# Stimulation of Redox‐Induced Electron Transfer by Interligand Hydrogen Bonding in a Cobalt Complex with Redox‐Active Guanidine Ligand

**DOI:** 10.1002/anie.202101423

**Published:** 2021-03-18

**Authors:** Lukas Lohmeyer, Florian Schön, Elisabeth Kaifer, Hans‐Jörg Himmel

**Affiliations:** ^1^ Anorganisch-Chemisches Institut Ruprecht-Karls-Universität Heidelberg Im Neuenheimer Feld 270 69120 Heidelberg Germany

**Keywords:** cobalt, electron transfer, guanidine, hydrogen bonding, redox active ligand

## Abstract

Octahedrally coordinated cobalt(II) complexes with a redox‐active bisguanidine ligand and acac co‐ligands were synthesized and their redox chemistry analysed in detail. The N−H functions in a bisguanidine ligand with partially alkylated guanidino groups form N−H⋅⋅⋅O hydrogen bonds with the acac co‐ligands, thereby massively influencing the redox chemistry. For all complexes, the first one‐electron oxidation is metal‐centred, leading to Co^III^ complexes with neutral bisguanidine ligand units. Further one‐electron oxidation is ligand‐centred in the case of Co–bisguanidine complexes with fully alkylated guanidino groups, giving Co^III^ complexes with radical monocationic bisguanidine ligands. On the other hand, the hydrogen‐bond strengthening upon oxidation of the Co–bisguanidine complex with partially alkylated guanidino groups initiates metal reduction (Co^III^→Co^II^) and two‐electron oxidation of the guanidine ligand, providing the first example for the stimulation of redox‐induced electron transfer by interligand hydrogen bonding.

## Graphical abstract

The strengthening of interligand hydrogen bonding triggers a redox‐induced intramolecular electron transfer (RIET) upon oxidation of a Co^III^ complex with a neutral redox‐active bisguanidine ligand, leading to metal reduction (Co^III^→Co^II^) and two‐electron oxidation of the bisguanidine ligand. In the absence of interligand hydrogen bonding, oxidation leads to a Co^III^ complex with a radical monocationic guanidine ligand.

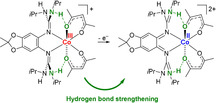

## Introduction

Cobalt complexes with redox‐active ligands have been the subject of intense research over the last decades,[^1^, ^2^, ^3^, ^4^, ^5^, ^6^] Especially, mononuclear complexes with one or two dioxolene‐type ligands and dinuclear complexes with a bridging tetraoxolene ligand were thoroughly studied, in part due to the huge potential of complexes with redox‐active ligands for catalysis.[^7^, ^8^, ^9^, ^10^, ^11^, ^12^, ^13^] Intramolecular electron transfer in such complexes could be triggered by various stimulants. In 1980, the first complex showing temperature‐dependent equilibrium between two redox isomers in solution was synthesized, converting a low‐spin Co^III^ complex preferred at low temperature into a high‐spin Co^II^ complex favoured at higher temperature.^[14]^ The term valence tautomerism (VT) was coined for equilibria between redox isomers. In 1993, the first complex showing VT in the solid state was reported,^[15]^ and several other examples followed,^[16]^ with various conversion temperatures, narrow or wide temperature regions for interconversion, and small or large thermal hysteresis.[^17^, ^18^] It proved possible to trigger intramolecular electron transfer (IET), converting a Co^III^ into a higher‐energetic Co^II^ complex by light at low temperature (similar to the light‐induced excited spin state trapping (LIESST) effect discovered by Gütlich et al.^[19]^) and also to stimulate relaxation back to the Co^III^ complex by light (similar to reverse‐LIESST).[^20^, ^21^, ^22^, ^23^, ^24^, ^25^] These studies gave useful information about the kinetics of IET, and highlighted lattice‐softening effects induced by co‐crystallized solvent molecules.^[25]^ Moreover, pressure‐induced VT was observed,^[26]^ and chain polymers were synthesized in which IET triggered by light could lead to measurable changes in the crystal length.^[27]^ It has also been shown that VT could initiate macroscopic crystal–melt phase transitions^[28]^ or change of polarization.^[29]^ Miller et al. studied the redox‐chemistry of dinuclear cobalt complexes with bridging tetraoxolene ligands, providing first examples for redox‐induced electron transfer (RIET).[^30^, ^31^, ^32^] In a RIET process, a redox reaction is coupled with intramolecular electron transfer, leading seemingly paradoxically to metal reduction in an overall oxidation or to metal oxidation in an overall reduction of a metal complex.

Although these extensive studies on Co–oxolene complexes greatly expanded our knowledge about the fundamentals of IET processes and disclosed a variety of different potential applications, there is still a high demand for studies in this field regarding both the fundamental understanding and the development of applications. Further redox‐active ligand classes have to be included into this research theme. In the last years, our group established redox‐active guanidines, comprising guanidino‐functionalized aromatics (GFAs), as a new class of versatile redox‐active ligands[^33^, ^34^, ^35^] and intensively studied IET in copper complexes with GFA ligands. We showed that IET could be triggered thermally (thermal equilibrium between two redox isomers (VT)),[^36^, ^37^, ^38^, ^39^] by redox reactions (redox‐induced electron transfer (RIET)),^[38]^ by co‐ligand addition[^40^, ^41^] or substitution,^[42]^ and by metal coordination to a secondary coordination sphere.^[43]^ Moreover, copper complexes with redox‐active guanidine ligands were applied in catalytic aerobic phenol homo‐ and cross‐coupling reactions,^[44]^ achieving a significant improvement of reactivity and selectivity.

**Figure 1:**
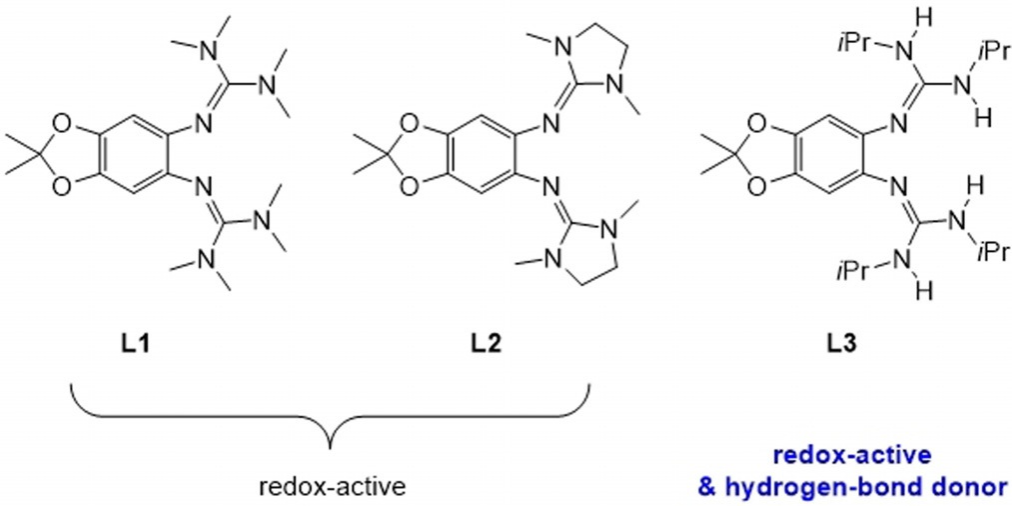
Lewis structures of the three redox‐active guanidine ligands L1–L3 used in this work. The hydrogen‐bond donor strength of L3 increases upon oxidation.

Herein, we report the first IET in a cobalt complex with a redox‐active bisguanidine ligand, being triggered in an unprecedented way by interligand hydrogen bonding. We used the three bisguanidines 5,6‐bis(*N*,*N*,*N*′,*N*′‐tetramethylguanidino)‐2,2‐dimethyl‐[1,3]‐benzodioxole (L1), 5,6‐bis(*N*,*N*′‐dimethyl‐*N*,*N*′‐ethylene‐guanidino)‐2,2‐dimethyl‐[1,3]‐benzodioxole (L2), and 5,6‐bis(*N*,*N*′‐diisopropyl‐guanidino)‐2,2‐dimethyl‐[1,3]‐benzodioxole (L3) sketched in Figure 1. L1 and L2 were previously reported;^[38]^ L3 is new and of key importance for this work due to its hydrogen‐bond donor properties that are enhanced upon oxidation. One‐ and two‐electron oxidation of Co^II^ complexes with one of the bisguanidines L1–L3 as ligand and two acetylacetonato (acac) co‐ligands led to stable complexes that were fully characterized. We present the first example for the initiation of RIET by hydrogen‐bond enforcement, enriching the arsenal of IET stimulants by a biomimetic, innovative alternative.

## Results and Discussion

**Scheme 1:**
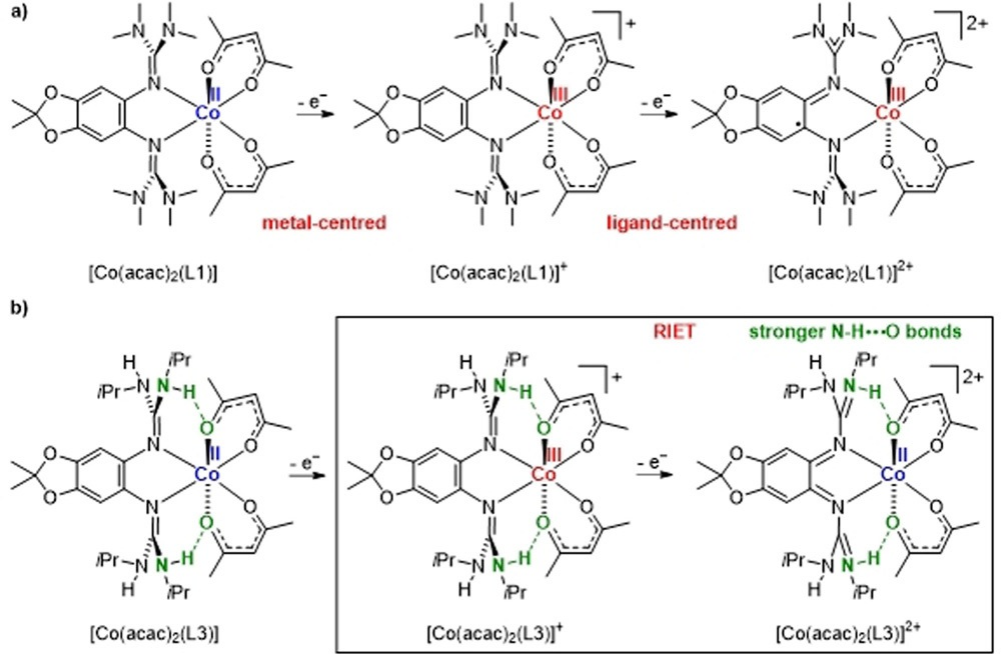
Comparison between stepwise oxidation of a) [Co(acac)_2_(L1)] and b) [Co(acac)_2_(L3)] in CH_2_Cl_2_ solution. The differences arise from a hydrogen‐bond triggered RIET process that is only feasible for the complex with L3.

The three neutral Co^II^ complexes [Co(acac)_2_(L1)], [Co(acac)_2_(L2)], and [Co(acac)_2_(L3)] (see Lewis structures of the complexes with L1 and L3 in Scheme 1) were synthesized in 93–98 % yield by reaction of one of the guanidine ligands L1–L3 with Co(acac)_2_ in CH_2_Cl_2_ solution, and crystallized from hot *n*‐hexane solutions. In difference to the complexes with L1 and L2, two N−H⋅⋅⋅O interligand hydrogen bonds between an N−H group of L3 and an O atom of the acac co‐ligands are established in [Co(acac)_2_(L3)] (see Lewis structure in Scheme 1). One‐electron oxidation was carried out with the ferrocenium (Fc^+^) salt Fc(PF_6_), and two electron oxidation either also with Fc(PF_6_) or with the stronger oxidant NO(SbF_6_). The complexes were isolated and fully characterized in all three redox states. The first one‐electron oxidation is metal‐centred (Co^II^→Co^III^) for all complexes. Further one‐electron oxidation of the complexes with L1 or L2 is ligand‐centred, giving Co^III^ complexes with radical monocationic ligand L1^.+^ or L2^.+^. On the other hand, oxidation of the monocationic complex [Co(acac)_2_(L3)]^+^ to the dication [Co(acac)_2_(L3)]^2+^ is coupled with IET (RIET) leading to cobalt reduction (Co^III^→Co^II^). RIET is triggered by the increase of the interligand N−H⋅⋅⋅O hydrogen‐bond strength upon two‐electron oxidation of the ligand. Hence detailed analysis clearly shows that the dication [Co(acac)_2_(L3)]^2+^ is a high‐spin Co^II^ complex with dicationic guanidine ligand. In principle, a further one‐electron oxidation is imaginable, leading to a tricationic Co^III^ complex with dicationic guanidine ligand. Indeed, cyclic voltammetry measurements indicate the possibility of reversible three‐electron oxidation (see below). However, it was not possible to isolate these ultimately oxidized complexes in pure form in experiments that relied on NO(SbF_6_) as oxidizing reagent. According to the experiments, partial degradation leads to cleavage of the bond between the metal and the dicationic guanidine in significant amount. In the following, the electronic structures of the neutral, as well as the singly and doubly oxidized complexes will be evaluated in detail. The discussion concentrates on the comparison between complexes of the ligands L1 and L3. The experimental results obtained for the complexes with L2 showed similar behaviour as the complexes of L1.

**Cyclic voltammetry**. For the free ligands L1 and L2, two reversible and potentially separated one‐electron processes are detected with cyclic voltammetry in CH_2_Cl_2_ solution,^[38]^ located at *E*_1/2_=−0.25 V (*E*_ox_=−0.19 V) and −0.11 V (*E*_ox_=−0.05 V) for L1^.+^/L1^0^ and L1^2+^/L1^.+^, respectively, and at *E*_1/2_=−0.46 V (*E*_ox_=−0.37 V) and −0.38 V (*E*_ox_=−0.27 V) for L2^.+^/L2^0^ and L2^2+^/L2^.+^, respectively. For free L3, the redox events are not reversible (SI, Figure S17); two oxidation waves appear, at *E*_ox_=−0.41 V and +0.05 V, tentatively assigned to oxidation of L3 to a hydrogen‐bonded dimer (L3^2+^)L3 (in equilibrium with small amounts of L3^.+^) and to L3^2+^, respectively. Three waves are visible in the direction of reduction. A wave at *E*_red_=−0.11 V belongs to one‐electron reduction of the dication (L3)^2+^. A smaller wave at −0.51 V and a larger one at −0.79 V are assigned to reduction of free (L3)^.+^ and of the (L3^2+^)L3 hydrogen‐bonded dimer, respectively. For tetrakisguanidino‐benzenes with partially alkylated guanidino groups, such aggregates were already synthesized in high yield and structurally characterized.[^45^, ^46^] Hence, the CV data of free L3 already point to the importance of hydrogen bonding.

**Figure 2:**
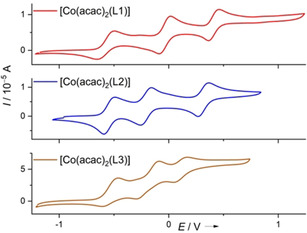
Cyclic voltammograms for the three neutral Co^II^ complexes in CH_2_Cl_2_ solutions (Ag/AgCl reference electrode, 0.1 m N(^*n*^Bu)_4_PF_6_ as supporting electrolyte, scan rate 20 mV s^−1^). Potentials given vs. the Fc^+^/Fc redox couple.

In the cyclic voltammogram recorded for [Co(acac)_2_(L1)] (Figure 2), three one‐electron redox processes are visible, at *E*_1/2_=−0.56 V for the redox couple [Co(acac)_2_(L1)]^+^/ [Co(acac)_2_(L1)]^0^, *E*_1/2_=−0.03 V for [Co(acac)_2_(L1)]^2+^/ [Co(acac)_2_(L1)]^+^, and *E*_1/2_=0.41 V for [Co(acac)_2_(L1)]^3+^/ [Co(acac)_2_(L1)]^2+^. Also, three redox processes are found for [Co(acac)_2_(L2)], at *E*_1/2_=−0.54 V for the redox couple [Co(acac)_2_(L2)]^+^/[Co(acac)_2_(L2)]^0^, *E*_1/2_=−0.21 V for [Co(acac)_2_(L2)]^2+^/[Co(acac)_2_(L2)]^+^, and *E*_1/2_=0.32 V for [Co(acac)_2_(L2)]^3+^/[Co(acac)_2_(L2)]^2+^. The potential for the first one‐electron oxidation is almost equal for [Co(acac)_2_(L1)] (*E*_1/2_=−0.56 V) and [Co(acac)_2_(L2)] (*E*_1/2_=−0.54 V), and significantly lower than the potentials required to oxidize the free ligands L1 (*E*_1/2_=−0.25 V for L1^.+^/L1^0^) or L2 (*E*_1/2_=−0.46 V for L2^.+^/L2^0^). These results clearly indicate that the first one‐electron oxidation is metal‐centred (Co^II^→Co^III^), as expressed by the Lewis structures in Scheme 1 a. It should be noted that at fast scan rates (more than 50 mV s^−1^) a small feature is visible on the high‐potential side of the first oxidation wave for all three complexes, vanishing at slower scan rates. The disappearance of this feature at slow scan rates and the similar behaviour of all three compounds argue against the presence of a compound with different composition in the solutions. The change from high‐spin Co^II^ to low‐spin Co^III^ brings about massive structural and electronic changes, and therefore it might be possible that the presence of an intermediate complex (e.g. a Co^III^ complex with different spin multiplicity that relaxes to the low‐spin Co^III^ ground state) causes the additional weak features at high scan rates.

The *E*_1/2_ values measured for the second oxidation process, −0.03 V for [Co(acac)_2_(L1)]^2+^/[Co(acac)_2_(L1)]^+^ and −0.21 V for [Co(acac)_2_(L2)]^2+^/[Co(acac)_2_(L2)]^+^, are both higher than the potentials for one‐electron oxidation of the free ligands. Such an increase could easily be explained by the bonding to the Lewis‐acidic metal. Moreover, the difference in the *E*_1/2_ values of 0.18 V is almost equal to the difference of 0.21 V for the free ligands. On these grounds, the second one‐electron oxidation is assigned to a ligand‐centred oxidation process, leading to Co^III^ complexes with radical monocationic guanidine ligands (Scheme 1 a). The third oxidation process should then produce Co^III^ complexes with dicationic guanidine ligand. The potentials for one‐ and two‐electron oxidation of the complexed ligands (being the second and third oxidation steps of the complex) are clearly potentially separated (Δ*E*_1/2_=0.44 V for L1 and 0.53 V for L2), comparing with Δ*E*_1/2_=0.14 and 0.08 V for the free ligands L1 and L2, respectively.

The cyclic voltammogram of [Co(acac)_2_(L3)] is also included in Figure 2. Here, also three redox waves appear, at *E*_1/2_=−0.53 V (*E*_ox_=−0.46 V), *E*_1/2_=−0.19 V (*E*_ox_=−0.09 V), and *E*_1/2_=+0.11 V (*E*_ox_=+0.17 V), assigned to the couples [Co(acac)_2_(L3)]^+^/[Co(acac)_2_(L3)]^0^, [Co(acac)_2_(L3)]^2+^/[Co(acac)_2_(L3)]^+^, and [Co(acac)_2_(L3)]^3+^/[Co(acac)_2_(L3)]^2+^, respectively. The potential of the first one‐electron process is close to those measured for the analogue complexes with L1 and L2, and lower than the potential measured for the free ligand L3; therefore it is assigned to a metal‐centred oxidation (Co^II^→Co^III^).

The second and third one‐electron redox events then belong to ligand‐centred oxidation. We will see that the second redox event is accompanied by a RIET process (from Co^III^ to Co^II^, see Scheme 1). The potential difference between the oxidation and reduction wave of the redox pair [Co(acac)_2_(L3)]^2+^/[Co(acac)_2_(L3)]^+^ is large (0.22 V), possibly due to the significant structural changes induced by the RIET process. From the synthesis of stable salts of the monocation [Co(acac)_2_L3]^+^ and dication [Co(acac)_2_L3]^2+^ by chemical oxidation of the neutral compound (see below) an inherent instability of the redox states could be excluded. The CV measurements suggest the third one‐electron redox process, leading to a Co^III^ complex with dicationic guanidine ligand, to be reversible. However, it was not possible to synthesize salts of these complexes in pure form.

**Figure 3:**
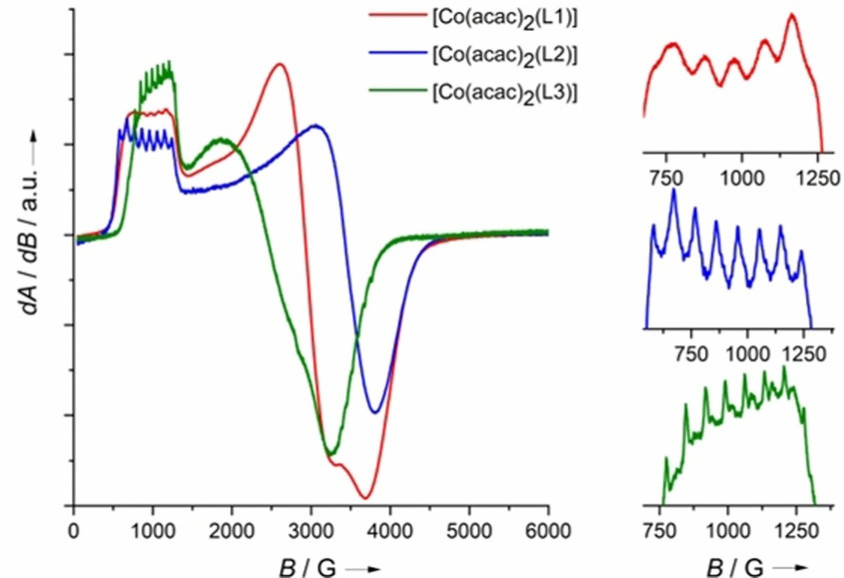
EPR spectra (9.63 GHz) of [Co(acac)_2_(L1)] (red), [Co(acac)_2_(L2)] (blue) and [Co(acac)_2_(L3)] (green) at 6 K in frozen CH_2_Cl_2_.

**EPR spectroscopy**. EPR spectra of the three neutral complexes, measured at 6 K in frozen CH_2_Cl_2_ solution, are displayed in Figure 3. The signals in the EPR spectra vanish at room temperature due signal broadening by fast relaxation processes; all data clearly show that Co^II^ complexes with neutral guanidine ligands prevail at all temperatures. The g values of g_⊥_=6.99 and g_∥_=2.34 give an effective g value of 3.89 for the complex [Co(acac)_2_(L1)]. For [Co(acac)_2_(L2)], g_⊥_=7.53 and g_∥_=2.00 add up to an effective g value is 3.84. Values of g_⊥_=6.66 and g_∥_=2.68 are obtained for [Co(acac)_2_(L3)], resulting in an effective g value of 4.00. Hyperfine coupling to the nuclear spin of the ^59^Co nucleus (*I*=7/2) should produce splitting into eight lines. Indeed, eight lines are visible for [Co(acac)_2_(L2)] (Figure 3). Only five of them are visible for [Co(acac)_2_(L1)], the others being buried. The coupling constants for [Co(acac)_2_(L1)] (98 G) and for [Co(acac)_2_(L2)] (95 G) are very similar. For [Co(acac)_2_(L3)], eight sharp lines show, but also additional lines due to coupling with other nuclei. With 72 G, the A value is slightly smaller, presumably due to the structural peculiarities produced by the two hydrogen bonds (see structure below).

**Figure 4:**
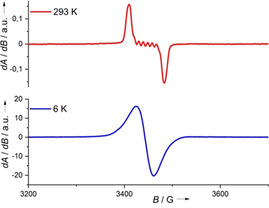
EPR spectra (9.63 GHz) of [Co(acac)_2_(L1)](PF_6_)_2_ in CH_2_Cl_2_ at 6 K and at 293 K.

No EPR signal was detected for [Co(acac)_2_(L1)](PF_6_) in CH_2_Cl_2_, indicating that [Co(acac)_2_(L1)]^+^ is a diamagnetic Co^III^ complex with neutral ligand in the whole temperature region 6–300 K. Complementary with these results, signals appeared in the ^1^H NMR spectrum of the compound (see. SI, Figure S3). The EPR spectrum of [Co(acac)_2_(L1)](PF_6_)_2_ in a frozen CH_2_Cl_2_ solution at 6 K shows a sharp signal due to a ligand‐centred radical with a g value near 2 (1.997). At 293 K, a clear hyperfine structure due to isotropic hyperfine coupling with the ^59^Co nucleus is visible, resulting in line splitting with a hyperfine‐coupling constant A=97 G (Figure 4). Similar EPR spectra were recorded for [Co(acac)_2_(L2)](PF_6_)_2_ (SI, Figure S25). Hence, [Co(acac)_2_(L1)]^2+^ and [Co(acac)_2_(L2)]^2+^ are Co^III^ complexes with radical monocationic ligands, in line with the results from cyclic voltammetry and the Lewis structures in Scheme 1.

On the other hand, no sharp signal appears in the room‐temperature EPR spectrum of [Co(acac)_2_(L3)](PF_6_)_2_, confirming the absence of an organic radical and the formulation as a Co^II^ complex with dicationic ligand unit L3^2+^. In similarity to the neutral complexes, the absence of clear signals could be rationalized by signal broadening due to fast relaxation at room temperature. Interestingly, a weak and broad signal that might be assignable to an organic radical appeared in the 6 K EPR spectrum, together with weak, broad signals in the region 550–3000 G (SI, Figure S26). In principal, we could not completely exclude that these signals arise from the presence of a minor impurity, but their absence in the room temperature spectra seems to be not in accordance with this explanation. Another possibility is a change in the electronic structure of the [Co(acac)_2_(L3)]^2+^ complex at very low temperature, that is also motivated by the calculated small energy difference between two redox isomers (see discussion below).

**UV/Vis spectroscopy**. In the UV/Vis spectra of the free ligands in CH_3_CN solution, bands at 334 and 300 nm belong to L1, bands at 336 and 277 nm to L2,^[38]^ and strong bands around 340 and 234 nm as well as a small shoulder around 265 nm to L3 (see SI, Figure S4). Bands around 450 and 300 nm were observed for the dications L1^2+^ and L2^2+^.^[38]^ Then, the free radical monocationic ligand L1^.+^ exhibits a band at 370 nm with a long tail extending into the visible region. A band at ca. 370 nm (with a shoulder around 385 m) is also characteristic for free L2^.+^, together with a broad band in the vis region (absorption maxima at 675/733 nm).

In the UV/Vis spectra of the neutral cobalt complexes, strong absorptions appear in the UV region, but only an unstructured, weak, and extremely broad absorption (400–500 nm) in the visible region, being responsible for the red colour of the compounds. The visible regions in the spectra of the monocations [Co(acac)_2_(L1)]^+^, [Co(acac)_2_(L2)]^+^, and [Co(acac)_2_(L3)]^+^ are also free of strong bands, arguing for metal‐centred oxidation (Co^II^→Co^III^), in line with the results from cyclic voltammetry and EPR spectroscopy. The spectra recorded for the dications [Co(acac)_2_(L1)]^2+^ and [Co(acac)_2_(L2)]^2+^ obtained upon two‐electron oxidation contain a band at 364 nm and a broad absorption in the visible region with an absorption maximum at ca. 554 nm for [Co(acac)_2_(L1)]^2+^ and 503 nm for [Co(acac)_2_(L2)]^2+^, indicating the presence of a Co^III^ complex with radical monocationic ligand. On the other hand, bands at 295 and 450 nm in the spectrum of [Co(acac)_2_(L3)]^2+^ in CH_2_Cl_2_ (being close to the absorptions detected for similar oxidized, dicationic bisguanidines^[38]^) clearly indicate the presence of the dicationic ligand, L3^2+^, implying the presence of a Co^II^ complex. In summary, the UV/Vis spectra are in line with cyclic voltammetry, EPR, and Lewis structures in Scheme 1.

**Figure 5:**
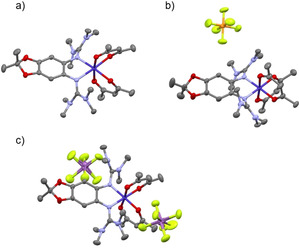
Illustration of the structures of a) [Co(acac)_2_(L1)], b) [Co(acac)_2_(L1)](PF_6_), and c) [Co(acac)_2_(L1)](SbF_6_)_2_ in the solid state. Color code: Co dark blue, N pale blue, O red, C dark grey, H pale grey, F pale green, P orange, Sb pink. Displacement ellipsoids drawn at the 50 % probability level. All hydrogen atoms omitted.

**Crystal structures**. The crystal structure of [Co(acac)_2_(L1)] is displayed in Figure 5, that of [Co(acac)_2_(L2)] is shown in the SI (Figure S31). In both compounds, the bisguanidine ligand binds with its two imino N atoms to the octahedrally coordinated Co^II^ atom. The Co−N bond lengths measure 2.135(2)/2.149(2) Å for [Co(acac)_2_(L1)] and 2.085(2)/2.245(2) for [Co(acac)_2_(L2)]. Due to combined σ‐ and π‐contributions to the metal−guanidine bonding,^[47]^ the N=C bond lengths increase from 1.295(1)/1.292(1) Å and 1.293(2)/1.287(2) Å in free L1 and L2, respectively,^[38]^ to 1.325(3)/1.318(3) Å and 1.326(2)/1.311(2) Å in [Co(acac)_2_(L1)] and [Co(acac)_2_(L2)], respectively.

The Co−N and Co−O bond lengths decrease significantly from neutral [Co(acac)_2_(L1)] to the monocation [Co(acac)_2_(L1)]^+^ and the dication [Co(acac)_2_(L1)]^2+^ (Figure 5 and Table 1), confirming conversion from a neutral high‐spin Co^II^ complex (*S*=3/2) to a low‐spin Co^III^ complex upon oxidation. The decrease of the Co−N bond lengths (by 0.183/0.189 Å) and Co−O bond lengths (by 0.163/0.187 Å) after two‐electron oxidation add up to an average bond length decrease of 0.180 Å, being similar to the value reported for VT between Co^II^ and Co^III^ complexes with two oxolene ligand units.^[1]^ The C1−C2 bond length remains short after one‐electron oxidation (1.405(5) Å), but significantly increases after two‐electron oxidation (to 1.440(4) Å), signalling loss of aromaticity in the second, ligand‐centred oxidation step. The N=C double bonds in the neutral Co^II^ complexes with reduced, neutral L1, measuring in average 1.321 Å, are elongated to an average value of 1.382 Å upon two‐electron oxidation. The N−C bonds connecting the guanidino groups to the C_6_ ring decrease from 1.408 Å before to 1.362 Å after two‐electron oxidation. In summary, the crystal structures fully support the Lewis structures in Scheme 1.

**Table 1:** Selected bond lengths [in Å] for structurally characterized complexes [Co(acac)_2_(L1)]^*n*+^ (*n*=0, 1, or 2) in the solid state.^[48]^

bond	n=0	n=1	n=2	
N1−Co1	2.135(2)	1.960(3)	1.952(2)	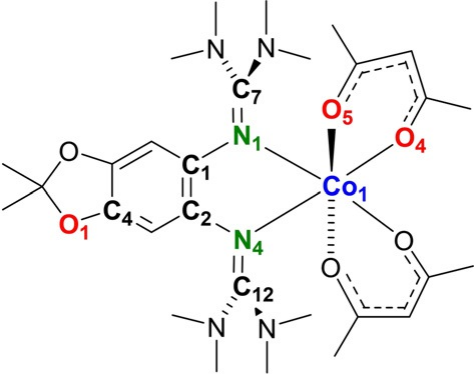
N4−Co1	2.149(2)	1.965(3)	1.960(2)	
Co1−O5	2.059(1)	1.896(3)	1.896(2)	
Co1−O4	2.077(1)	1.888(3)	1.890(2)	
C1−N1	1.410(2)	1.404(5)	1.359(4)	
C2−N4	1.406(2)	1.424(5)	1.365(4)	
N1−C7	1.325(3)	1.347(5)	1.382(4)	
N4−C12	1.318(3)	1.342(5)	1.382(4)	
C1−C2	1.411(3)	1.405(5)	1.440(4)	
C4−O1	1.389(2)	1.383(5)	1.352(4)	

**Figure 6:**
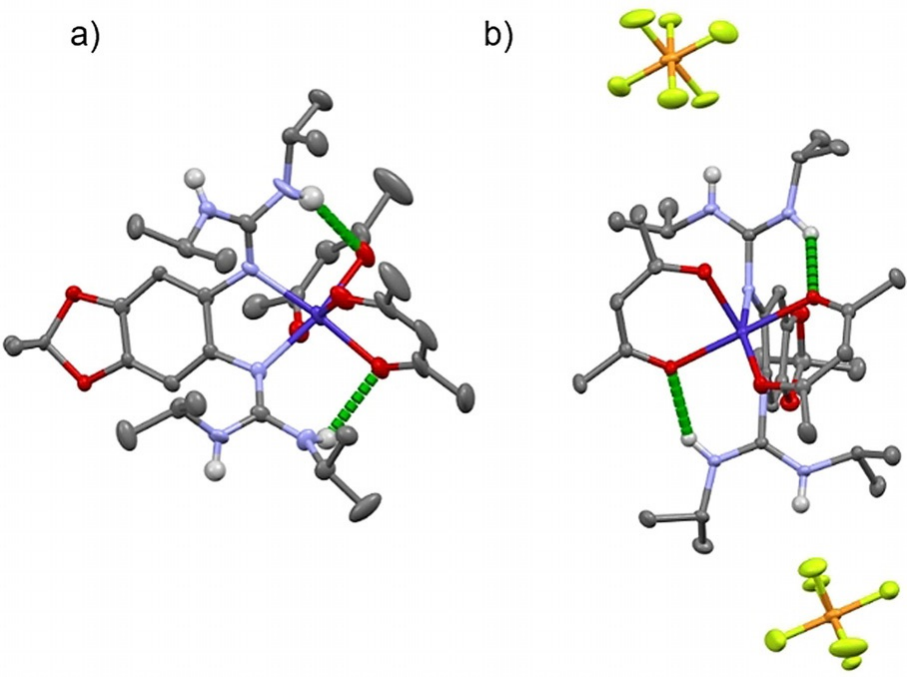
Illustration of the structures of a) [Co(acac)_2_(L3)] and b) [Co(acac)_2_(L3)](PF_6_)_2_ in the solid state. Color code: Co dark blue, N pale blue, O red, C dark grey, H pale grey, F pale green, P orange. Displacement ellipsoids drawn at the 50 % probability level. Hydrogen atoms bound to carbon omitted. The interligand hydrogen bonds are highlighted in green.

Figure 6 displays the crystal structures of the neutral and dicationic complexes of L3, [Co(acac)_2_(L3)], and [Co(acac)_2_(L3)](PF_6_)_2_. The Co–N distances slightly increase, from average 2.150 Å before to 2.175 Å after oxidation, and the Co–O distances slightly decrease, from average 2.083 Å before to 2.043 Å after oxidation, but these values remain in the region typical for Co^II^ (Table 2), in sharp contrast to the changes upon oxidation of the complex with L1. Moreover, the bond lengths within the L3 ligand unit clearly indicate ligand oxidation (e.g. massive increase of the N1−C7/N4−C12 and decrease of the C1−N1/C2−N4 bond lengths). Hence, [Co(acac)_2_(L3)]^2+^ is best described as a Co^II^ complex with dicationic ligand unit L3^2+^. To obtain structural information about the hydrogen atoms within the hydrogen bonds, the positions of the relevant hydrogens in [Co(acac)_2_(L3)]^2+^ and [Co(acac)_2_(L3)]^2+^ were taken from difference Fourier maps and freely refined. The experimental NH⋅⋅⋅O distances decrease from 2.22(6)/2.15(6) Å in [Co(acac)_2_(L3)] to 2.020(2) Å in [Co(acac)_2_(L3)]^2+^. The more reliable distances between the N and O atoms within the N−H⋅⋅⋅O hydrogen bonds decrease from 3.033(9)/2.966(8) Å in [Co(acac)_2_(L3)] to 2.762(2) Å in [Co(acac)_2_(L3)]^2+^ (see SI), a value that is quite small in comparison to previously reported interligand N−H⋅⋅⋅O hydrogen bonds.[^49^, ^50^] The strengthening (and harmonization) of the hydrogen bonds upon oxidation, as suggested by the experimental solid‐state structures, is also backed by quantum‐chemical calculations giving similar values for the N⋅⋅⋅O distances (see below). Since RIET is only observed for the complex with L3, it could be concluded that the hydrogen‐bond strengthening triggers IET from the guanidine ligand unit to the cobalt atom upon oxidation. Quantum‐chemical calculations (see discussion below) support this conclusion.

**Table 2:** Selected bond lengths [in Å] and angles [in °] for the structurally characterized complexes [Co(acac)_2_(L3)]^*n*+^ (*n*=0 or 2) in the solid state.^[48]^

bond/ angle	*n*=0	*n*=2	
N1−Co1	2.161(4)	2.175(1)	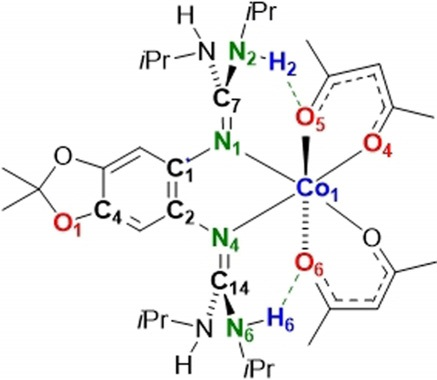
N4−Co1	2.138(6)	2.175(1)	
Co1−O5	2.059(6)	2.021(1)	
Co1−O4	2.106(4)	2.065(1)	
C1−N1	1.410(8)	1.297(2)	
C2−N4	1.402(7)	1.297(2)	
N1−C7	1.308(7)	1.408(2)	
N4−C12	1.330(1)	1.408(2)	
C1−C2	1.417(9)	1.514(3)	
C4−O1	1.391(8)	1.331(2)	
H2−O5	2.15(6)	2.020(2)	
H6−O6	2.22(6)	2.020(2)	
N2−O5	2.966(8)	2.762(2)	
N6−O6	3.033(9)	2.762(2)	
∡ N2‐H2‐O5	156(5)	152(2)	
∡ N6‐H6‐O6	154(4)	152(2)	

**Figure 7:**
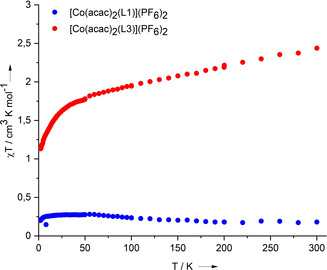
Plot of the magnetometric (SQUID) data for [Co(acac)_2_(L1)](PF_6_)_2_ (blue) and [Co(acac)_2_(L3)](PF_6_)_2_ (red) in the temperature range 2–300 K, measured at 50 mT.

**SQUID measurements**. Magnetometric (SQUID) data were measured for [Co(acac)_2_(L1)](PF_6_)_2_ and [Co(acac)_2_(L3)](PF_6_)_2_ to confirm the differences in the electronic structure (Figure 7). For [Co(acac)_2_(L1)](PF_6_)_2_, the *χ T* value measures 0.181 cm^3^ K mol^−1^ at 300 K and slightly increases to 0.276 cm^3^ K mol^−1^ at 40 K, being close to the theoretical value of 0.375 cm^3^ K mol^−1^ obtained from the Curie law for a compound with one unpaired electron (*S*=1/2). Hence, the data are in line with the presence of low‐spin Co^III^ and L1^.+^.

A much higher *χ T* value of ca. 2.5 cm^3^ K mol^−1^ was found for [Co(acac)_2_(L3)](PF_6_)_2_ at 300 K. This large value fully supports the formulation as a high‐spin Co^II^ complex (*S*=3/2) with dicationic ligand, L3^2+^ and an unquenched orbital contribution. Due to significant spin‐orbit coupling (^4^T_1g_ ground term in *O_h_* symmetry), it is higher than the spin‐only value for high‐spin Co^II^ (*S*=3/2) of 1.876 cm^3^ K mol^−1^ predicted by the Curie law. The value decreases almost linearly with decreasing temperature until ca. 50 K (Figure 7), where it drops down to reach a value of 1.1 cm^3^ K mol^−1^ at 2 K. The sharp decrease at low temperature^[51]^ is characteristic for octahedral high‐spin Co^II^ complexes.[^52^, ^53^] The degeneracy of the ^4^T_g_ ground state is removed by spin–orbit coupling and the distortion of the crystal field due to the asymmetric coordination of two different ligands. According to the Boltzmann distribution, the population of the resulting doublet ground state increases with decreasing temperature, leading to reduction of the *χ T* value at low temperature.^[52]^ Additionally, a possible VT process favouring the Co^III^ redox isomer with L3^.+^ ligand unit at very low temperature might lead to a further decrease of the *χ T* value. The latter is supported by the appearance of a weak, broad signal attributable to an organic radical in the low temperature (6 K) EPR spectrum of a frozen CH_2_Cl_2_ solution (SI, Figure S26), and by the calculated small energy difference between two redox isomers (see below). However, it is not possible to reach a final conclusion on this point.

**Figure 8:**
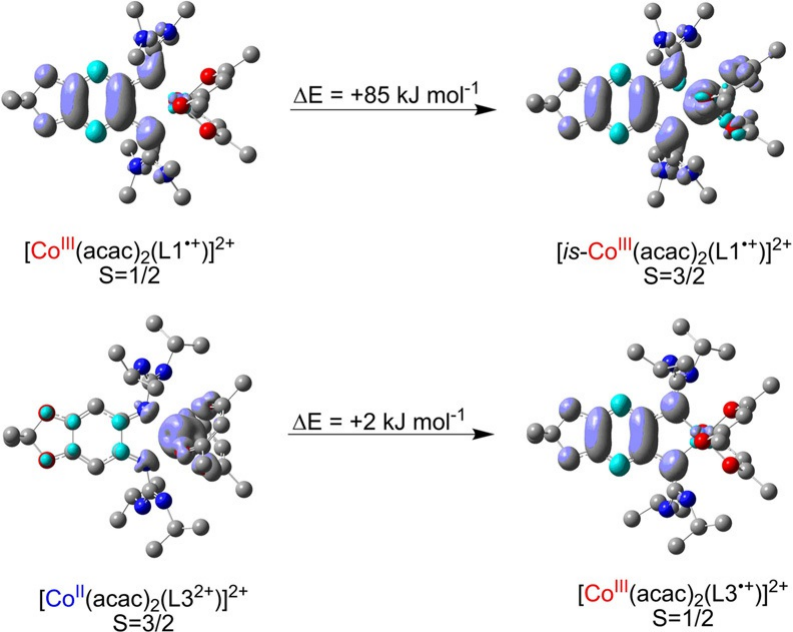
Visualization of the spin density and relative energies (including zero‐point energy) from B3LYP/def2‐TZVP calculations for the two redox isomers of [Co(acac)_2_(L1)]^2+^ and [Co(acac)_2_(L3)]^2+^ at *ϵ*_r_=37.5. Hydrogen atoms omitted. The calculations for [Co(acac)_2_(L1)]^2+^ and *S*=3/2 converged to the intermediate‐spin (*is*) Co^III^ complex with L1^.+^ instead of a high‐spin Co^II^ complex with L1^2+^.

**Quantum‐chemical calculations**. Finally, B3LYP/def2‐TZVP calculations were carried out, the solvent effect being included by single‐point calculations with the conductor‐like screening model (COSMO). The calculated structures are in pleasing agreement with the experimentally derived structures in the solid state (see SI for details). In Figure 8 the spin density distribution is plotted for [Co(acac)_2_(L1)]^2+^ and [Co(acac)_2_(L3)]^2+^. The calculations for the quartet state of [Co(acac)_2_(L1)]^2+^ did not converge to a Co^II^ complex with dicationic ligand L1^2+^, but instead to an intermediate‐spin Co^III^ complex with radical monocationic ligand L1^.+^ (see Table 3). The low‐spin Co^III^ redox isomer with radical monocationic ligand L1^.+^ (*S*=1/2) is preferred by Δ*E*=85 kJ mol^−1^ at *ϵ*_r_=37.5 with respect to this intermediate‐spin Co^III^ redox isomer.

**Table 3:** Calculated natural spin population (B3LYP/def2‐TZVP) for the two redox isomers of [Co(acac)_2_(L1)]^2+^ and [Co(acac)_2_(L3)]^2+^.

fragment	Co	(acac)_2_	L1/L3
[Co^III^(acac)_2_(L1^+^)]^2+^	0.006	0.012	0.982
[*is*‐Co^III^(acac)_2_(L1^+^)]^2+^	1.800	0.153	1.047
[Co^III^(acac)_2_(L3^+^)]^2+^	0.018	0.015	0.966
[Co^II^(acac)_2_(L3^2+^)]^2+^	2.720	0.288	−0.008

In the calculated Co^II^ redox isomer of [Co(acac)_2_(L3)]^2+^ (*S*=3/2) with dicationic ligand, L3^2+^, the N−H⋅⋅⋅O hydrogen bond lengths measure 1.824 Å, being significantly shorter than in the calculated Co^III^ redox isomer with radical monocationic L3^.+^ unit (2.061 Å). Additionally, the calculated distances between the N and the O atom in the N−H⋅⋅⋅O hydrogen bonds in the neutral and the dicationic complexes of L3 are similar to those found in the corresponding crystal structures. Hence experimental values of 3.033/2.966 and 2.762 Å were found for [Co(acac)_2_(L3)] and [Co(acac)_2_(L3)]^2+^, respectively, being close to the calculated values of 2.905/2.835 and 2.788 Å (see SI). The decrease of the hydrogen‐bond lengths signals a stronger interligand interaction, leading to a stabilization of the Co^II^ redox isomer by the two strong interligand hydrogen bonds. Consequently, the calculations predict the otherwise highly unfavourable quartet spin state (being high‐energy states for complexes of L1 and L2) to be even slightly preferred over the doublet state (by 2 kJ mol^−1^) at *ϵ*_r_=37.5 (CH_3_CN solution); without solvent effect the doublet state (Co^III^ redox isomer) is slightly preferred, but by not more than 10 kJ mol^−1^.

**Figure 9:**
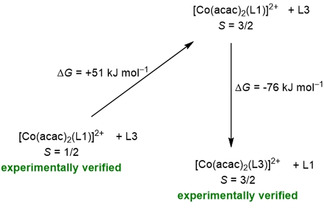
Gibbs free energy change (at 298 K, 1 bar, without inclusion of the solvent effect) for substitution of the L1 ligand in [Co(acac)(L1)]^2+^ (after redox‐isomerization to the *S*=3/2 state) by the hydrogen‐bond donor ligand L3 from B3LYP/def2‐TZVP calculations.

The Gibbs free energy for a hypothetical ligand exchange reaction was calculated to further highlight the impact of hydrogen bonding (Figure 9). For the reaction of [Co(acac)_2_(L1)]^2+^ in its *S*=3/2 state with free L3 to give [Co(acac)_2_(L3)]^2+^ in the *S*=3/2 ground state and free L1, a Δ*G* value (at 298 K) of −76 kJ mol^−1^ was obtained, underlining the massive stabilization of the *S*=3/2 state (Co^II^ redox isomer) with respect to the *S*=1/2 state (Co^III^ redox isomer) for the complex with L3 by hydrogen‐bonding.

## Conclusion

The first mononuclear, octahedrally coordinated cobalt complexes with redox‐active bisguanidine ligands were synthesized and their redox chemistry was studied. Three different redox‐active bisguanidines were used in this study, two of them with fully alkylated and one with partially alkylated guanidino groups. Interligand N−H⋅⋅⋅O hydrogen bonds between two guanidino N−H functions and oxygen atoms of the acetylacetonate (acac) co‐ligands are established in the Co–bisguanidine complex with partially alkylated guanidino groups. The increase of the hydrogen‐bond strength upon oxidation of this guanidine ligand triggers a redox‐induced electron transfer (RIET) that is absent in the complexes with the guanidine ligands having fully alkylated guanidino groups. Hence, one‐electron oxidation of the monocationic Co^III^ complex with the neutral guanidine ligand leads to metal reduction and two‐electron oxidation of the guanidine ligand, resulting in a Co^II^ complex with dicationic guanidine ligand unit. The results of this study demonstrate the possibility to alter the outcome of redox processes and to initiate intramolecular electron transfer (IET) by introducing intramolecular hydrogen‐bond interactions. Thereby, they pave the way to a sophisticated control of redox and IET processes. Nature extensively uses the weakening or strengthening of hydrogen bonding up to the point of proton transfer to trigger electron transfer,^[54]^ for example, in a variety of redox enzymes. Therefore, the development of model reactions in which electron transfer is initiated by hydrogen bonding leads to the realization of new biomimetic redox reaction motifs. The first example for a RIET enabled by interligand hydrogen bonding discloses new opportunities for the systematic study of intramolecular electron transfer, leading eventually to new reactivity patterns for use in synthetic chemistry.

## Conflict of interest

The authors declare no conflict of interest.

## Supporting information

As a service to our authors and readers, this journal provides supporting information supplied by the authors. Such materials are peer reviewed and may be re‐organized for online delivery, but are not copy‐edited or typeset. Technical support issues arising from supporting information (other than missing files) should be addressed to the authors.

Supplementary
