# Supplementary material for: Stimulation of Redox‐Induced Electron Transfer by Interligand Hydrogen Bonding in a Cobalt Complex with Redox‐Active Guanidine Ligand
Source: Angew Chem Int Ed Engl. 2021 Mar 18;60(18):10415–22. doi: 10.1002/anie.202101423 (PMC8252010; doi:10.1002/anie.202101423)
Supplement: Supplementary file 1 — Supplementary [file ANIE-60-10415-s001.pdf]

Angewandte Chemie
Eine Zeitschrift der Gesellschaft Deutscher Chemiker

Supporting Information

Stimulation of Redox-Induced Electron Transfer by Interligand Hydrogen Bonding in a Cobalt Complex with Redox-Active Guanidine Ligand

*Lukas Lohmeyer, Florian Schön, Elisabeth Kaifer, and Hans-Jörg Himmel**

anie_202101423_sm_miscellaneous_information.pdf

Content

1. General information **2**
2. Experimental procedures **2**
  2.1 Neutral Co(II)-complexes 3
  2.2 Single-oxidized complexes 4
  2.3 Twofold-oxidized complexes 5
3. Analytical data **8**
  3.1 NMR spectra 8
  3.2 UV-vis spectra 10
  3.4 EPR spectroscopy 21
  3.5 SQUID measurements 26
4 Crystallographic data **27**
  4.1 Details of the structural characterizations. 27
  4.2 Crystal structures 28
  4.2 Details of the crystal structure determinations 34
5 DFT calculations **36**
  5.1 Calculated structures and spin densities 36
  5.2 Comparison of H-bond lengths of calculated and measured structures 46
  5.3 Calculations of the Gibbs free energy for ligand exchange reactions 46
  5.4 aRMSD calculations 47
  5.4 Cartesian coordinates for all complexes, calculated with B3LYP/def2-TZVP 57
6 References **77**

1. General information

If not stated otherwise, all reactions were carried out under a dry argon atmosphere using standard Schlenk techniques or in a dinitrogen-filled glove box (MBraun, LABstar). The applied solvents were dried with an MBraun Solvent Purification System, degassed by three freeze-pump-thaw cycles and stored over molecular sieves prior to their use. The ligands L1 and L2 as well as 5,6-diamino-2,2-dimethyl-[1,3]-benzodioxol-dihydrochloride were synthesized according to the literature.[S1] The reagents cobalt(II) acetylacetonate, ferrocenium hexafluorophosphate, $Zn(OTf)_2$ nitrosyl hexafluoroantimonate and diisopropylcarbodiimide were purchased from Sigma Aldrich, abcr and TCI and used without further purification. Elemental analysis was performed at the Microanalytical Laboratory of the University of Heidelberg using the vario EL and vario MICRO cube devices from Elementar Analysensysteme GmbH. NMR spectra were recorded on a Bruker Avance II 400 spectrometer or Bruker Avance III 600 spectrometer at a temperature of 298 K. Solvent resonances were taken as references for all ^{1}H NMR or ^{13}C NMR spectra if not stated otherwise, NMR spectra were recorded at 298 K. UV/Vis spectra were measured on a VARIN Cary 5000 UV-Vis-NIR spectrophotometer. CV measurements were carried out with a Metrohm Autolab PGSTAT 204 potentiostate/galvanostate and an Ag/AgCl reference electrode, Pt rod counter electrode and glassy carbon working electrode. The curves were recorded at room temperature with different scan rates (30, 100, 200 $m{\cdot}V{\cdot}s^{-1}$). CH_2Cl_2 was used as solvent for the individual compounds ($c = 10^{-3}$ M), whereas $n$$Bu_4N(PF_6)$ was employed as supporting electrolyte ($c = 0.1$ M). Infrared spectra were recorded as solids on an ATR crystal with an AGILENT Cary 630 FTIR spectrometer. X-band EPR spectra were measured with a Bruker Elexsys E500 EPR with an ER 4116DM CW dual mode resonator. The solid samples and r.t. measurements in solution are prepared in thin glass tubes (Ø ≈ 1 mm). As temperature system a ER 4112HV-CF58nc In-Cavaty Cryogen Free VT is used. The dc-SQUID measurements were taken with a MPMS-XL apparatus from LOT Quantum Design.

2. Experimental procedures

L3: Diisopropylcarbodiimide (1.7 g, 13.5 mmol) was added to a suspension of 5,6-diamino-2,2-dimethyl-[1,3]-benzodioxol-dihydrochloride (1.69 g, 6.68 mmol) and

$Zn(OTf)_2$ (15.5 g, 34.0 mmol) in THF (40 mL) and the reaction mixture stirred for six days at room temperature. The beige crude product was filtrated, washed with Et_2O (2 x 5 ml) and dried under reduced pressure. A solution of NaOMe in MeOH (4 ml, 5.4 M) was added and the resulting solution was stirred for 15 min. The solvent was removed in vacuo and the resulting grey-brown solid was washed with H_2O (3 x 15 ml) and *n*-hexane (8 ml) to yield 1.90 g (66%, 4.39 mmol) of the product as a beige solid. Crystals suitable for structural characterization by single-crystal X-ray diffraction were grown from a saturated acetonitrile solution. C,H,N analysis (%) for $C_{23}H_{40}N_6O_2$: calcd. C 63.86, H 9.32, N 19.43; found C 63.55, H 9.44, N 19.85. ^{1}H NMR (400 MHz, CD_3CN): δ = 6.61 (s, 2 H), 4.03 (br s, 4 H), 3.71 (br s, 4 H), 1.60 (s, 6H), 1.08 (s, 12H), 1.07 (s, 12H) ppm. ^{13}C NMR (100 MHz, CD_3CN): δ = 150.19, 143.17, 118.30, 117.56, 105.32, 43.61, 25.84, 23.62 ppm. MS (ESI$^+$): m/z (%) 433.32 (100) [M+H$^+$]. UV-vis (CH_2Cl_2): λ_{max} (ε in L·mol^{-1} cm^{-1}) = 231 (1.97·10^4), 266 (0.79·10^4), 324 (0.84·10^4) nm. IR (ATR) $\tilde{\nu}$ (cm^{-1}) = 3418w, 3355w, 3205w, 2963m, 2929w, 2865w, 1620s, 1596s, 1520m, 1475vs, 1402w, 1380m, 1361s, 1289w, 1242m, 1214m, 1181s, 1168s, 1149s, 1125s, 1049m, 1023w, 975s, 928w, 874m, 863m, 845s, 788m, 772m, 708m, 656w.

2.1 Neutral Co(II)-complexes

[Co(acac)$_2$(L1)]: L1 (60 mg, 0.16 mmol) was dissolved in 10 mL of CH_2Cl_2. Then $Co(acac)_2$ (41 mg, 0.16 mmol) was added in one portion. The colour of the solution immediately changed to light-red. After 2 h of stirring the solvent was removed in vacuo and the crude product was dissolved in 20 mL of *n*-hexane. The solution was filtrated to remove excess $Co(acac)_2$ and dried in vacuo to yield 99 mg (98% 0.16 mmol) of [Co(acac)$_2$(L1)] as a dusky-pink solid. Crystals suitable for structural characterization by single-crystal X-ray diffraction were grown from a hot saturated *n*-hexane solution. C,H,N analysis (%) for $C_{29}H_{46}CoN_6O_6$: calcd. C 54.97, H 7.32, N 13.26; found C 54.89, H 7.48, N 13.26. UV-vis (CH_2Cl_2): λ_{max} (ε in L·mol^{-1} cm^{-1}) = 228 (3.12·10^4), 295 (2.20·10^4) nm. EPR (CH_2Cl_2, 6 K): g = 3.89. IR (ATR) $\tilde{\nu}$ (cm^{-1}) = 3065w, 2985w, 2916m, 2895m, 2863m, 2797m, 1609m, 1595m, 1512s, 1465s, 1419m, 1396s, 1369m, 1252m, 1224m, 1158vs, 1062m, 1015m, 974m, 933w, 913m, 887s, 842m, 796m, 745m, 727s, 697m.

[Co(acac)$_2$(L2)]: L2 (60 mg, 0.16 mmol) was dissolved in 10 mL of CH_2Cl_2. Then $Co(acac)_2$ (42 mg, 0.16 mmol) was added in one portion. The colour of the solution

immediately changed to red. After 2 h of stirring the solvent was removed in vacuo and the crude product was dissolved in 20 mL of *n*-hexane. The solution was filtrated to remove excess Co(acac)$_2$ and dried in vacuo to yield 95 mg (93% 0.15 mmol) of [Co(acac)$_2$(L2)] as a brown-red solid. Crystals suitable for structural characterization by single-crystal X-ray diffraction were grown from a hot saturated *n*-hexane solution. C,H,N analysis (%) for $C_{29}H_{42}CoN_6O_6$: calcd. C 55.32, H 6.72, N 13.35; found C 54.37, H 6.72, N 13.35. UV-vis (CH_2Cl_2): λ_{max} (ε in L·mol^{-1} cm^{-1}) = 229 (3.00·10^4), 290 (2.42·10^4) nm. EPR (CH_2Cl_2, 6 K): g = 3.84. IR (ATR) $\tilde{\nu}$ (cm^{-1}) = 2988w, 2922m, 2854m, 2788m, 1615w, 1593m, 1552s, 1509s, 1480s, 1407vs, 1353m, 1286m, 1235m, 1207m, 1187m, 1154s, 1082w, 1061w, 1024m, 1008m, 970s, 901m, 847m, 815s, 789m, 750m, 715m.

[Co(acac)$_2$(L3)]: L3 (60 mg, 0.14 mmol) was dissolved in 10 mL of CH_2Cl_2. Then Co(acac)$_2$ (36 mg, 0.14 mmol) was added in one portion. The colour of the solution immediately changed to pink-red. After 2 h of stirring the solvent was removed in vacuo and the crude product was dissolved in 20 mL of *n*-hexane. The solution was filtrated to remove excess Co(acac)$_2$ and dried in vacuo to yield 94 mg (98% 0.14 mmol) of [Co(acac)$_2$(L3)] as a pink solid. Crystals suitable for structural characterization by single-crystal X-ray diffraction were grown from a hot saturated *n*-hexane solution. C,H,N analysis (%) for $C_{33}H_{54}CoN_6O_6$: calcd. C 57.46, H 7.89, N 12.18; found C 57.20, H 7.89, N 11.71. UV-vis (CH_2Cl_2): λ_{max} (ε in L·mol^{-1} cm^{-1}) = 234 (2.24·10^4), 291 (2.34·10^4) nm. EPR (CH_2Cl_2, 6 K): g = 3.97. IR (ATR) $\tilde{\nu}$ (cm^{-1}) = 3281m, 2971m, 2932m, 2872w, 1588vs, 1511vs, 1458vs, 1407s, 1381s, 1303m, 1252s, 1212w, 1189m, 1170m, 1147s, 1125m, 1063w, 1011m, 977s, 915m, 891m, 844s, 804w, 787w, 758m, 740w, 717m.

2.2 Single-oxidized complexes

[Co(III)(acac)$_2$(L1)]PF$_6$: [Co(acac)$_2$(L1)] (40 mg, 0.63 mmol) was dissolved in 10 mL dichloromethane. Then ferrocenium-hexafluorophosphate (19 mg, 0.56 mmol) was added and the colour of the solution turned to dark green. The reaction mixture was stirred for 2 h at room temperature. Subsequently the solvent was removed in vacuo and the the crude green product was washed with *n*-hexane (4 x 10 mL) to yield 39 mg (81%, 0.51 mmol) of [Co(III)(acac)$_2$(L1)]PF$_6$ as a dark-green solid. Crystals suitable for structural characterization by single-crystal X-ray diffraction were grown by diffusion of

diethyl ether into a saturated acetonitrile solution. C,H,N analysis (%) for $C_{29}H_{46}CoF_6N_6O_6P$: calcd. C 44.74, H 5.96, N 10.79; found C 45.12, H 5.85, N 10.31. ^1H NMR (200 MHz, CD_2Cl_2): δ = 5.45 (s, 2 H), 4.15 (s, 2 H), 2.86(br. s, 24 H), 2.03 (s, 12 H), 1.61 (s, 6 H) ppm. UV-vis (CH_2Cl_2): λ_{max} (ε in L·mol^{-1} cm^{-1}) = 230 (4.43·10^4), 344 (1.30·10^4) nm. IR (ATR) $\tilde{\nu}$ (cm^{-1}) = 2993w, 2941m, 1614m, 1565m, 1517m, 1468s, 1392s, 1298m, 1238w, 1216m, 1173w, 1143m, 1108w, 1086w, 1064m, 1024m, 983m, 827vs, 802m, 693w.

[Co(III)(acac)$_2$(L2)]PF$_6$: [Co(acac)$_2$(L2)] (40 mg, 0.64 mmol) was dissolved in 10 mL dichloromethane. Then ferrocenium-hexafluorophosphate (19 mg, 0.56 mmol) was added and the colour of the solution turned to dark green. The reaction mixture was stirred for 2 h at room temperature. Subsequently the solvent was removed in vacuo and the the crude green product was washed with *n*-hexane (4 x 10 mL) to yield 43 mg (88%, 0.56 mmol) of [Co(III)(acac)$_2$(L2)]PF$_6$ as a dark-green solid. C,H,N analysis (%) for $C_{29}H_{42}CoF_6N_6O_6P$: calcd. C 44.97, H 5.47, N 10.85; found C 44.34, H 5.15, N 10.55. UV-vis (CH_2Cl_2): λ_{max} (ε in L·mol^{-1} cm^{-1}) = 232 (2.67·10^4), 285 (1.46·10^4), 370 (0.87·10^4), 471 (0.12·10^4) nm. IR (ATR) $\tilde{\nu}$ (cm^{-1}) = 2983w, 2929m, 2880m, 1576m, 1538m, 1513m, 1470m, 1413m, 1371s, 1295m, 1211s, 1156m, 1106w, 1027m, 976s, 916m, 875w, 834vs, 810m.

[Co(III)(acac)$_2$(L3)]PF$_6$: [Co(acac)$_2$(L3)] (30 mg, 0.43 mmol) was dissolved in 10 mL dichloromethane. Then ferrocenium hexafluorophosphate (13 mg, 0.39 mmol) was added and the colour of the solution turned to green. The reaction mixture was stirred for 2 h at room temperature. Subsequently the solvent was removed in vacuo and the the crude green product was washed with *n*-hexane (4 x 10 mL) and diethyl ether (2 x 5 mL) to yield 26 mg (71%, 0.31 mmol) of [Co(III)(acac)$_2$(L3)]PF$_6$ as a green solid. C,H,N analysis (%) for $C_{33}H_{54}CoF_6N_6O_6P$: calcd. C 47.48, H 6.52, N 10.07; found C 46.99, H 6.21, N 9.46. UV-vis (CH_2Cl_2): λ_{max} (ε in L·mol^{-1} cm^{-1}) = 233 (1.76·10^4), 287 (1.30·10^4), 385 (0.23·10^4).

2.3 Twofold-oxidized complexes

[Co(III)(acac)$_2$(L1)](PF$_6$)$_2$: [Co(acac)$_2$(L1)] (40 mg, 0.63 mmol) was dissolved in 10 mL dichloromethane. Then ferrocenium hexafluorophosphate (40 mg, 1,20 mmol) was added and the colour of the solution changed to green and after 5 min of stirring to

deep-purple. The reaction mixture was stirred for another 2 h at room temperature. Subsequently the solvent was removed in vacuo and the the crude violet product was washed with *n*-hexane (4 x 10 mL) and diethyl ether (2 x 10 mL) to yield 50 mg (85%, 0.54 mmol) of [Co(III)(acac)$_2$(L1)](PF$_6$)$_2$ as a purple solid. C,H,N analysis (%) for $C_{29}H_{46}CoF_{12}N_6O_6P_2$: calcd. C 37.71, H 5.02, N 9.10; found C 37.39, H 5.04, N 8.88. UV-vis (CH$_2$Cl$_2$): λ_{max} (ε in L·mol^{-1} cm^{-1}) = 230 (4.45·10^4), 294 (1.68·10^4), 364 (1.31·10^4), 554 (0.31·10^4) nm. EPR (CH$_2$Cl$_2$, 6 K): g = 2.00, (CH$_2$Cl$_2$, 293 K): g = 1.997132. IR (ATR) $\tilde{\nu}$ (cm^{-1}) = 2990w, 2943m, 1611m, 1565m, 1516s, 1466s, 1391s, 1300m, 1238m, 1217m, 1172w, 1155w, 1143m, 1107w, 1085w, 1064m, 1025m, 981m, 892w, 877w, 829vs, 802m, 741w, 692w.

[Co(III)(acac)$_2$(L1)](SbF$_6$)$_2$: [Co(acac)$_2$(L1)] (30 mg, 0.47 mmol) was dissolved in 10 mL dichloromethane. Then nitrosyl hexafluoroantimonate (24 mg, 0.90 mmol) was added and the colour of the solution changed to green and after 15 min of stirring to deep-purple. The reaction mixture was stirred for another 2 h at room temperature and was evacuated periodically. Subsequently the solvent was removed in vacuo and the the crude red-purple product was washed with *n*-hexane (4 x 10 mL) and diethyl ether (2 x 10 mL) to yield 38 mg (79%, 0.37 mmol) of [Co(III)(acac)$_2$(L1)](SbF$_6$)$_2$ as a red-violet solid. Crystals suitable for structural characterization by single-crystal X-ray diffraction were grown by diffusion of diethyl ether into a saturated tetrahydrofurane solution. C,H,N analysis (%) for $C_{29}H_{46}CoF_{12}N_6O_6Sb_2$: calcd. C 31.52, H 4.20, N 7.60; found C 30.17, H 4.30, N 8.00. UV-vis (CH$_2$Cl$_2$): λ_{max} (ε in L·mol^{-1} cm^{-1}) = 230 (4.17·10^4), 281 (2.01·10^4), 364 (0.70·10^4) nm. IR (ATR) $\tilde{\nu}$ (cm^{-1}) = 3371m, 2992w, 2940m, 1628m, 1551s, 1518s, 1494m, 1467s,1408vs, 1304m, 1276s, 1257w, 1217s, 1182s, 1166m, 1143w, 1106w, 1064m, 1025m, 978m, 933w, 888m, 864w, 833w, 803m, 784m, 735m.

[Co(III)(acac)$_2$(L2)](PF$_6$)$_2$: [Co(acac)$_2$(L2)] (30 mg, 0.48 mmol) was dissolved in 10 mL dichloromethane. Then ferrocenium hexafluorophosphate (30 mg, 0.91 mmol) was added and the colour of the solution changed to green and after 15 min of stirring to red-purple. The reaction mixture was stirred for another 2 h at room temperature. Subsequently the solvent was removed in vacuo and the the crude red-purple product was washed with *n*-hexane (4 x 10 mL) and diethyl ether (2 x 10 mL) to yield 31 mg (84%, 0.40 mmol) of [Co(III)(acac)$_2$(L2)](PF$_6$)$_2$ as a red-violet solid. C,H,N analysis (%) for $C_{29}H_{46}CoF_{12}N_6O_6P_2$: calcd. C 37.88, H 4.60, N 9.14; found C 36.90 H 5.07, N 8.44.

UV-vis (CH_2Cl_2): λ_{max} (ε in L·mol^{-1} cm^{-1}) = 231 ($3.09\cdot10^4$), 293 ($1.54\cdot10^4$), 334 ($1.31\cdot10^4$), 503 ($0.28\cdot10^4$) nm. EPR (CH_2Cl_2, 293 K): g = 2.00. IR (ATR) $\tilde{\nu}$ (cm^{-1}) = 3622w, 3553w, 2985w, 1626s, 1573m, 1525m, 1483w, 1423s, 1387w, 1371m, 1301m, 1271w, 1253m, 1219s, 1193m, 1154m, 1139vs, 1101m, 1087m, 1022m, 985m, 974w, 949w, 918w, 894w, 831w, 818m, 808s, 789m, 768w, 744m, 705w.

[Co(II)(acac)$_2$(L3)](PF$_6$)$_2$: [Co(acac)$_2$(L3)] (40 mg, 0.58 mmol) was dissolved in 10 mL dichloromethane. Then ferrocenium hexafluorophosphate (36 mg, 1.10 mmol) was added and the colour of the solution changed to green and after 5 min of stirring to yellow-brown. The reaction mixture was stirred for another 2 h at room temperature. Subsequently the solvent was removed in vacuo and the the crude dark-yellow product was washed with *n*-hexane (4 x 10 mL) and diethyl ether (2 x 10 mL) to yield 48 mg (84%, 0.49 mmol) of [Co(II)(acac)$_2$(L3)](PF$_6$)$_2$ as a dark-yellow solid. Crystals suitable for structural characterization by single-crystal X-ray diffraction were grown by diffusion of diethyl ether into a saturated dichloromethane solution. C,H,N analysis (%) for $C_{33}H_{54}CoF_{12}N_6O_6P_2$: calcd. C 40.46, H 5.56, N 8.58; found C 40.97 H 5.54, N 8.56. UV-vis (CH_2Cl_2): λ_{max} (ε in L·mol^{-1} cm^{-1}) = 228 ($2.06\cdot10^4$), 257 ($1.63\cdot10^4$), 295 ($1.68\cdot10^4$), 450 ($0.26\cdot10^4$) nm. EPR (CH_2Cl_2, 6 K): g = 2.00. IR (ATR) $\tilde{\nu}$ (cm^{-1}) = 3333m, 2989w, 2944w, 1643s, 1549s, 1466w, 1420s, 1386m, 1349w, 1325w, 1254s, 1201s, 1147s, 1099m, 1031w, 979w, 831s, 847m, 814vs, 801w, 768w, 742m, 667s.

3. Analytical data

3.1 NMR spectra

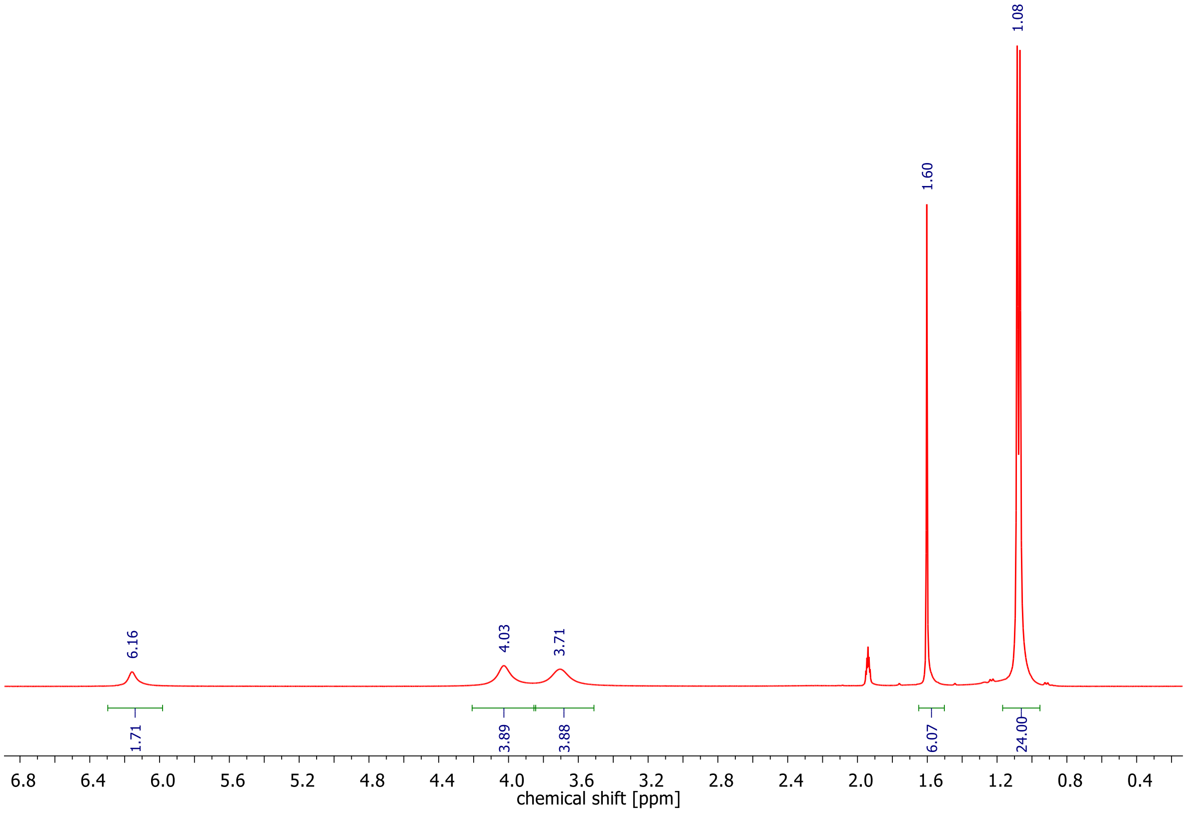

Figure S1: ^{1}H NMR spectrum (400 MHz, CD_3CN) of L3.

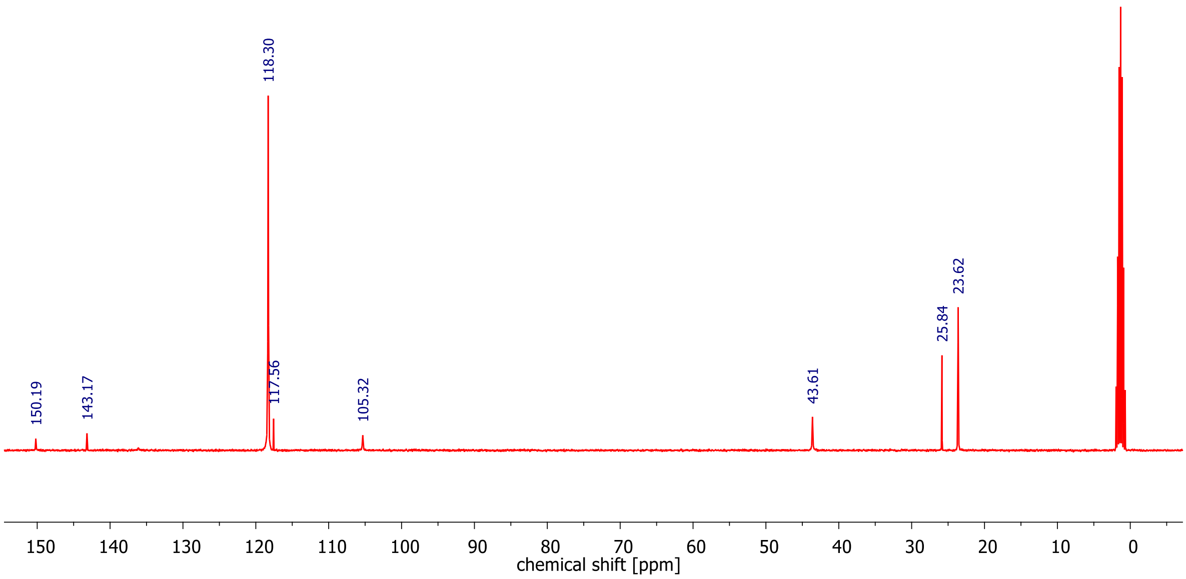

Figure S2: ^{13}C NMR spectrum (400 MHz, CD_3CN) of L3.

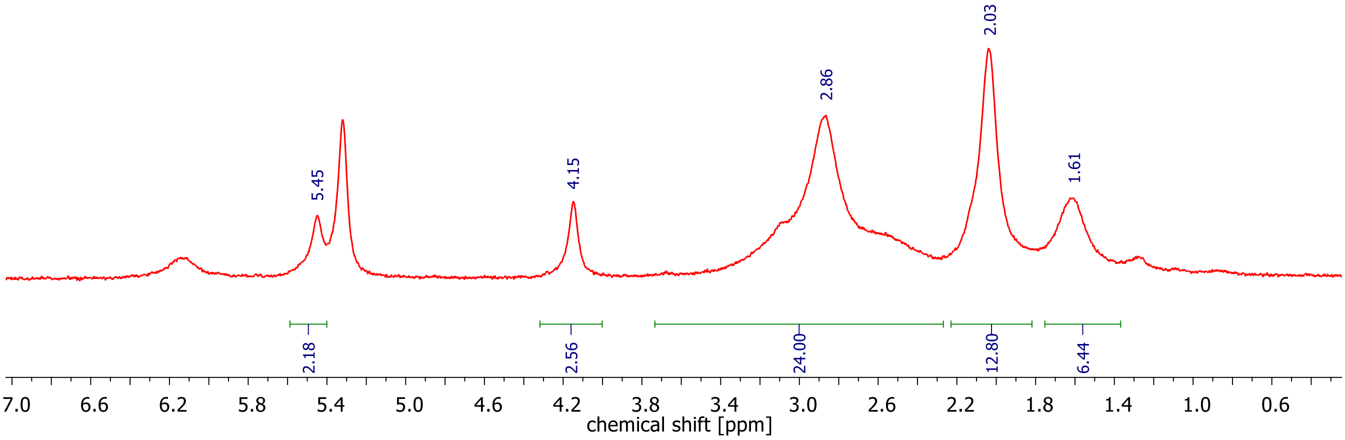

Figure S3: ^{1}H NMR spectrum (200 MHz, CD_2Cl_2) of [L1Co(III)(acac)$_2$]PF_6.

3.2 UV-vis spectra

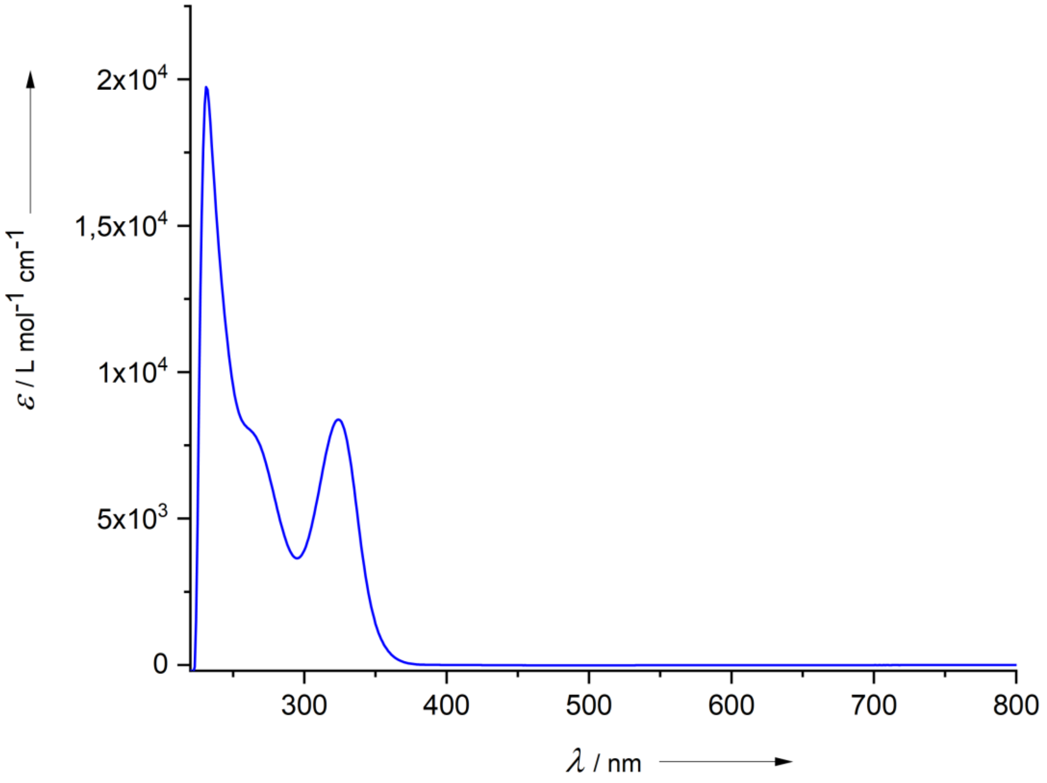

Figure S4: UV-vis spectrum (CH_2Cl_2) of L3**.**

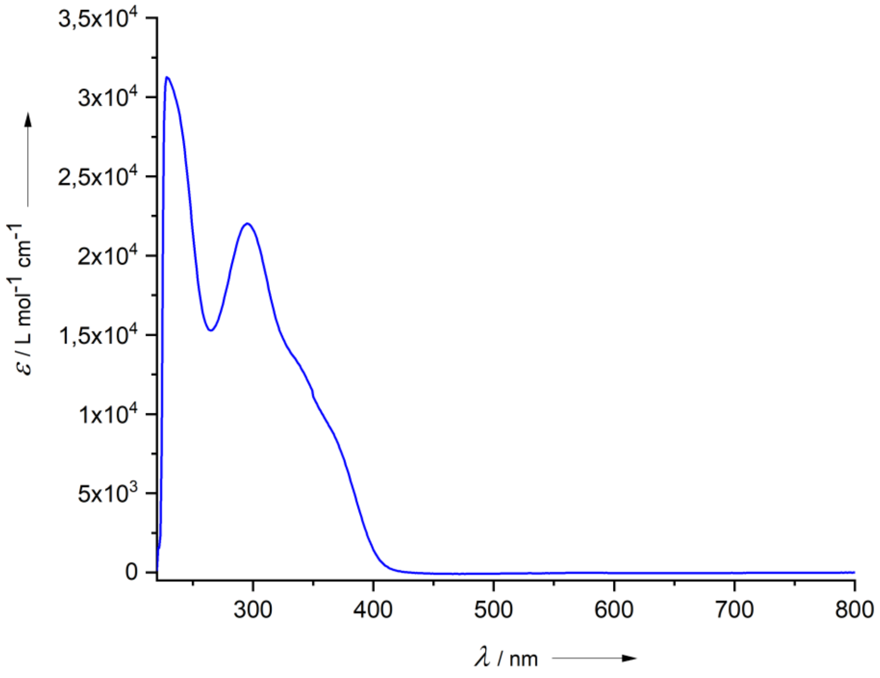

Figure S5: UV-vis spectrum (CH_2Cl_2) of [Co(acac)$_2$(L1)].

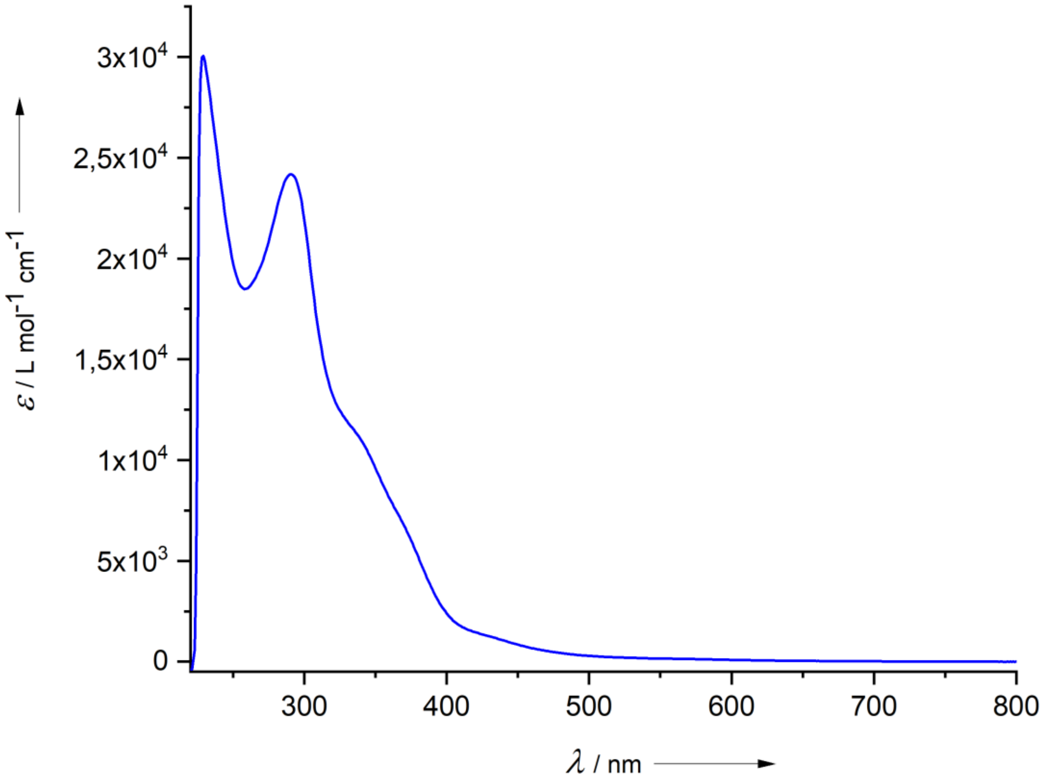

Figure S6: UV-vis spectrum (CH_2Cl_2) of [Co(acac)$_2$(L2)].

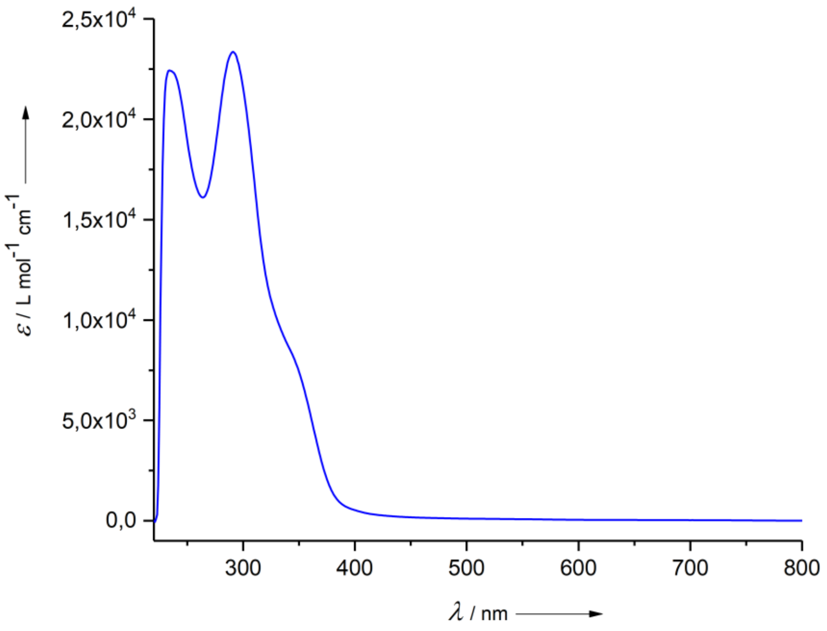

Figure S7: UV-Vis spectrum (CH_2Cl_2) of [Co(acac)$_2$(L3)].

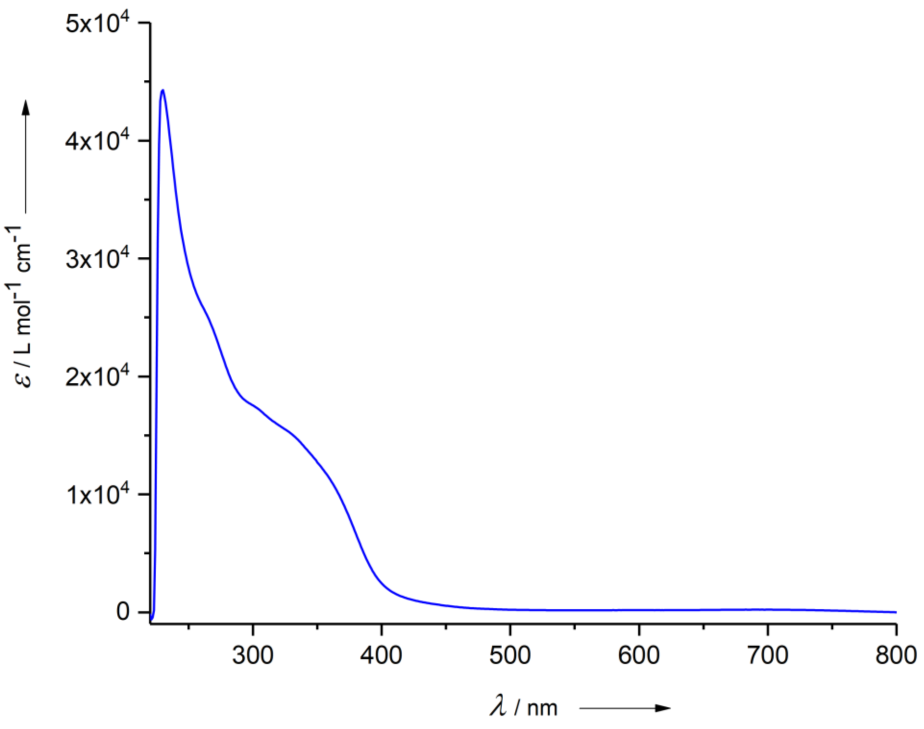

Figure S8: UV-vis spectrum (CH_2Cl_2) of [Co(III)(acac)$_2$(L1)]PF_6.

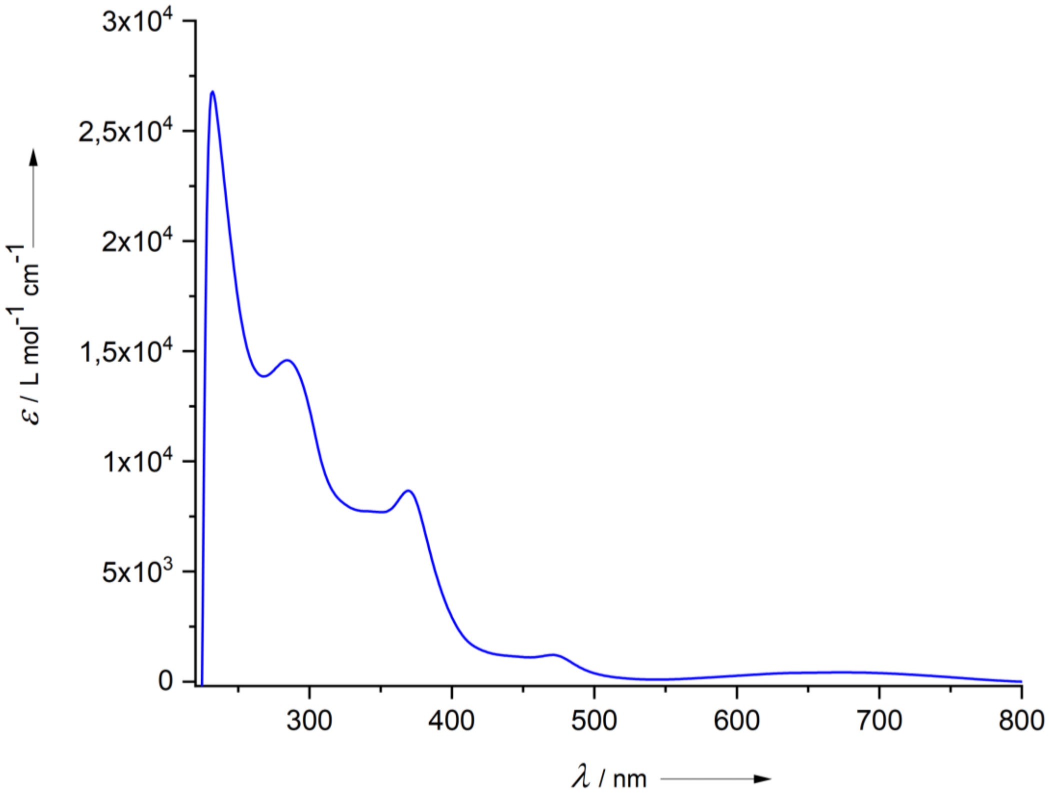

Figure S9: UV-vis spectrum (CH_2Cl_2) of $[Co(III)(acac)_2(L2)]PF_6$.

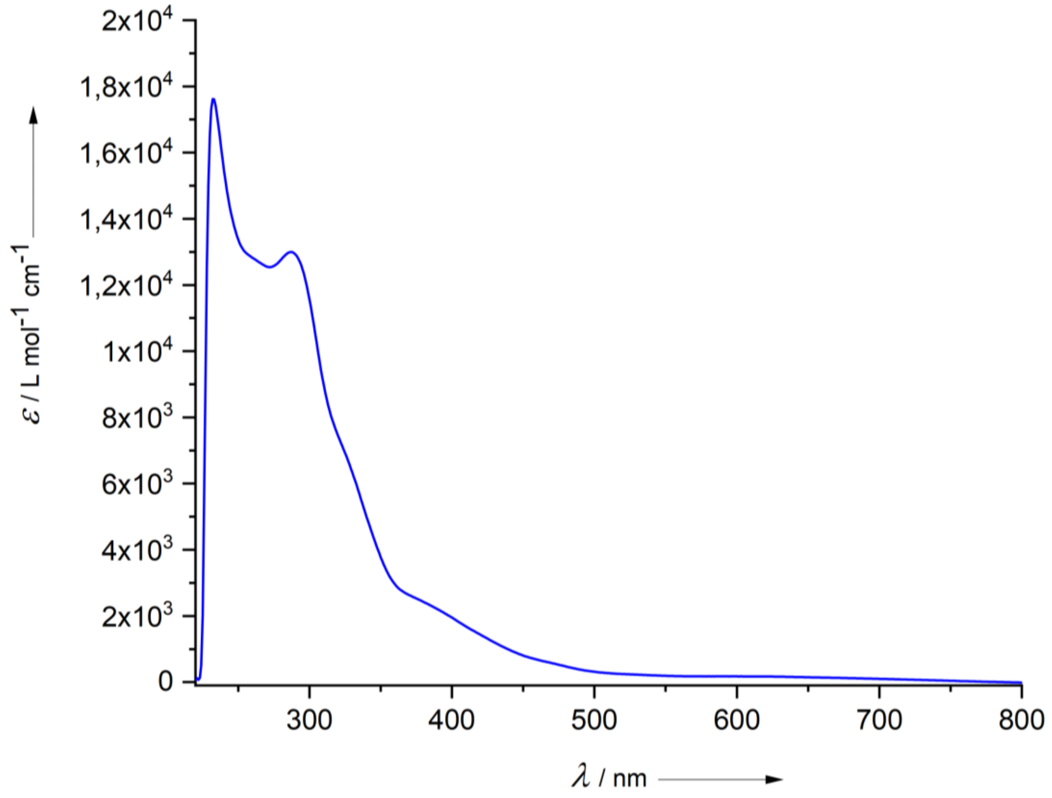

Figure S10: UV-vis spectrum (CH_2Cl_2) of $[Co(III)(acac)_2(L3)]PF_6$.

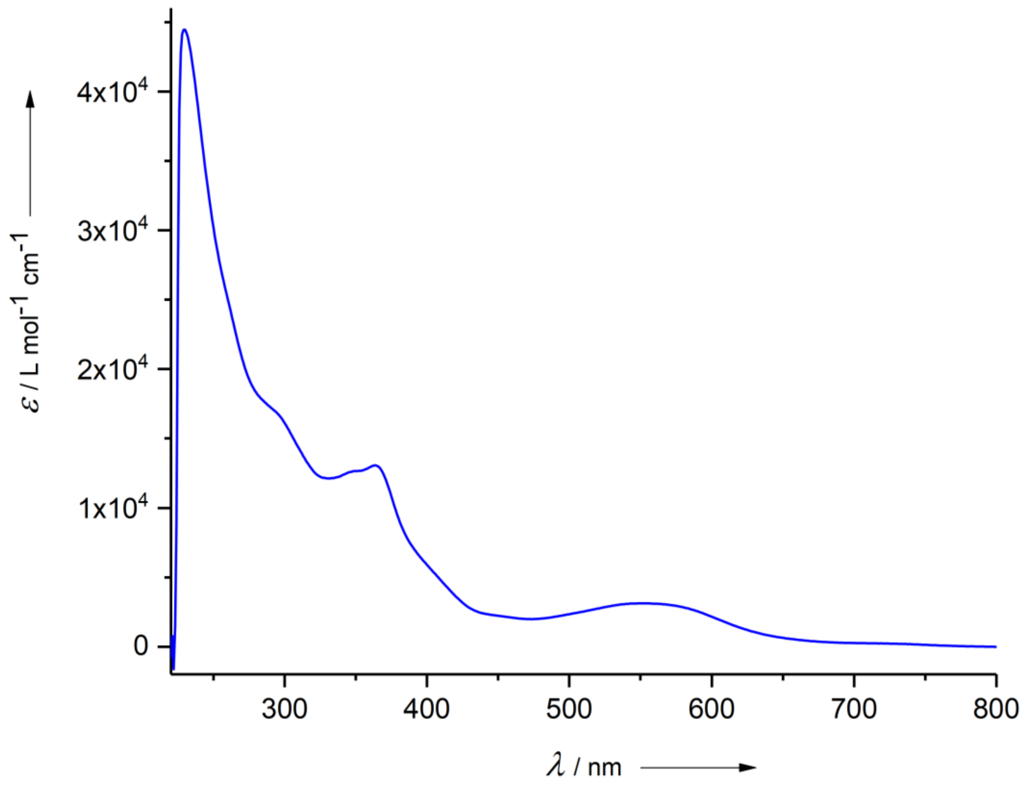

Figure S11: UV-vis spectrum (CH_2Cl_2) of [Co(III)(acac)$_2$(L1)](PF_6)$_2$.

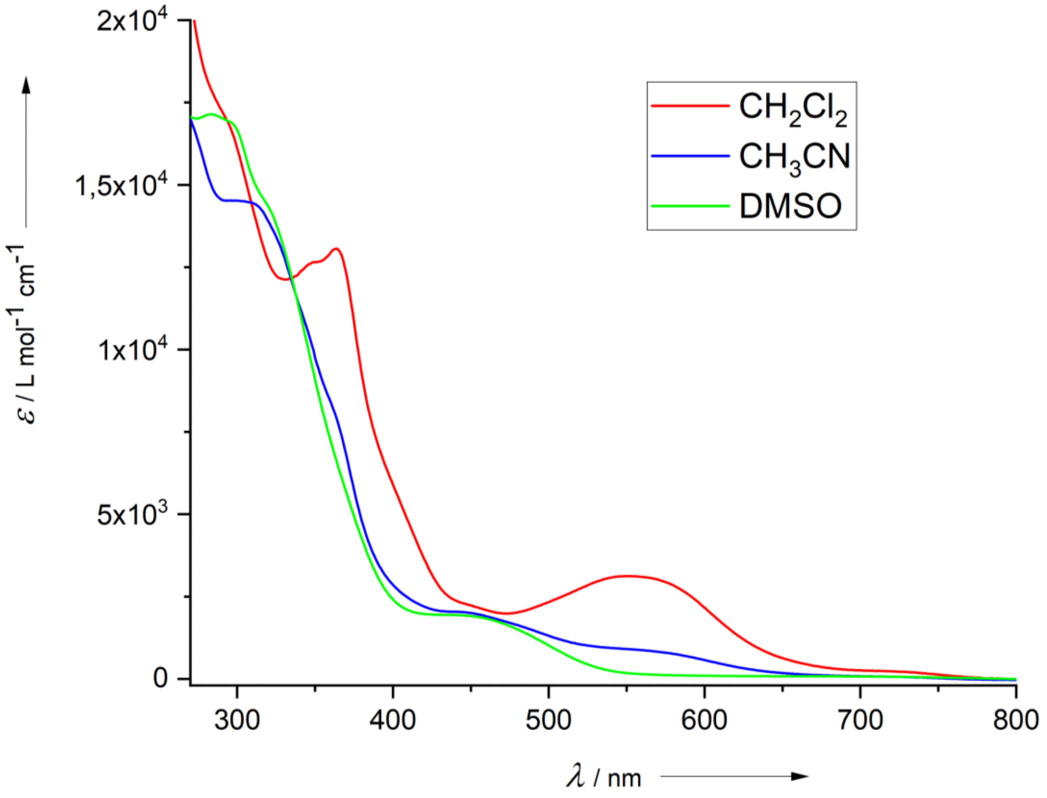

Figure S12: UV-vis spectra CH_2Cl_2, CH_3CN and DMSO of [Co(III)(acac)$_2$(L1)](PF_6)$_2$.

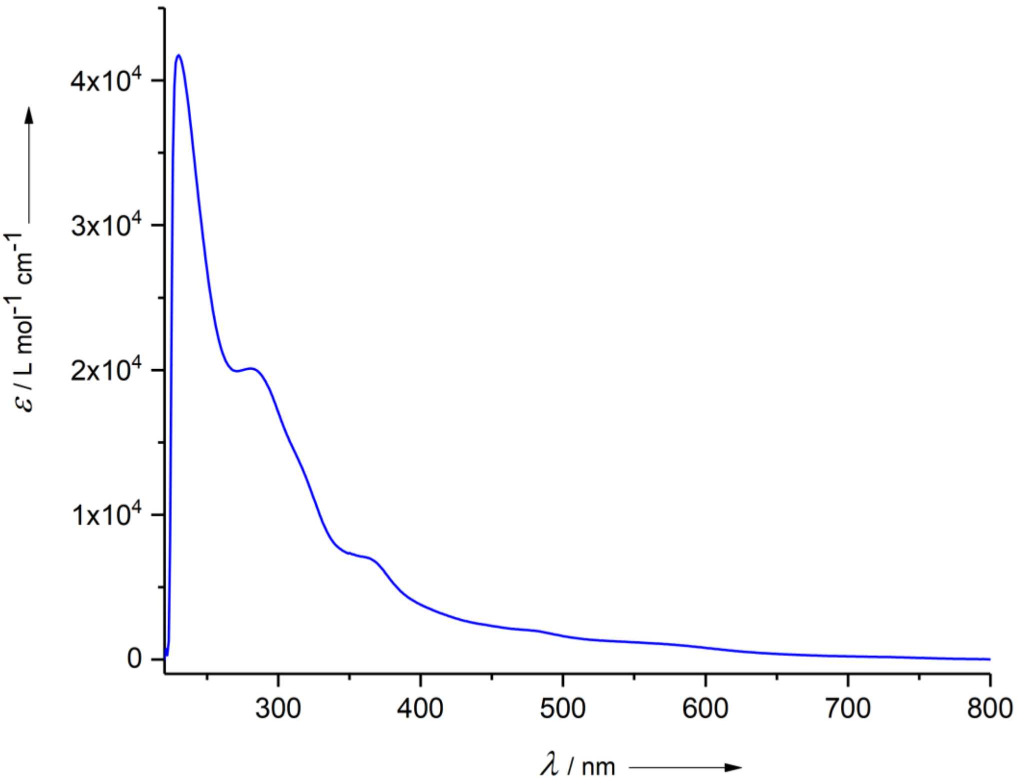

Figure S13: UV-vis spectrum (CH_2Cl_2) of [Co(III)(acac)$_2$(L1)](SbF_6)$_2$.

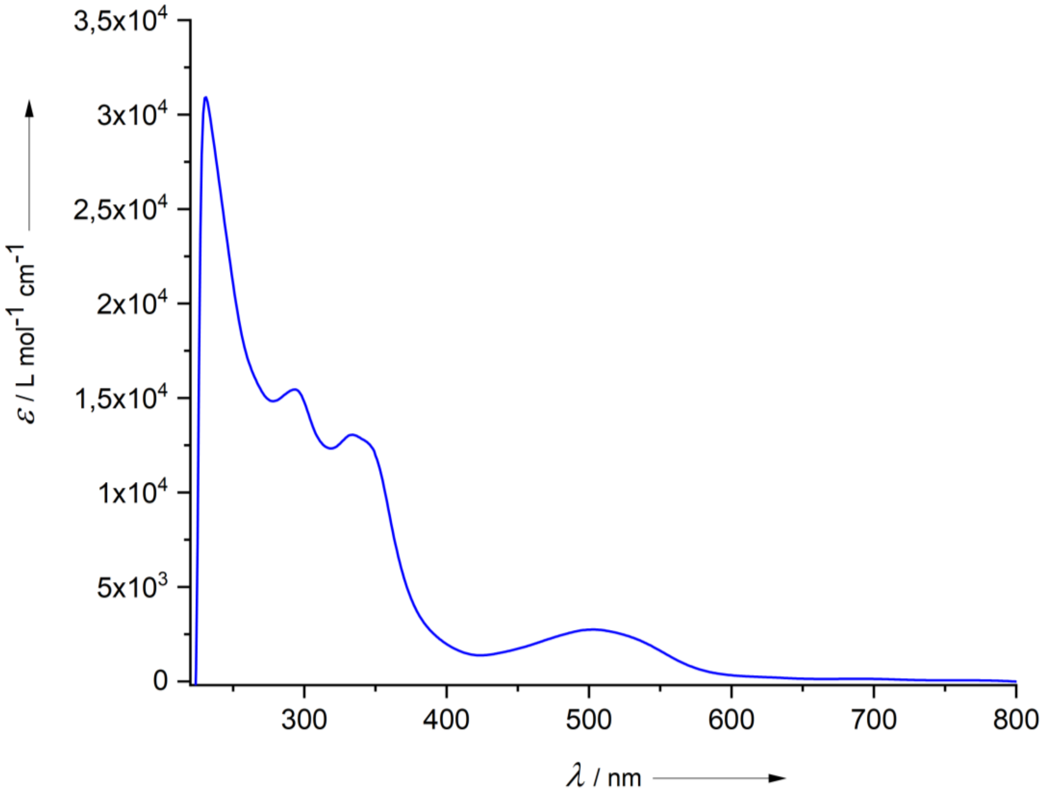

Figure S14: UV-vis spectrum (CH_2Cl_2) of [Co(III)(acac)$_2$(L2)](PF_6)$_2$.

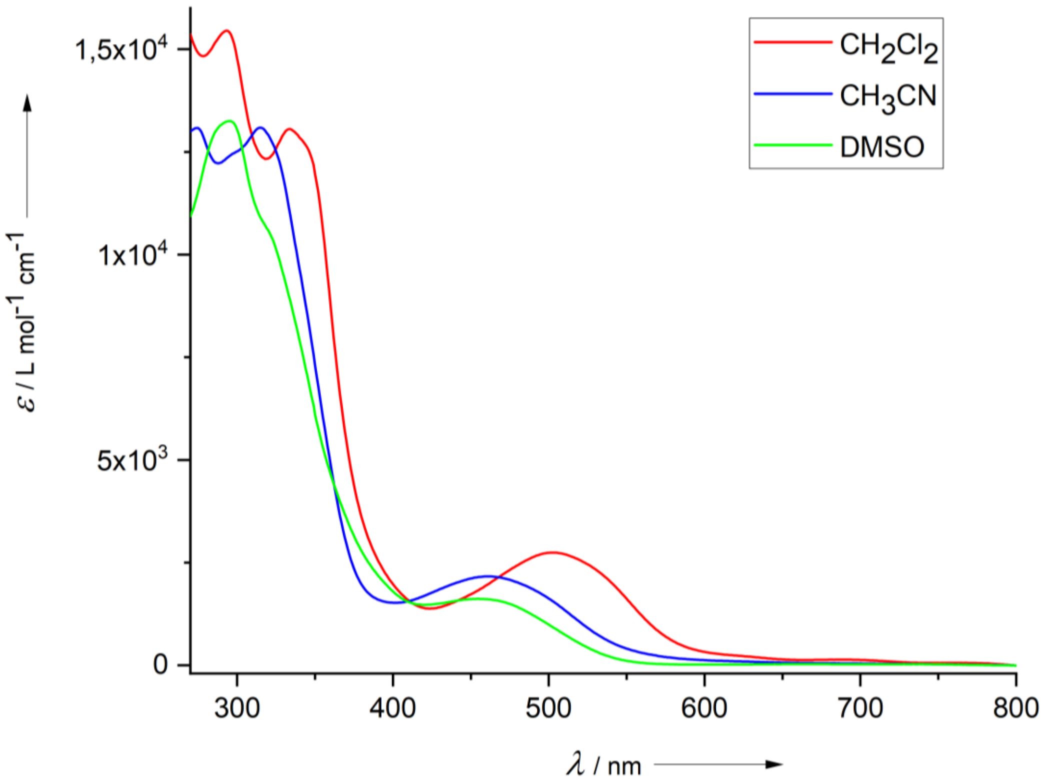

Figure S15: UV-vis spectra CH_2Cl_2, CH_3CN and DMSO of [Co(III)(acac)$_2$(L2)](PF_6)$_2$.

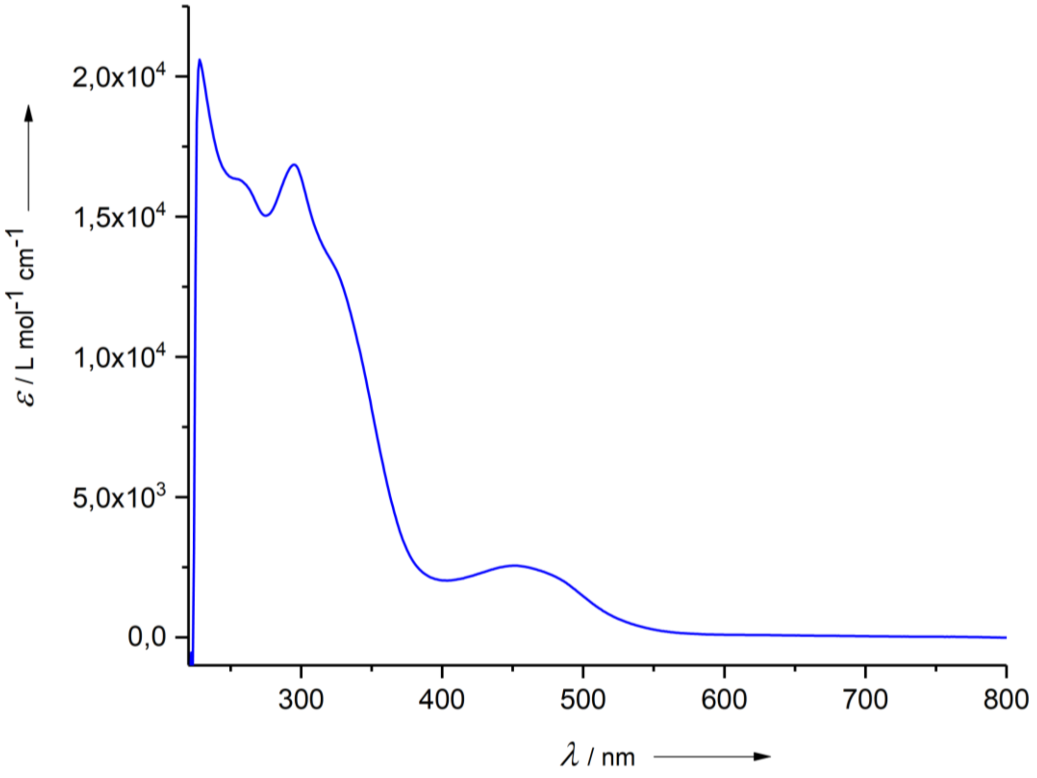

Figure S16: UV-vis spectrum (CH_2Cl_2) of [Co(II)(acac)$_2$(L3)](PF_6)$_2$.

3.3 Cyclic voltammetry (CV) measurements

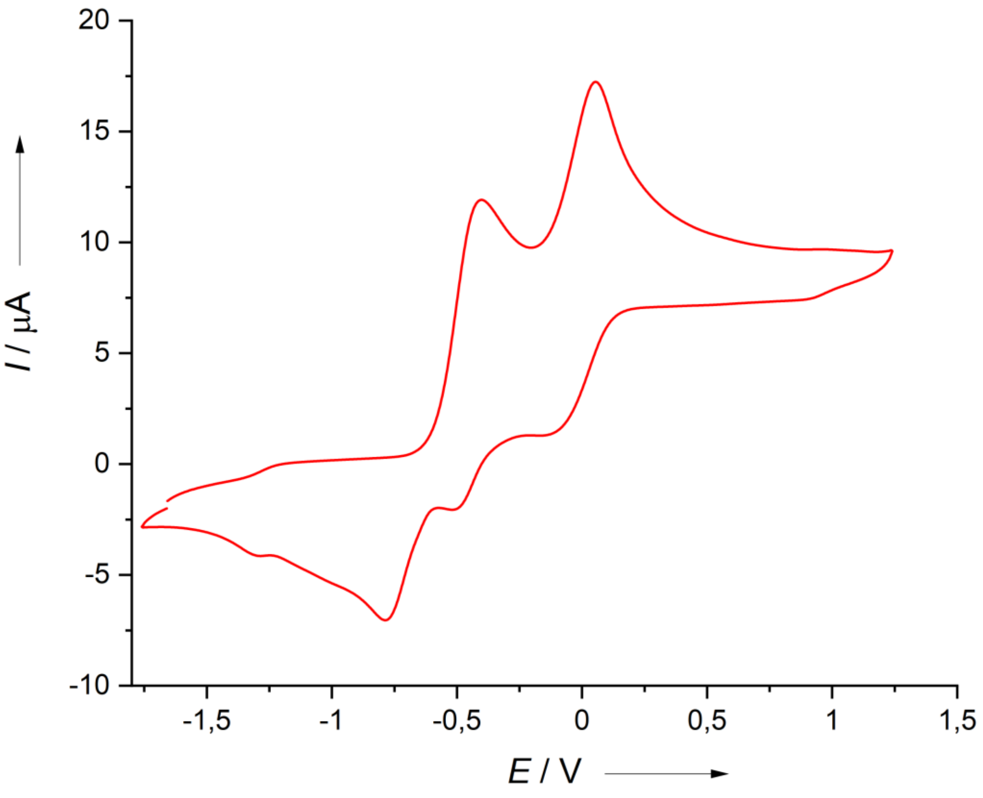

E_{red} (1) = -0.79 V E_{ox} (1) = -0.41 V

E_{red} (2) = -0.51 V E_{ox} (2) = 0.05 V

E_{red} (3) = -0.11 V

Figure S17: CV curve of compound L3 in CH_2Cl_2 (Ag/AgCl reference electrode, 0.1 M $N(nBu)_4(PF_6)$ as supporting electrolyte, scan rate 30 mV s^{-1}). Potentials given vs. the Fc^+/Fc redox couple.

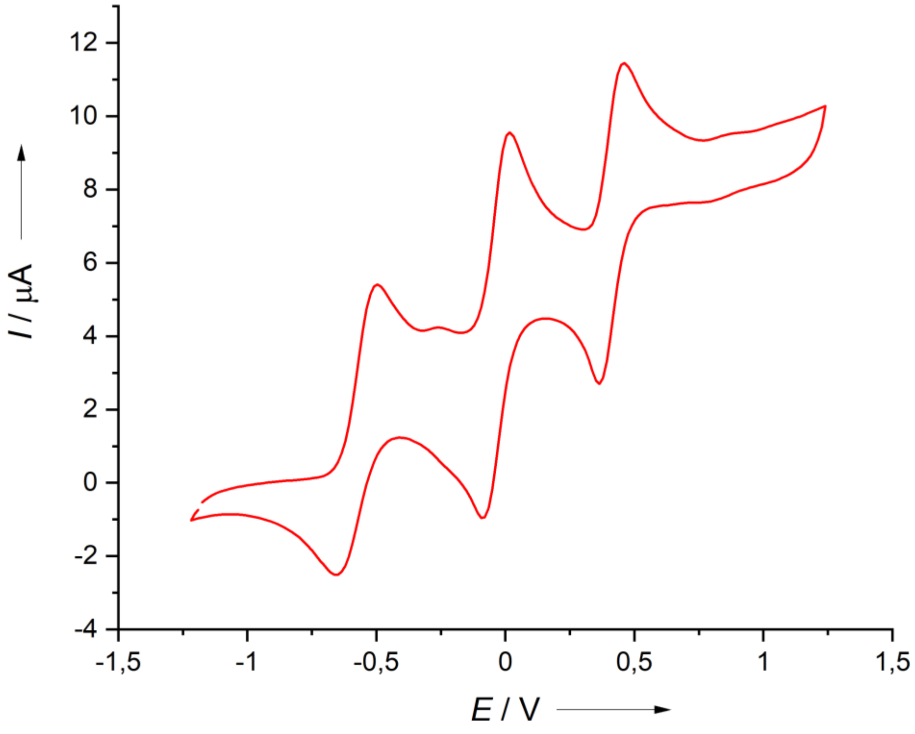

$E_{1/2}$ (1) = -0.56 V

$E_{1/2}$ (2) = -0.03 V

$E_{1/2}$ (3) = 0.41 V

Figure S18: CV curve of compound [Co(acac)$_2$(L1)] in CH_2Cl_2 (Ag/AgCl reference electrode, 0.1 M N(*n*Bu)$_4$(PF$_6$) as supporting electrolyte, scan rate 20 mV s^{-1}). Potentials given vs. the Fc^+/Fc redox couple.

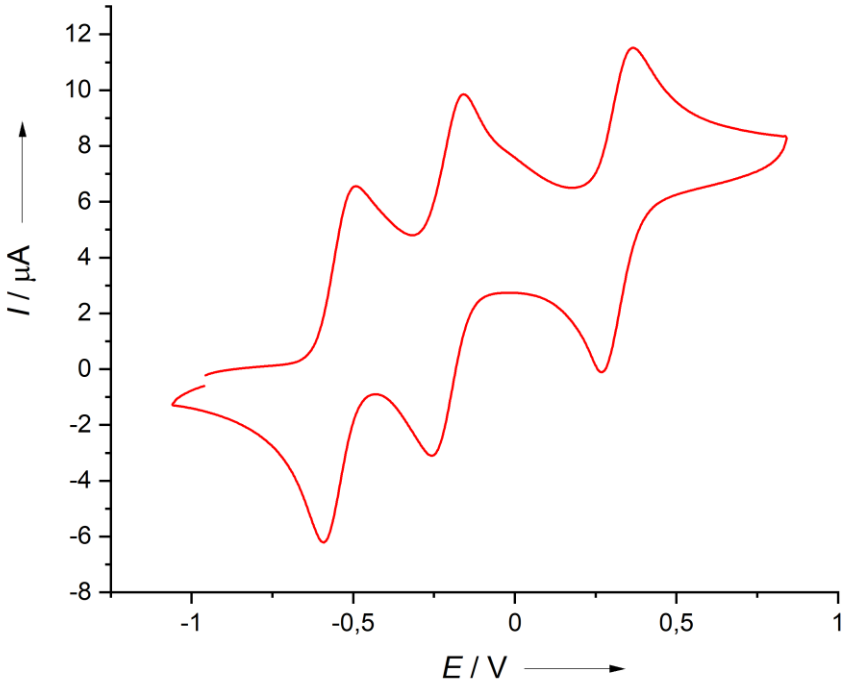

$E_{1/2}$ (1) = -0.54 V

$E_{1/2}$ (2) = -0.21 V

$E_{1/2}$ (3) = 0.32 V

Figure S19: CV curve of compound [Co(acac)$_2$(L2)] in CH_2Cl_2 (Ag/AgCl reference electrode, 0.1 M N(*n*Bu)$_4$(PF$_6$) as supporting electrolyte, scan rate 30 mV s^{-1}). Potentials given vs. the Fc^+/Fc redox couple.

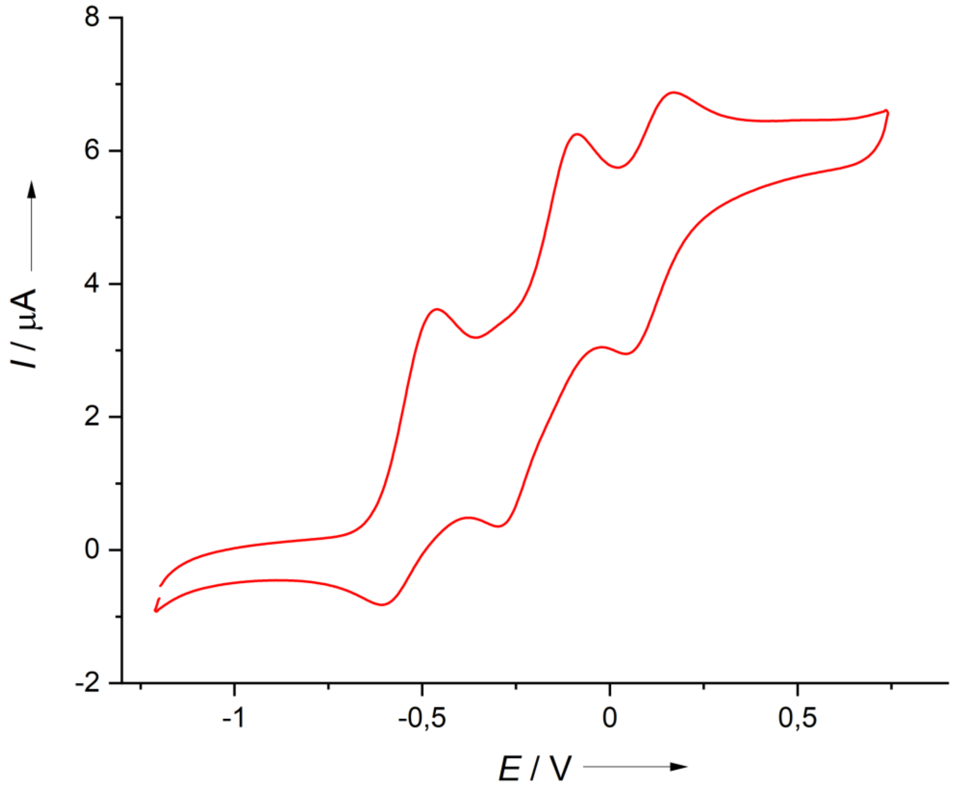

$E_{1/2}$ (1) = -0.53 V

$E_{1/2}$ (2) = -0.19 V

$E_{1/2}$ (3) = 0.11 V

Figure S20: CV curve of compound [Co(acac)$_2$(L3)] in CH_2Cl_2 (Ag/AgCl reference electrode, 0.1 M N(*n*Bu)$_4$(PF$_6$) as supporting electrolyte, scan rate 20 mV s^{-1}). Potentials given vs. the Fc^+/Fc redox couple.

3.4 EPR spectroscopy

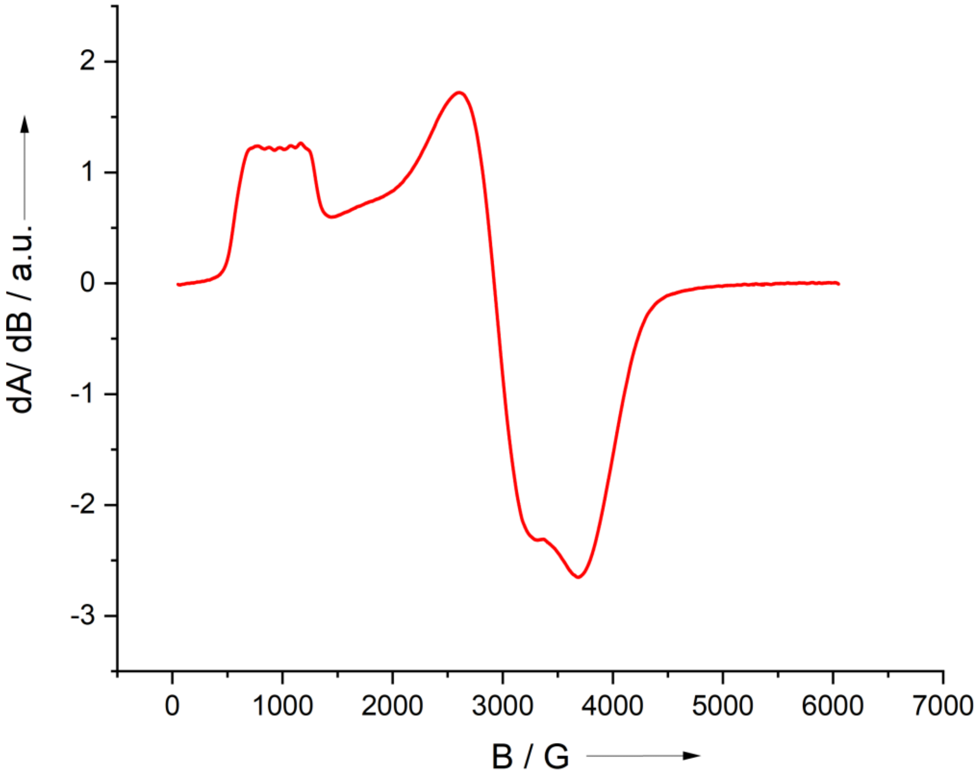

$g_{\perp}$= 7.10, g_{II} = 2.19, g = 3.82

Figure S21: EPR spectrum of [Co(acac)$_2$(L1)] in CH_2Cl_2 at 6 K.

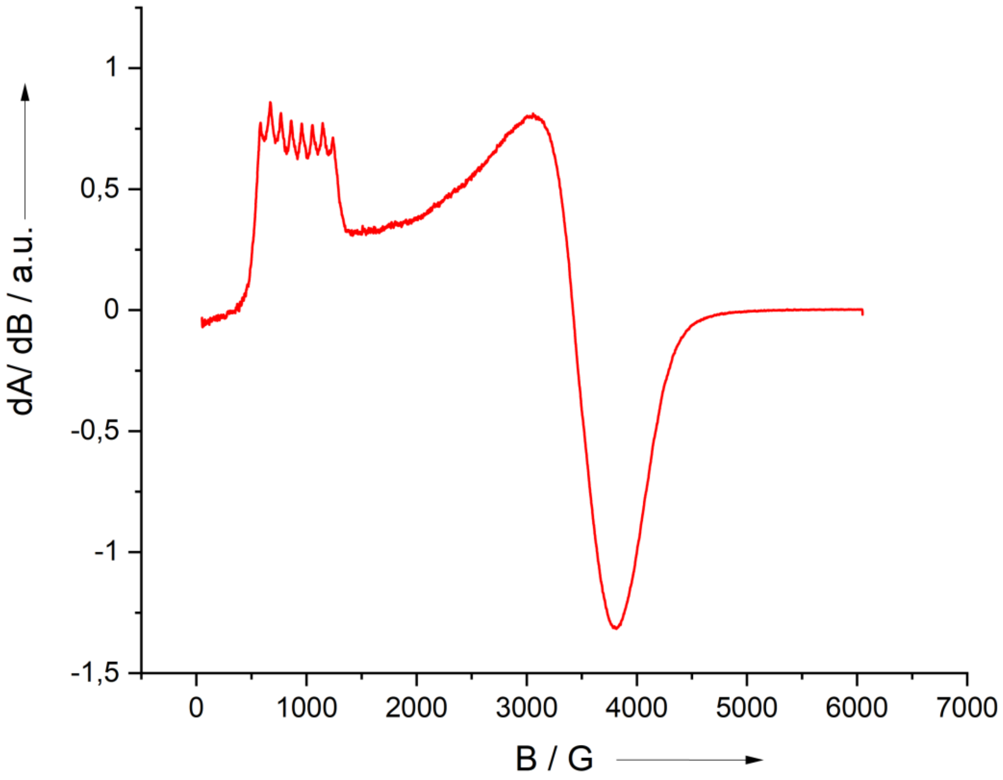

$g_{\perp}$= 7.53, g_{II} = 2.00, g = 3.84

Figure S22: EPR spectrum of [Co(acac)$_2$(L2)] in CH_2Cl_2 at 6 K.

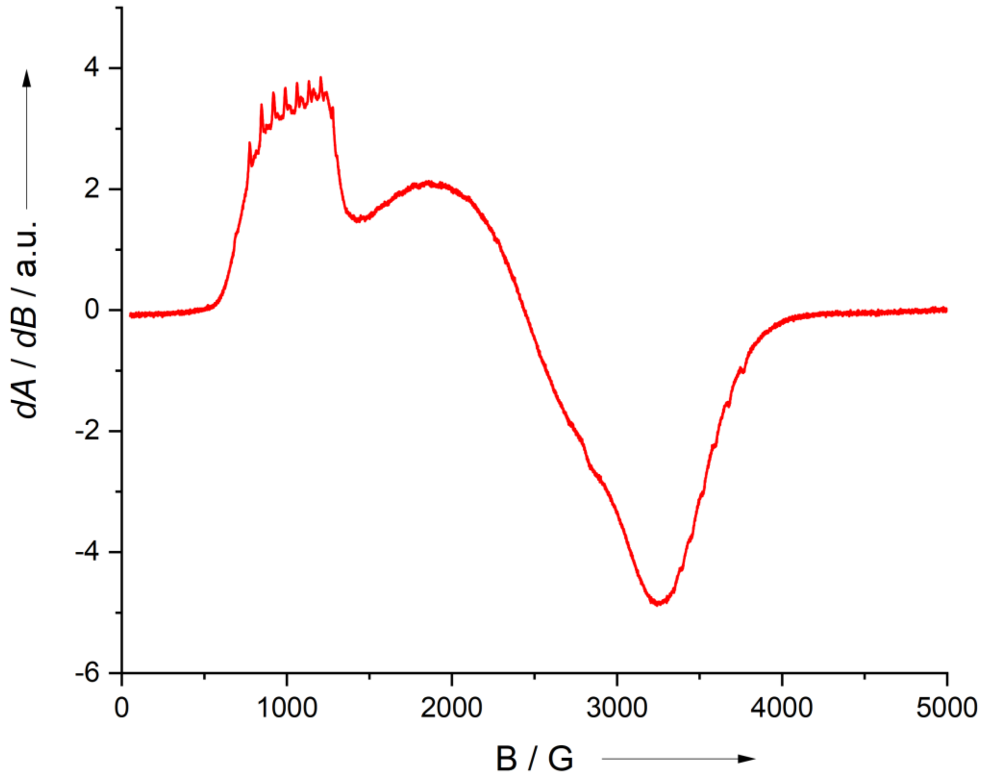

$g_{\perp}$ = 6.66, g_{II} = 2.68, g = 3.97

Figure S23: EPR spectrum of [Co(acac)$_2$(L3)] in CH_2Cl_2 at 6 K.

a)

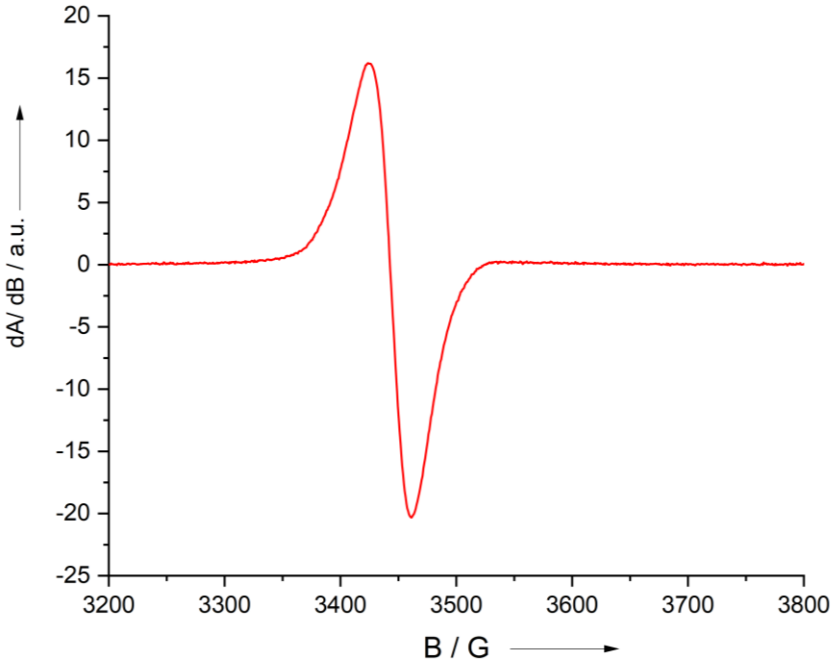

b)

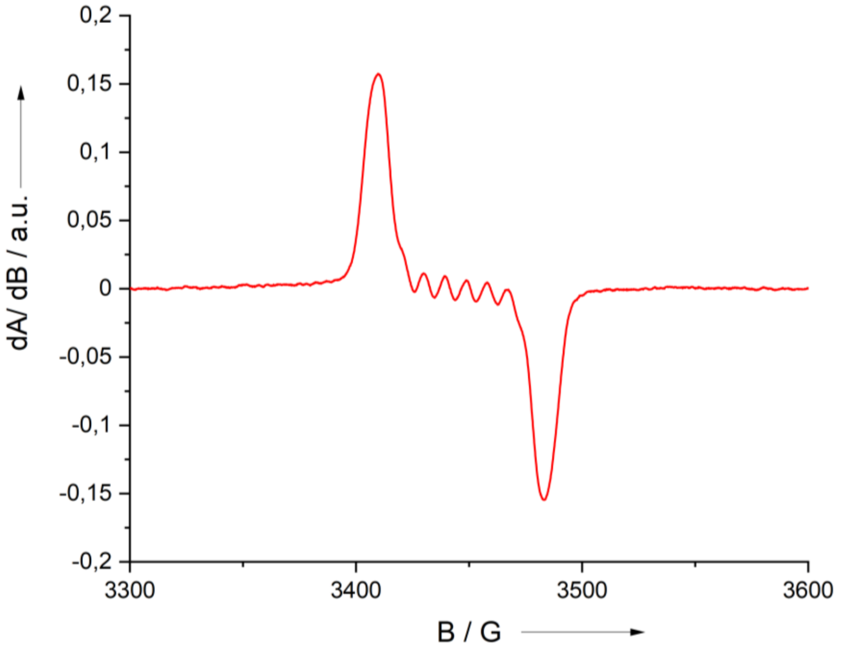

g = 2.00

Figure S24: EPR spectrum (CH_2Cl_2) of [Co(III)(acac)$_2$(L1)](PF_6)$_2$ at 6 K (a) and 293 K (b).

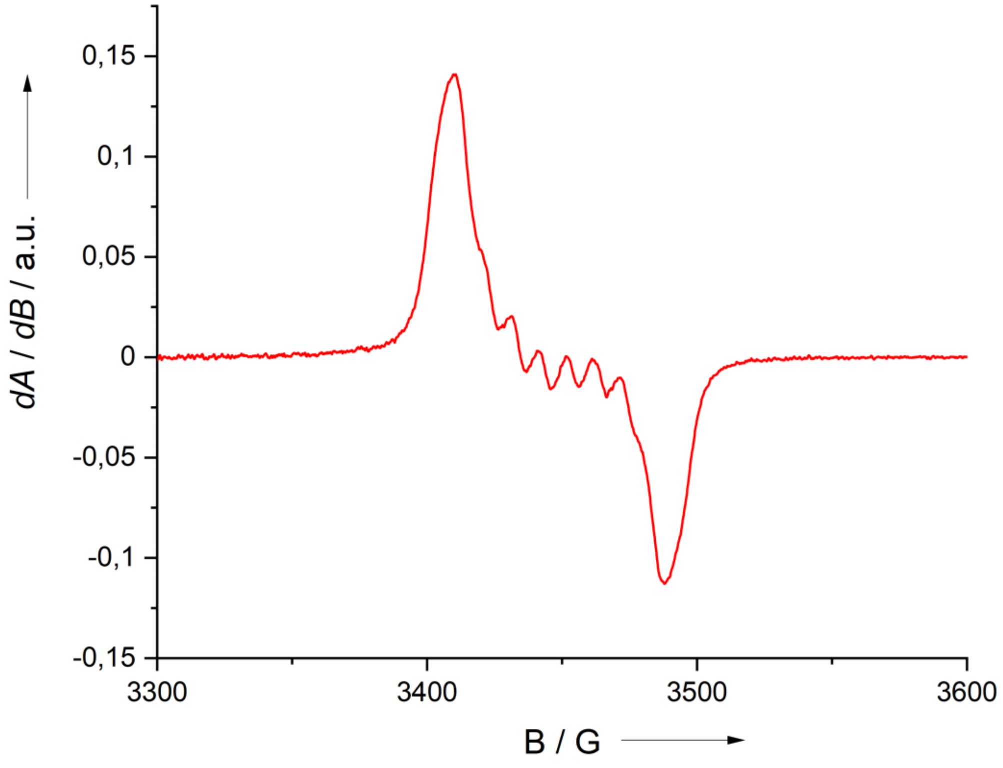

g = 2.00

Figure S25: EPR spectrum (CH_2Cl_2) of $[Co(III)(acac)_2(L2)](PF_6)_2$ at 293 K.

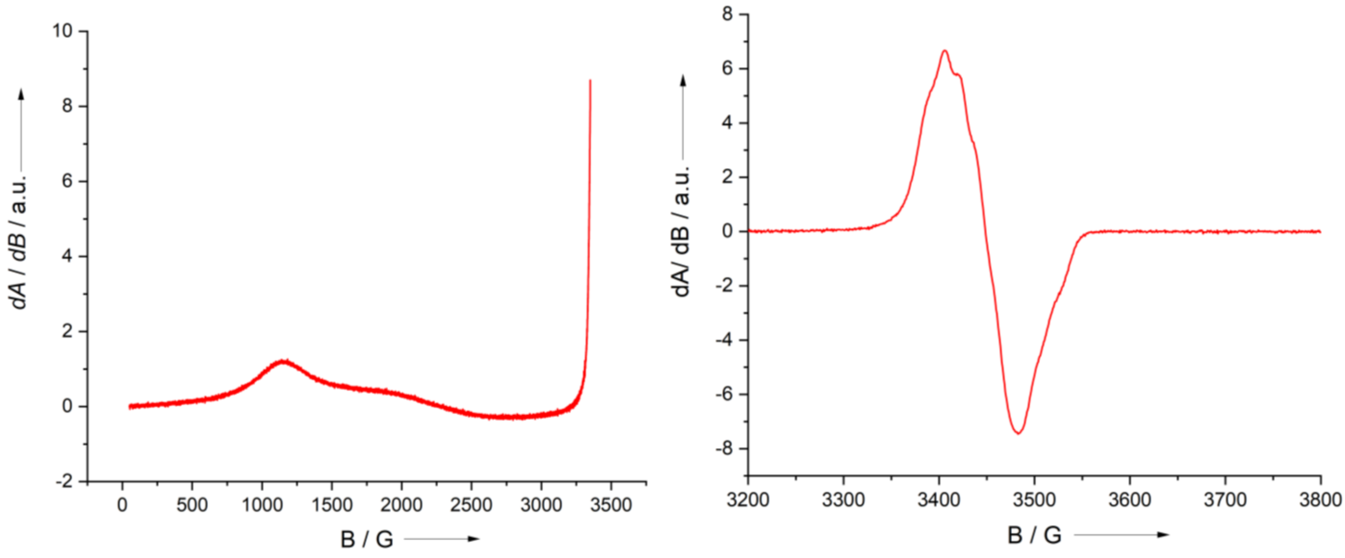

g = 2.00

Figure S26: EPR spectrum (CH_2Cl_2) of $[Co(II)(acac)_2(L3)](PF_6)_2$ at 6 K.

3.5 SQUID measurements

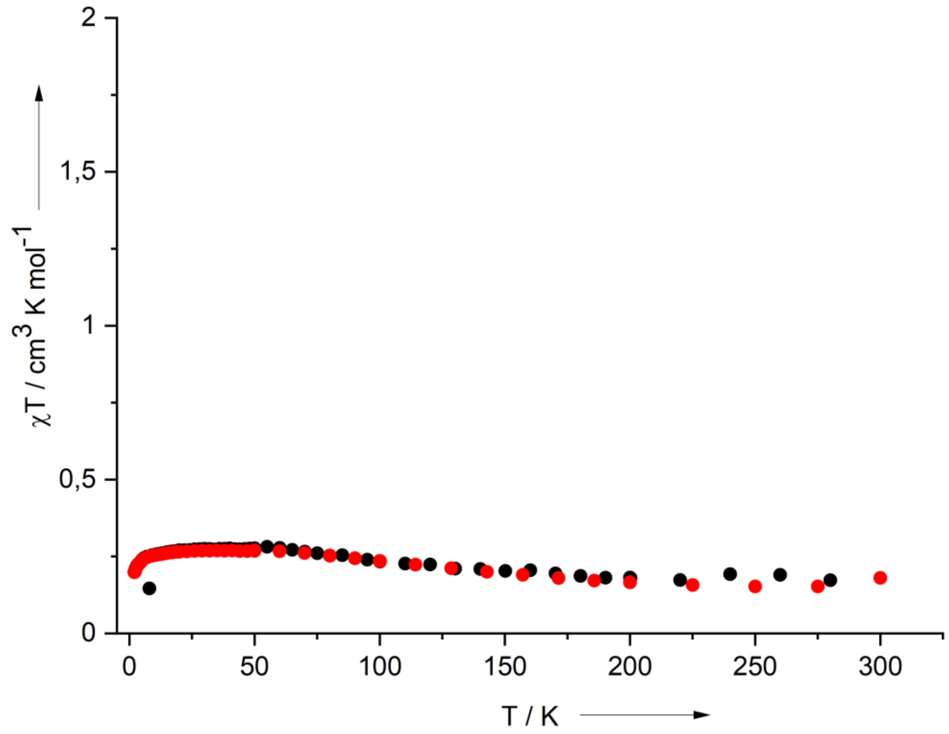

Figure S27: SQUID curves of $[Co(III)(acac)_2(L1)](PF_6)_2$ at 500 Oe (black) and 1000 Oe (red).

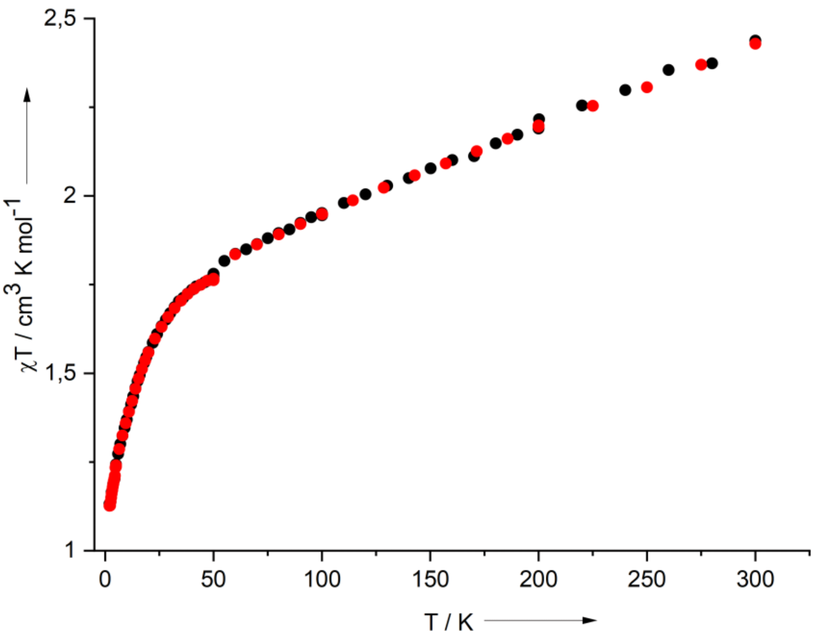

Figure S28: SQUID curves of [Co(II)(acac)$_2$(L3)](PF$_6$)$_2$ at 500 Oe (black) and 1000 Oe (red).

4 Crystallographic data

4.1 Details of the structural characterizations.

Suitable crystals for single-crystal structure determination were taken directly from the mother liquor, immersed in perfluorinated polyether oil and fixed on a cryo loop. Full shells of intensity data for all compounds were collected at low temperature with a Nonius Kappa CCD diffractometer (Mo-K$_α$ radiation, sealed X-ray tube, graphite monochromator, compound L3) an Bruker D8 Venture, dual source (Mo- or Cu-*K*$_α$ radiation, microfocus X-ray tube, Photon III detector, compounds [Co(acac)$_2$(L1)], [Co(acac)$_2$(L2)], [Co(acac)$_2$(L3)], [Co(acac)$_2$(L1)]PF$_6$, [Co(acac)$_2$(L1)](SbF$_6$)$_2$, [Co(acac)$_2$(L3)](PF$_6$)$_2$). Data were processed with the standard Nonius and Bruker (SAINT, APEX3) software package.[S2] Multiscan absorption correction was applied using the SADABS program.[S3] The structures were solved by intrinsic phasing[S4] and refined using the SHELXTL software package (Version 2014/6 and 2018/3).[S5] Graphical handling of the structural data during solution and refinement were performed with OLEX2.[S6] All non-hydrogen atoms were given anisotropic

displacement parameters. Hydrogen atoms bound to carbon were input at calculated positions and refined with a riding model. Hydrogen atoms bound to nitrogen were located in difference Fourier syntheses and refined, either fully or with appropriate distance and/or symmetry. CCDC No. 2045934-2045940 contain the supplementary crystallographic data for this paper. These data can be obtained free of charge from The Cambridge Crystallographic Data Centre via https://www.ccdc.cam.ac.uk/data_request/cif.

4.2 Crystal structures

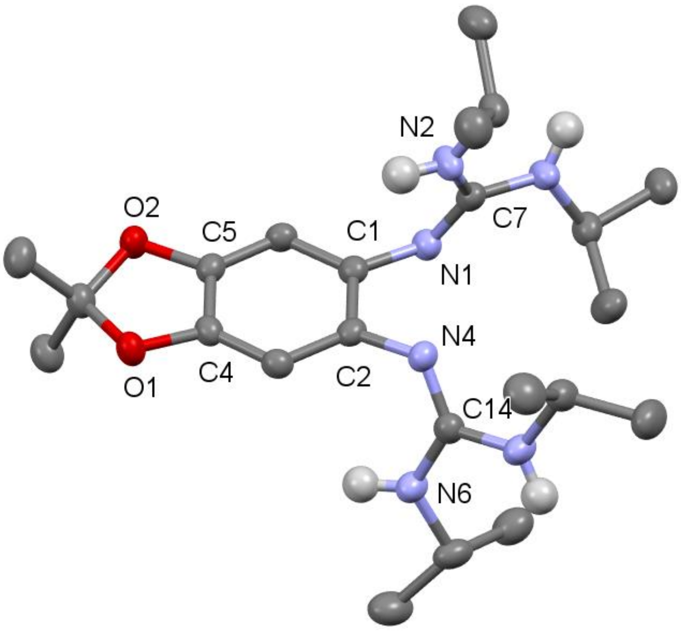

parameter	bond distance (Å)
C1-C2	1.414(3)
C1-N1	1.420(2)
C2-N4	1.407(3)
C5-O2	1.388(2)
C4-O1	1.384(2)
C4-C5	1.375(3)
N1-C7	1.295(3)
N4-C14	1.291(2)
C7-N2	1.368(3)
C14-N6	1.378(3)

Figure S29: Illustration of the structure of L3 in the solid state. Color code: N pale blue, O red, C dark grey, H pale grey. Displacement ellipsoids drawn at the 50% probability level. Hydrogen atoms bound to carbon omitted.

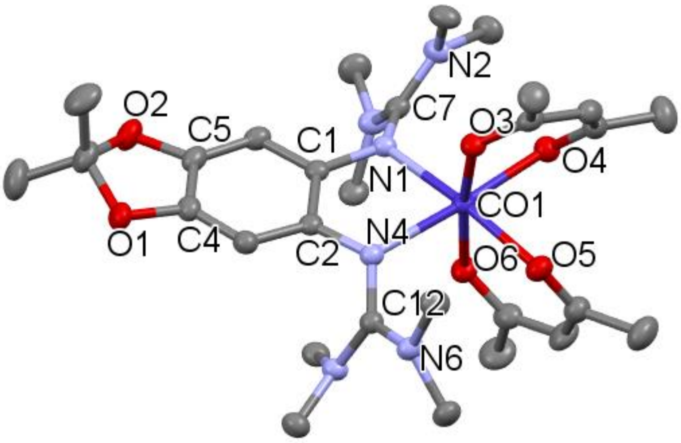

parameter	bond distance (Å)
C1-C2	1.411(3)
C1-N1	1.410(3)
C2-N4	1.406(3)
C5-O2	1.386(3)
C4-O1	1.389(3)
C4-C5	1.377(3)
N1-C7	1.325(3)
N4-C12	1.318(3)
C7-N2	1.353(3)
C12-N6	1.357(3)
N1-Co1	2.135(2)
N4-Co1	2.149(2)
O3-Co1	2.059(2)
O4-Co1	2.077(2)
O5-Co1	2.103(2)
O6-Co1	2.079(2)

Figure S30: Illustration of the structure of [Co(acac)$_2$(L1)] in the solid state. Color code: Co: dark blue N pale blue, O red, C dark grey. Displacement ellipsoids drawn at the 50% probability level. Hydrogen atoms omitted.

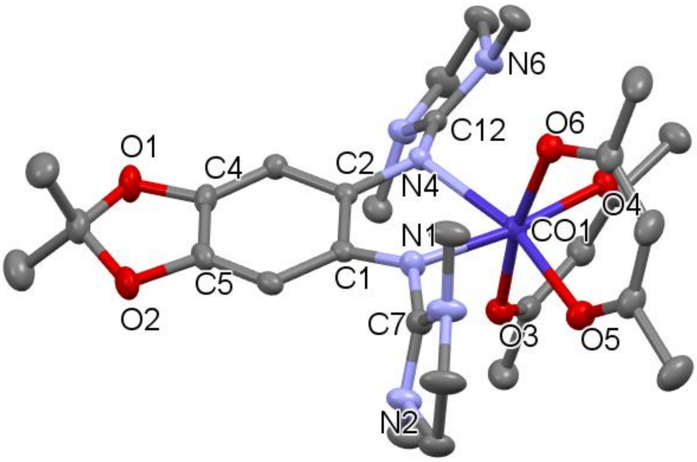

*only one of two molecules in the unit cell is shown

parameter	bond distance (Å)
C1-C2	1.418(3)
C1-N1	1.394(3)
C2-N4	1.411(3)
C5-O2	1.380(3)
C4-O1	1.387(3)
C4-C5	1.379(3)
N1-C7	1.326(3)
N4-C12	1.311(3)
C7-N2	1.354(3)
C12-N6	1.364(3)
N1-Co1	2.085(2)
N4-Co1	2.245(2)
O3-Co1	2.075(2)
O4-Co1	2.041(2)
O5-Co1	2.085(2)
O6-Co1	2.064(2)

Figure S31: Illustration of the structure of [Co(acac)$_2$(L2)] in the solid state. Color code: Co: dark blue N pale blue, O red, C dark grey. Displacement ellipsoids drawn at the 50% probability level. Hydrogen atoms omitted.

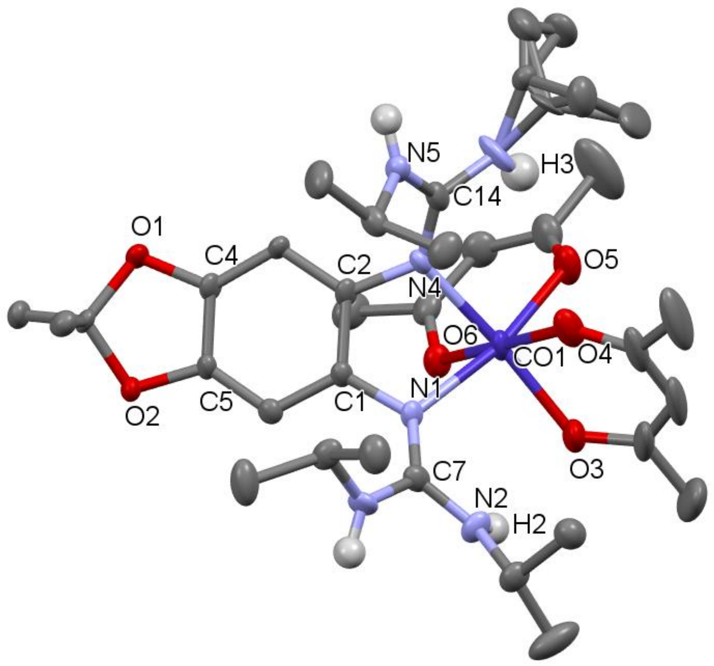

parameter	bond distance / angles (Å) / (°)
C1-C2	1.417(9)
C1-N1	1.410(8)
C2-N4	1.402(8)
C5-O2	1.390(7)
C4-O1	1.390(7)
C4-C5	1.389(9)
N1-C7	1.309(8)
N4-C14	1.332(9)
C7-N2	1.356(9)
C14-N5	1.361(9)
N1-Co1	2.161(5)
N4-Co1	2.138(6)
O3-Co1	2.079(6)
O4-Co1	2.056(5)
O5-Co1	2.106(5)
O6-Co1	2.067(5)
H2····O3	2.15(6)
H3····O5	2.22(6)
N2····O3	2.966(8)
N6····O5	3.033(9)
∢ N2-H2-O3	156(5)
∢ N6-H3-O5	154(4)

Figure S32: Illustration of the structure of [Co(acac)$_2$(L3)] in the solid state. Color code: Co: dark blue N pale blue, O red, C dark grey, H pale grey. Displacement ellipsoids drawn at the 50% probability level. Hydrogen atoms bound to carbon omitted.

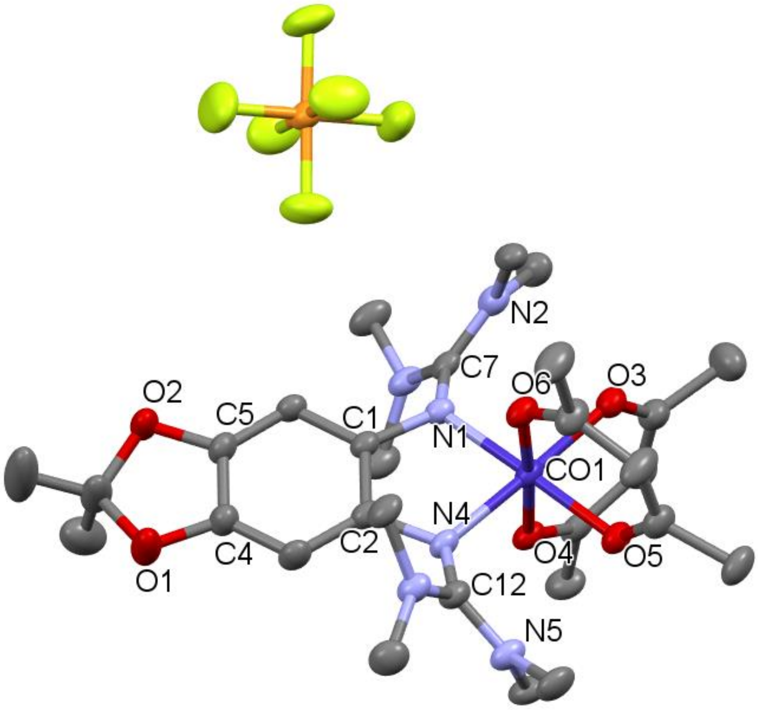

parameter	bond distance (Å)
C1-C2	1.440(4)
C1-N1	1.359(4)
C2-N4	1.365(4)
C5-O2	1.349(4)
C4-O1	1.352(4)
C4-C5	1.407(5)
N1-C7	1.382(4)
N4-C12	1.382(4)
C7-N2	1.335(5)
C12-N6	1.325(4)
N1-Co1	1.952(3)
N4-Co1	1.960(3)
O3-Co1	1.890(3)
O4-Co1	1.883(3)
O5-Co1	1.884(3)
O6-Co1	1.896(3)

Figure S33: Illustration of the structure of $[Co(acac)_2(L1)](PF_6)$ in the solid state. Color code: Co: dark blue N pale blue, O red, C dark grey, P orange, F yellow. Displacement ellipsoids drawn at the 50% probability level. Hydrogen atoms and co-crystallised solvent omitted.

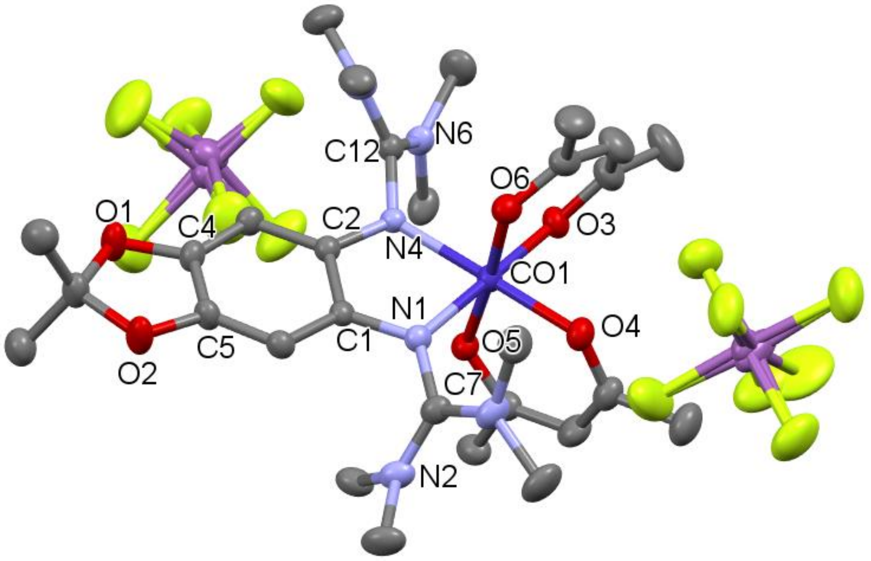

parameter	bond distance (Å)
C1-C2	1.405(5)
C1-N1	1.404(5)
C2-N4	1.424(5)
C5-O2	1.382(5)
C4-O1	1.383(5)
C4-C5	1.378(6)
N1-C7	1.347(5)
N4-C12	1.342(5)
C7-N2	1.343(5)
C12-N5	1.345(5)
N1-Co1	1.960(3)
N4-Co1	1.965(3)
O3-Co1	1.898(3)
O4-Co1	1.888(3)
O5-Co1	1.896(3)
O6-Co1	1.897(3)

Figure S34: Illustration of the structure of $[Co(acac)_2(L1)](SbF_6)_2$ in the solid state. Color code: Co: dark blue N pale blue, O red, C dark grey, Sb violet, F yellow. Displacement ellipsoids drawn at the 50% probability level. Hydrogen atoms omitted.

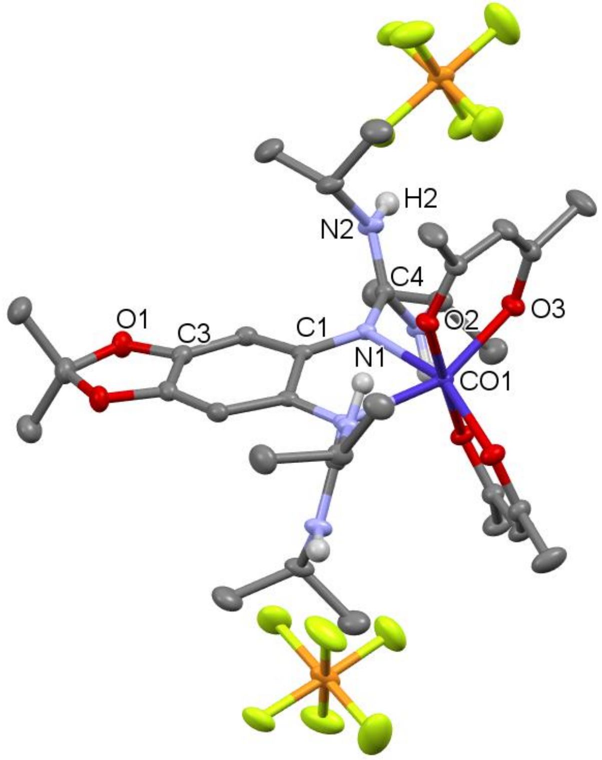

parameter	bond distance/angles (Å) / (°)
C1-C1	1.515(3)
C1-N1	1.297(2)
C3-O1	1.331(2)
C3-C3	1.454(3)
N1-C4	1.408(2)
C4-N2	1.314(2)
N1-Co1	2.175(1)
O2-Co1	2.065(1)
O3-Co1	2.020(1)
H2····O2	2.019(18)
N2····O2	2.762(2)
∢ N2-H2-O2	152.0(17)

Figure S35: Illustration of the structure of $[Co(acac)_2(L3)](PF_6)_2$ in the solid state. Color code: Co: dark blue N pale blue, O red, C dark grey, H pale grey P orange, F yellow. Displacement ellipsoids drawn at the 50% probability level. Hydrogen atoms bound to carbon omitted.

4.2 Details of the crystal structure determinations

	**L3**	**[Co(acac)$_2$(L1)]**	**[Co(acac)$_2$(L2)]**	**[Co(acac)$_2$(L3)]**
CCDC-No.	2045940	2045934	2045938	2045935
formula	$C_{23}H_{40}N_6O_2$	$C_{29}H_{46}CoN_6O_6$	$C_{29}H_{42}CoN_6O_6$	$C_{33}H_{54}CoN_6O_6$
Crystal system	orthorhombic	monoclinic	triclinic	monoclinic
Space group	*Pbca*	*P* 2$_1$/*c*	$P\bar{1}$	*C*2/*c*
a /Å	10.457(2)	12.8561(9)	11.4102(12)	43.053(4)
b /Å	18.792(4)	13.1570(8)	11.8078(12)	8.8518(8)
c /Å	26.356(5)	19.8139(14)	23.494(3)	19.7946(18)
α /°	90	90	78.919(4)	90
β /°	90	100.675(3)	86.565(4)	104.193(4)
γ /°	90	90	89.981(4)	90
V /Å^3	5179.2(18)	3293.5(4)	3100.5(6)	7313.3(12)
Z	8	4	4	8
M_r	432.61	633.65	629.61	689.75
F_{000}	1888	1348	1332	2952
d_c /Mg·m^{-3}	1.110	1.278	1.349	1.253
μ /mm^{-1}	0.073	0.569	0.604	0.518
max., min. transmission factors	1.0000, 0.7598	0.7461, 0.6537	0.7461, 0.7001	0.7458, 0.5788
X-radiation, λ /Å	Mo-K_a 0.71073	Mo-K_a, 0.71073	Mo-K_a 0.71073	Mo-K_a 0.71073
data collect. temperat./K	120	100	100	100
θ range /°	2.167 to 30.109	2.092 to 28.997	1.946 to 27.500	1.952 to 27.498
index ranges *h,k,l*	-14 ... 14, -26 ... 26, -37 ... 37	-17 ... 17, -17 ... 17, -27 ... 27	-14 ... 14, -15 ... 15, -30 ... 30	-55 ... 55, -11 ... 11, -25 ... 25
reflections measured	101295	114234	153937	25566
Unique [R_{int}]	7611[0.0941]	8742 [0.0692]	14238[0.0420]	8392[0.1022]
observed [$I \geq 2\sigma(I)$]	4549	6818	12286	5032
data / restraints /parameters	7611 / 0 / 290	8742 / 0 / 393	14238 / 0 / 777	8392 / 0 / 459
GooF on F^2	1.022	1.015	1.044	1.091
R indices[$F>4\sigma(F)$] $R(F)$, $wR(F^2)$	0.0577, 0.1272	0.0467, 0.1157	0.0378, 0.0889	0.1093, 0.2272
R indices (all data) $R(F)$, $wR(F^2)$	0.1134, 0.1507	0.0642, 0.1273	0.0464, 0.0945	0.1643, 0.2515
largest residual peaks /e·Å^{-3}	0.621, -0.502	2.013, -0.597	0.919, -0.597	0.766, -1.004

	**[Co(acac)$_2$(L1)](PF$_6$)**	**[Co(acac)$_2$(L1)](SbF$_6$)$_2$**	**[Co(acac)$_2$(L3)](PF$_6$)$_2$**
CCDC-No.	2045936	2045939	2045937
formula	$C_{29}H_{46}CoF_6N_6O_6P$	$C_{29}H_{46}CoF_{12}N_6O_6Sb_2$	$C_{33}H_{54}CoF_{12}N_6O_6P_2$
Crystal system	monoclinic	monoclinic	tetragonal
Space group	$P2_1/c$	$P2_1/c$	$I4_1/a$
a /Å	8.2990(7)	17.0024(6)	16.2400(17)
b /Å	21.252(2)	14.2413(6)	16.2400(17)
c /Å	24.161(2)	20.8020(8)	34.667(3)
α /°	90	90	90
β /°	90.389(3)	96.713(2)	90
γ /°	90	90	90
V /Å^3	4261.1(7)	5002.4(3)	9143(2)
Z	4	4	8
M_r	829.57	1249.35	979.69
F_{000}	1725	2508	4056
d_c /Mg·m^{-3}	1.293	1.659	1.423
μ /mm^{-1}	0.584	1.496	0.540
max., min. transmission factors	0.7454, 0.6326	0.7461, 0.6308	0.7461, 0.6879
X-radiation, λ /Å	Mo-K_a, 0.71073	Mo-K_a 0.71073	Mo-K_a 0.71073
data collect. temperat./K	100	100	100
θ range /°	1.917 to 26.502	1.971 to 28.000	2.127 to 29.999
index ranges h,k,l	-10 ... 10, -26 ... 26, -30 ... 30	-22 ... 21, -18 ... 18, -27 ... 27	-22 ... 22, -22 ... 22, -48 ... 48
reflections measured	207659	123510	248306
Unique [R_{int}]	8797[0.1356]	12082[0.0737]	6683[0.0770]
observed [$I \geq 2\sigma(I)$]	6843	9704	5473
data / restraints /parameters	8797 / 1 / 502	9704 / 1 / 639	6683 / 0 / 279
GooF on F^2	1.062	1.046	1.061
R indices[$F>4\sigma(F)$] $R(F)$, $wR(F^2)$	0.0615, 0.1643	0.0410, 0.1000	0.0314, 0.0793
R indices (all data) $R(F)$, $wR(F^2)$	0.0790, 0.1755	0.0558, 0.1108	0.0436, 0.0836
largest residual peaks /e·Å^{-3}	0.809, -0.472	1.154, -0.620	0.467, -0.444

5 DFT calculations

DFT calculations were carried out with the TURBOMOLE program package.[S7] The B3LYP functional[S8] in combination with the def2-TZVP basis set[S9] was used in all calculations. Structural optimizations were performed at the RI-DFT[S10] level of theory with multipole accelerated RI-approximation (MARI-J)[S11]. Dispersion is included by the DFT-D3[S12] approach developed by Grimme and co-workers. The solvent effect was simulated with the conductor-like screening model (COSMO). For all structures calculated at $\varepsilon_r = 1$ (without COSMO), a vibrational analysis was carried out to confirm that the optimized structures are energy minima on the potential energy hypersurface. The atomic spin population of a given atom, as obtained from a natural population analysis (NPA)[S13], is defined as the difference between the population of α and β electrons. The spin density distributions were visualized with GaussView 5. The deviation between the experimentally derived and calculated structures are analysed with the program aRMSD.[S14]

5.1 Calculated structures and spin densities

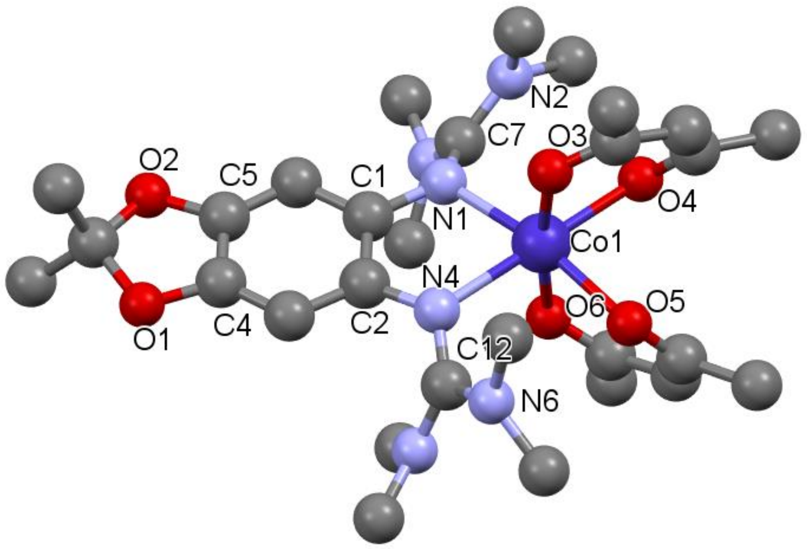

parameter	bond distance (Å)
C1-C2	1.412
C1-N1	1.399
C2-N4	1.400
C5-O2	1.375
C4-O1	1.375
C4-C5	1.383
N1-C7	1.312
N4-C12	1.313
C7-N2	1.362
C12-N6	1.359
N1-Co1	2.171
N4-Co1	2.164
O3-Co1	2.059
O4-Co1	2.097
O5-Co1	2.128
O6-Co1	2.123

Figure S36: Illustration of the calculated structure of [Co(acac)$_2$(L1)]. E_{el} = -3295.181662114 H; Chem pot = 1706.88 kJ mol^{-1} (1 bar, 298 K).

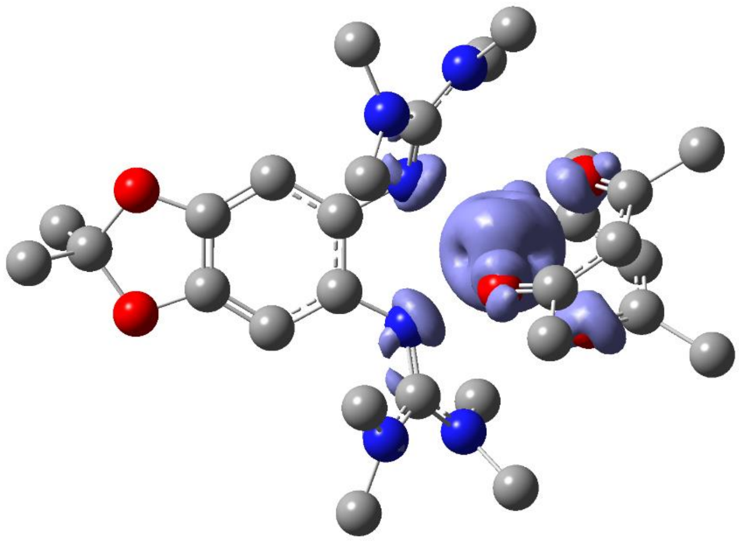

fragment	population
Co	2.692
$(acac)_2$	0.211
L1	0.097

Figure S37: Spin density plot and natural population analysis results for $[Co(acac)_2(L1)]$.

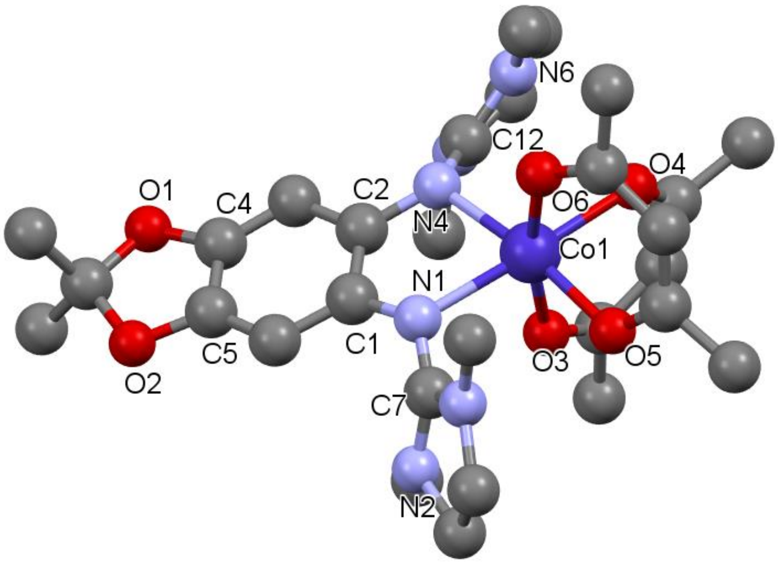

parameter	bond distance (Å)
C1-C2	1.415
C1-N1	1.396
C2-N4	1.401
C5-O2	1.376
C4-O1	1.376
C4-C5	1.383
N1-C7	1.309
N4-C12	1.309
C7-N2	1.381
C12-N6	1.353
N1-Co1	2.166
N4-Co1	2.173
O3-Co1	2.118
O4-Co1	2.107
O5-Co1	2.101
O6-Co1	2.083

Figure S38: Illustration of the calculated structure of $[Co(acac)_2(L2)]$. E_{el} = -3292.787175053 H; Chem pot = 1598.17 kJ mol^{-1} (1 bar, 298 K).

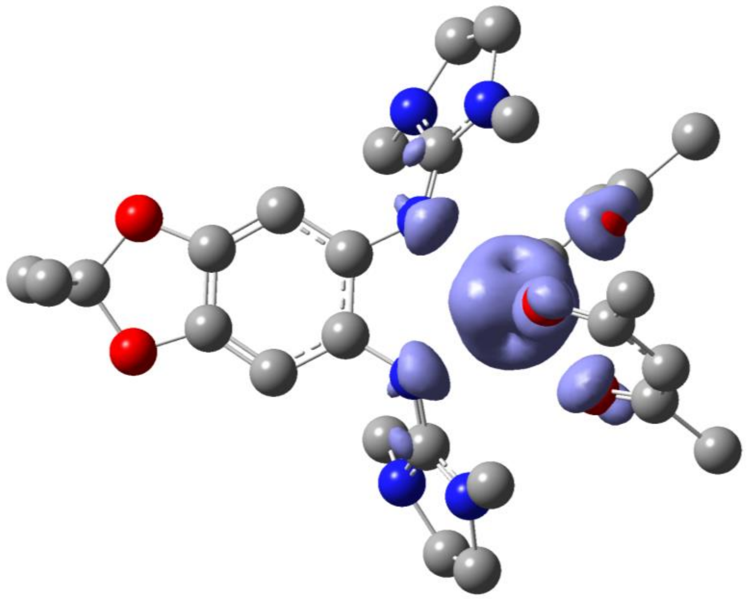

fragment	population
Co	2.698
$(acac)_2$	0.195
L2	0.106

Figure S39: Spin density plot and natural population analysis results for $[Co(acac)_2(L2)]$.

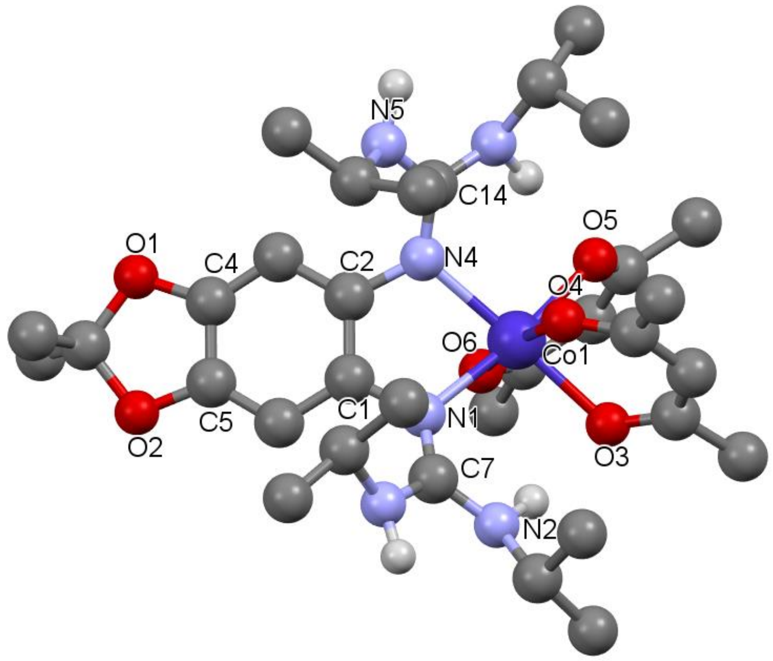

parameter	bond distance (Å)
C1-C2	1.410
C1-N1	1.400
C2-N4	1.403
C5-O2	1.374
C4-O1	1.374
C4-C5	1.386
N1-C7	1.306
N4-C14	1.308
C7-N2	1.361
C14-N5	1.375
N1-Co1	2.176
N4-Co1	2.142
O3-Co1	2.115
O4-Co1	2.122
O5-Co1	2.110
O6-Co1	2.066
H-Bond O5	1.877
H-Bond O3	1.963

Figure S40: Illustration of the calculated structure of $[Co(acac)_2(L3)]$. E_{el} = -3452.461705414 H; Chem pot = 1982.42 kJ mol^{-1} (1 bar, 298 K).

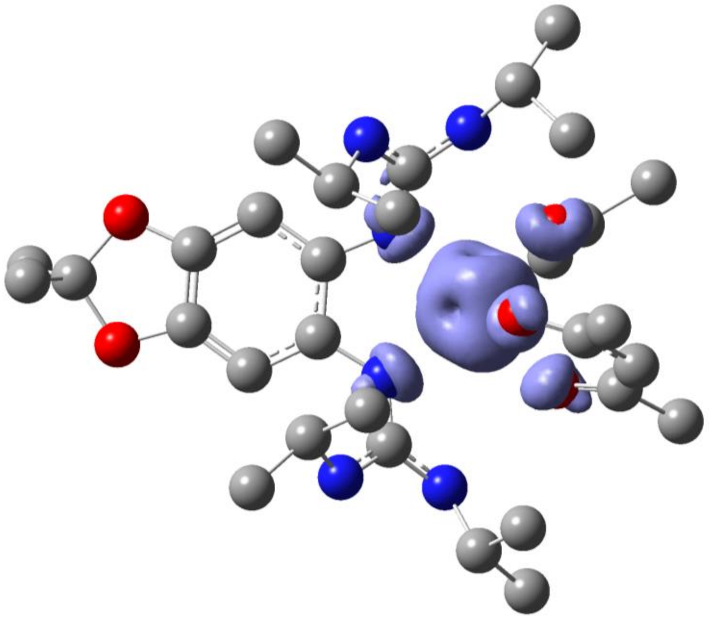

fragment	population
Co	2.699
$(acac)_2$	0.207
L3	0.094

Figure S41: Spin density plot and natural population analysis results for $[Co(acac)_2(L3)]$.

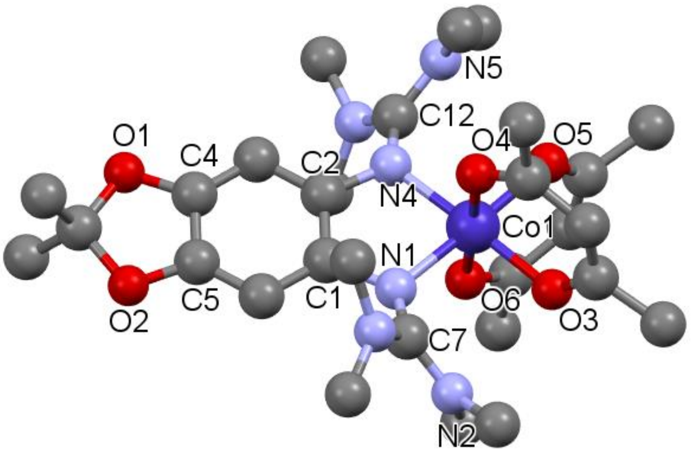

parameter	bond distance (Å)
C1-C2	1.400
C1-N1	1.405
C2-N4	1.405
C5-O2	1.362
C4-O1	1.362
C4-C5	1.389
N1-C7	1.332
N4-C12	1.332
C7-N2	1.351
C12-N5	1.351
N1-Co1	1.976
N4-Co1	1.977
O3-Co1	1.917
O4-Co1	1.908
O5-Co1	1.917
O6-Co1	1.908

Figure S42: Illustration of the calculated structure of $[Co(III)(acac)_2(L1)]^+$. E_{el} = -3294.997794527 H; Chem pot = 1731.64 kJ mol^{-1} (1 bar, 298 K).

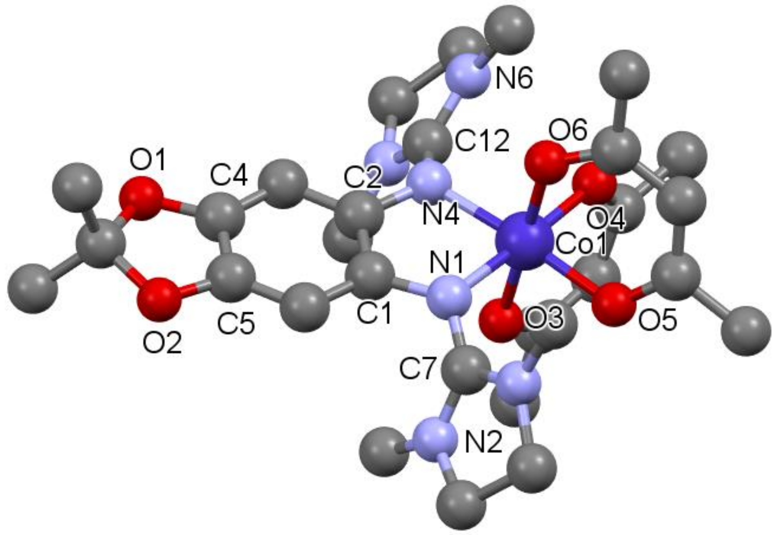

parameter	bond distance (Å)
C1-C2	1.398
C1-N1	1.409
C2-N4	1.409
C5-O2	1.365
C4-O1	1.365
C4-C5	1.388
N1-C7	1.331
N4-C12	1.328
C7-N2	1.352
C12-N6	1.346
N1-Co1	1.978
N4-Co1	1.976
O3-Co1	1.922
O4-Co1	1.910
O5-Co1	1.909
O6-Co1	1.902

Figure S43: Illustration of the calculated structure of $[Co(III)(acac)_2(L2)]^+$. E_{el} = -3292.600949450 H; Chem pot = 1625.06 kJ mol^{-1} (1 bar, 298 K).

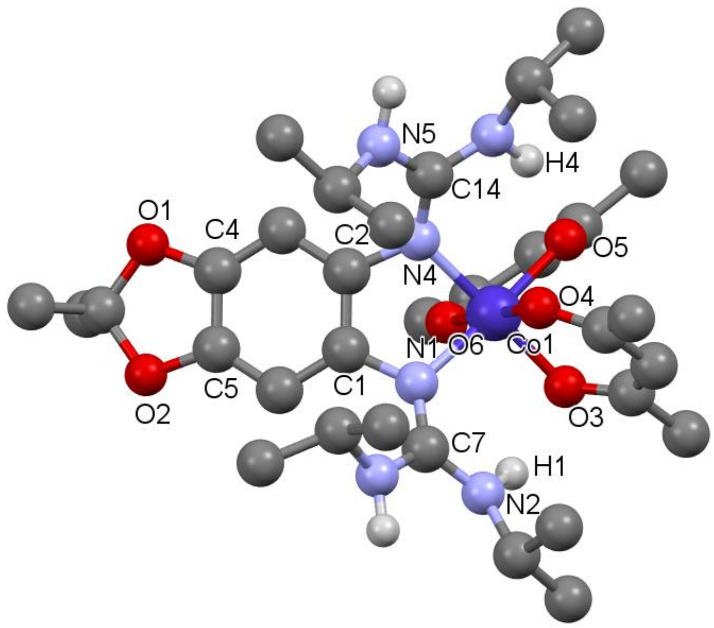

parameter	bond distance (Å)
C1-C2	1.396
C1-N1	1.411
C2-N4	1.411
C5-O2	1.362
C4-O1	1.362
C4-C5	1.392
N1-C7	1.324
N4-C14	1.324
C7-N2	1.347
C14-N5	1.364
N1-Co1	1.979
N4-Co1	1.971
O3-Co1	1.927
O4-Co1	1.918
O5-Co1	1.925
O6-Co1	1.898
H-bond O5	1.814
H-bond O3	1.960

Figure S44: Illustration of the calculated structure of $[Co(III)(acac)_2(L3)]^+$. E_{el} = -3452.276000388 H; Chem pot = 2008.69 kJ mol^{-1} (1 bar, 298 K).

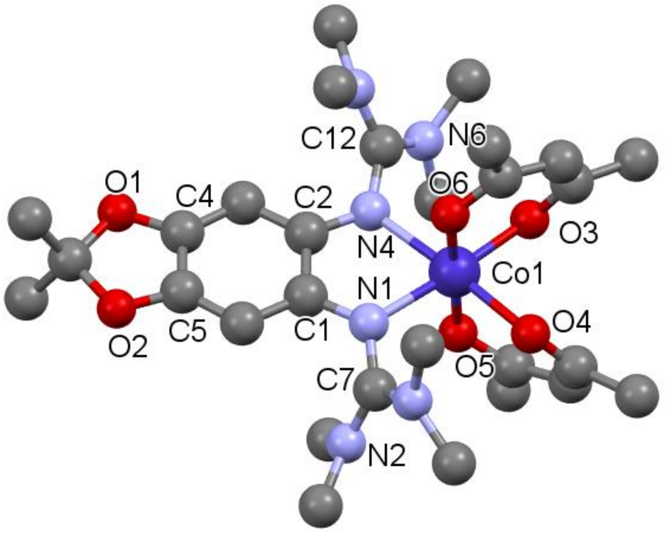

parameter	bond distance (Å)
C1-C2	1.460
C1-N1	1.361
C2-N4	1.352
C5-O2	1.332
C4-O1	1.334
C4-C5	1.421
N1-C7	1.367
N4-C12	1.357
C7-N2	1.346
C12-N6	1.338
N1-Co1	2.024
N4-Co1	2.196
O3-Co1	1.913
O4-Co1	2.110
O5-Co1	1.877
O6-Co1	1.883

Figure S45: Illustration of the calculated structure of [*is*-Co(III)(acac)$_2$(L1$^{\cdot+}$)]$^{2+}$. E_{el} = -3294.682347603 H; Chem pot = 1724.33 kJ mol^{-1} (1 bar, 298 K).

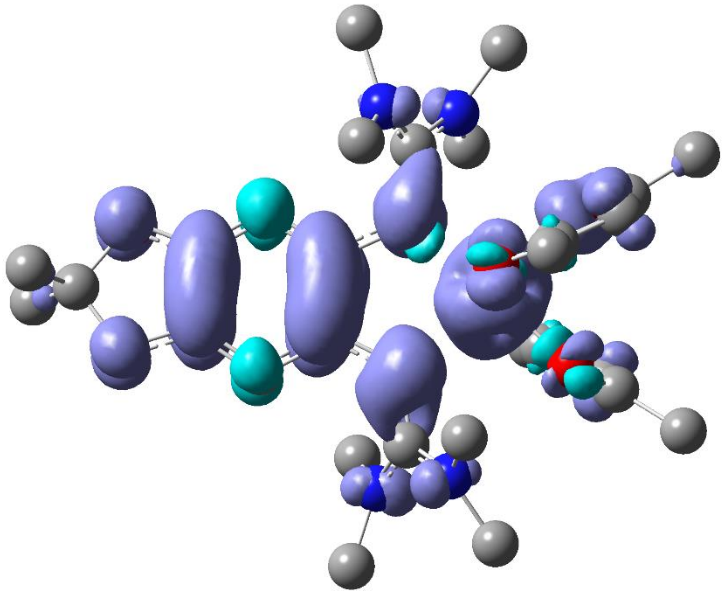

fragment	population
Co	1.800
(acac)$_2$	0.153
L1	1.047

Figure S46: Spin density plot and natural population analysis results for [*is*-Co(III)(acac)$_2$(L1$^{\cdot+}$)]$^{2+}$.

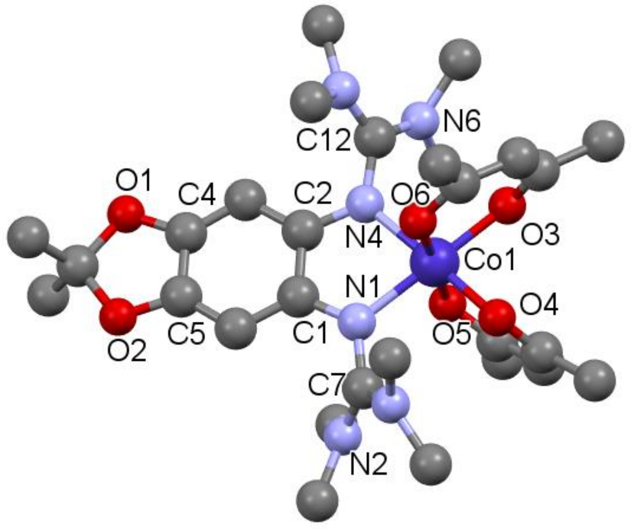

parameter	bond distance (Å)
C1-C2	1.453
C1-N1	1.359
C2-N4	1.359
C5-O2	1.332
C4-O1	1.332
C4-C5	1.421
N1-C7	1.370
N4-C12	1.370
C7-N2	1.347
C12-N6	1.333
N1-Co1	1.989
N4-Co1	1.988
O3-Co1	1.895
O4-Co1	1.895
O5-Co1	1.905
O6-Co1	1.905

Figure S47: Illustration of the calculated structure of $[Co(III)(acac)_2(L1^{\cdot+})]^{2+}$. E_{el} = - 3294.706755596 H; Chem pot = 1737.86 kJ mol^{-1} (1 bar, 298 K).

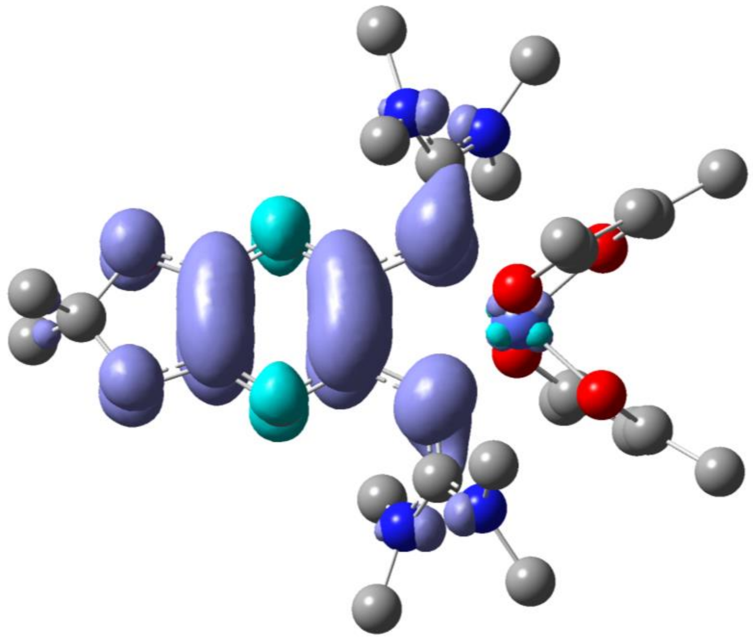

fragment	population
Co	0.006
$(acac)_2$	0.012
L1	0.982

Figure S48: Spin density plot and natural population analysis results for $[Co(III)(acac)_2(L1^{\cdot+})]^{2+}$.

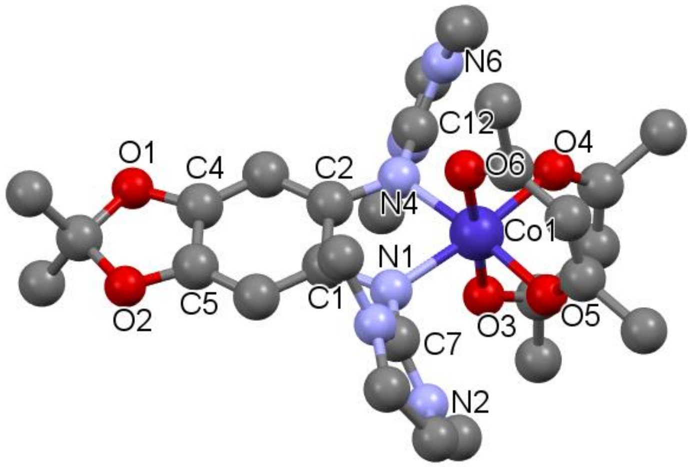

parameter	bond distance (Å)
C1-C2	1.453
C1-N1	1.355
C2-N4	1.355
C5-O2	1.333
C4-O1	1.333
C4-C5	1.422
N1-C7	1.361
N4-C12	1.361
C7-N2	1.328
C12-N6	1.328
N1-Co1	1.974
N4-Co1	1.975
O3-Co1	1.907
O4-Co1	1.889
O5-Co1	1.889
O6-Co1	1.907

Figure S49: Illustration of the calculated structure of $[Co(III)(acac)_2(L2^{\cdot+})]^{2+}$. E_{el} = -3292.321355562 H; Chem pot = 1626.62 kJ mol^{-1} (1 bar, 298 K).

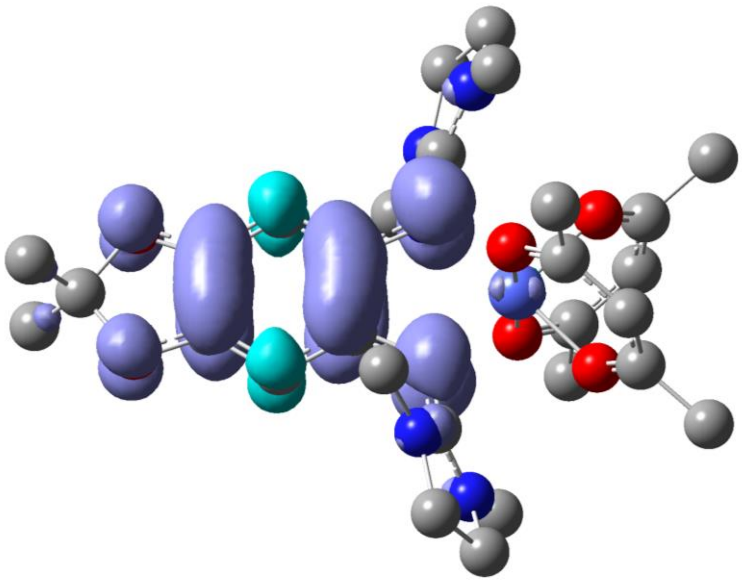

fragment	population
Co	0.010
$(acac)_2$	0.009
L2	0.981

Figure S50: Spin density plot and natural population analysis results for $[Co(III)(acac)_2(L2^{\cdot+})]^{2+}$.

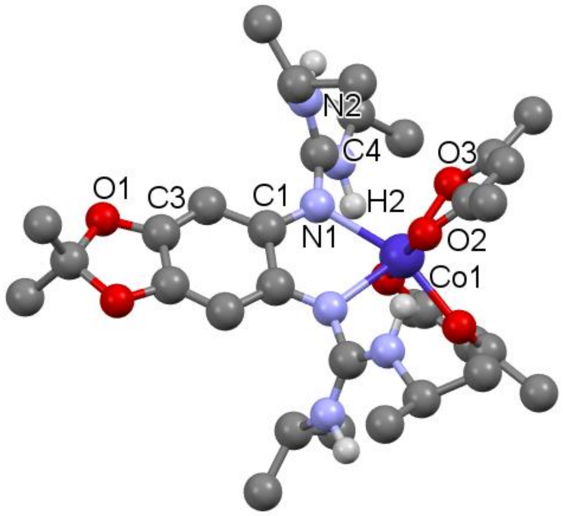

parameter	bond distance (Å)
C1-C1	1.515
C1-N1	1.298
C3-O1	1.321
C3-C3	1.457
N1-C4	1.392
C4-N2	1.329
N1-Co1	2.183
O2-Co1	2.096
O3-Co1	2.005
H-Bond O2	1.824

Figure S51: Illustration of the calculated structure of $[Co(II)(acac)_2(L3^{2+})]^{2+}$. E_{el} = -3451.984737409 H; Chem pot = 1991.84 kJ mol^{-1} (1 bar, 298 K).

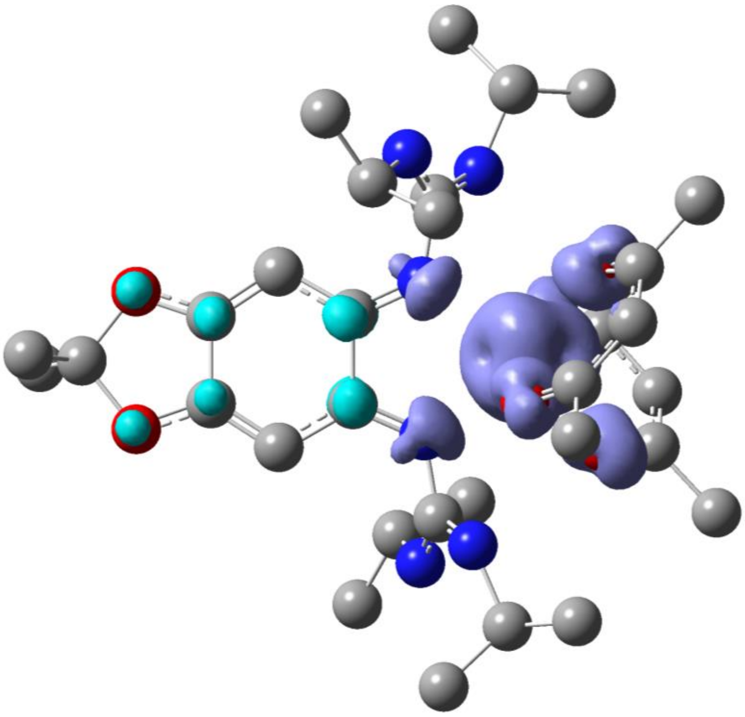

fragment	population
Co	2.720
$(acac)_2$	0.288
L3	-0.008

Figure S52: Spin density plot and natural population analysis results for $[Co(III)(acac)_2(L3^{2+})]^{2+}$.

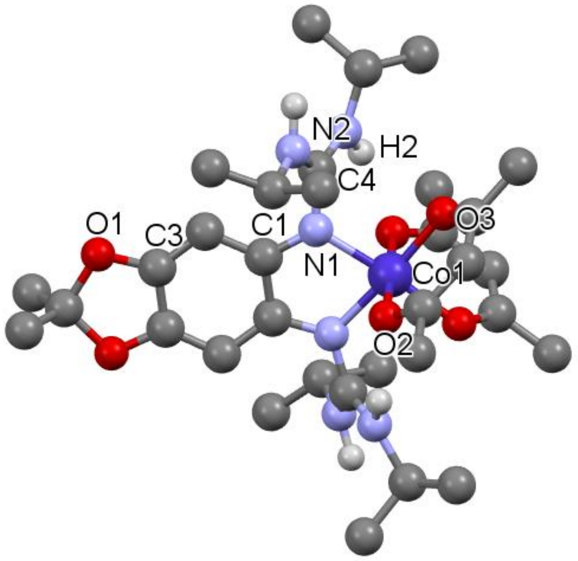

parameter	bond distance (Å)
C1-C1	1.454
C1-N1	1.353
C3-O1	1.335
C3-C3	1.420
N1-C4	1.378
C4-N2	1.329
N1-Co1	1.961
O2-Co1	1.912
O3-Co1	1.898
H-bond O2	2.061

Figure S53: Illustration of the calculated structure of $[Co(III)(acac)_2(L3^{\cdot +})]^{2+}$. E_{el} = -3451.991317744 H; Chem pot = 2004.86 kJ mol^{-1} (1 bar, 298 K).

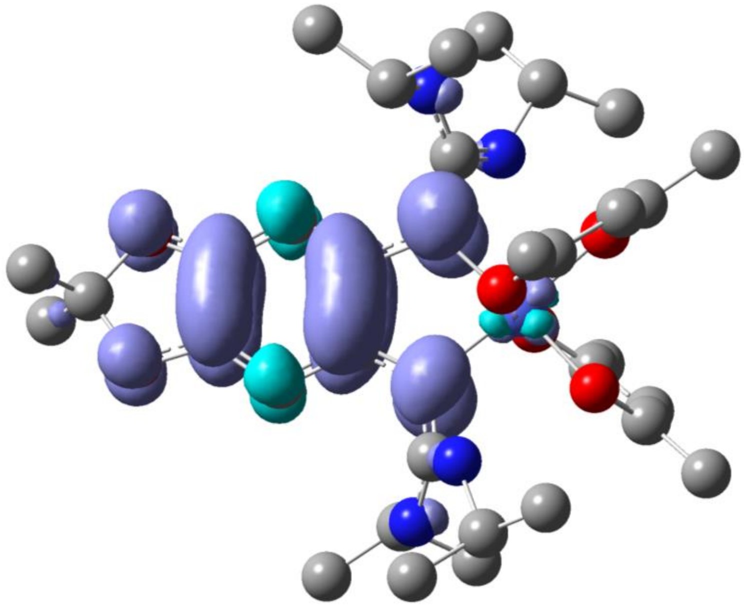

fragment	population
Co	0.018
$(acac)_2$	0.015
L3	0.966

Figure S54: Spin density plot and natural population analysis results for $[Co(III)(acac)_2(L3^{\cdot +})]^{2+}$.

5.2 Comparison of H-bond lengths of calculated and measured structures

Structure	Calculated H-bond 1 distance (N-O distance) [Å]	Calculated H-bond 2 distance (N-O distance) [Å]	Measured H-bond 1 Distance (N-O distance) [Å]	Measured H-bond 2 distance (N-O distance) [Å]
$[Co(acac)_2(L3)]$	1.963(2.905)	1.877(2.835)	2.216(3.033)	2.148(2.966)
$[Co(III)(acac)_2(L3)]^{+}$	1.960(2.848)	1.814(2.771)	-	-
$[Co(III)(acac)_2(L3)]^{2+}$	2.061(2.952)	2.061(2.952)	-	-
$[Co(II)(acac)_2(L3)]^{2+}$	1.824(2.788)	1.824(2.788)	2.019(2.762)	2.019(2.762)

5.3 Calculations of the Gibbs free energy for ligand exchange reactions

To compare the bonding strength of the different ligands, the gibbs free energy of selected ligand exchange reactions based on the 5.3 shown structures were calculated. The Gibbs free energy is calculated at 273.15 K and 1 bar.

$$[Co(III)(acac)_2(L1^{\cdot+})]^{2+} + L3 \longrightarrow [Co(II)(acac)_2(L3^{2+})]^{2+} + L1$$

$\Delta G = -25.16\ kJ\ mol^{-1}$

$$[Co(III)(acac)_2(L2^{\cdot+})]^{2+} + L3 \longrightarrow [Co(II)(acac)_2(L3^{2+})]^{2+} + L2$$

$\Delta G = -29.75\ kJ\ mol^{-1}$

$$[Co(II)(acac)_2(L1)] + L3 \longrightarrow [Co(II)(acac)_2(L3)] + L1$$

$\Delta G = -9.01\ kJ\ mol^{-1}$

$$[Co(II)(acac)_2(L2)] + L3 \longrightarrow [Co(II)(acac)_2(L3)] + L2$$

$\Delta G = -40.00\ kJ\ mol^{-1}$

$$[\textit{is}\text{-}Co(III)(acac)_2(L1^{\bullet+})]^{2+} + L3 \longrightarrow [Co(II)(acac)_2(L3^{2+})]^{2+} + L1$$

$\Delta G = -76.24\ kJ\ mol^{-1}$

5.4 aRMSD calculations

The crystal structure of [Co(acac)$_2$(L1)] is in good agreement with that calculated, with a total RMSD of 0.41 Å. All bond distances are in excellent agreement, as evidenced with the RMSE of 0.012 Å (Figure S56).

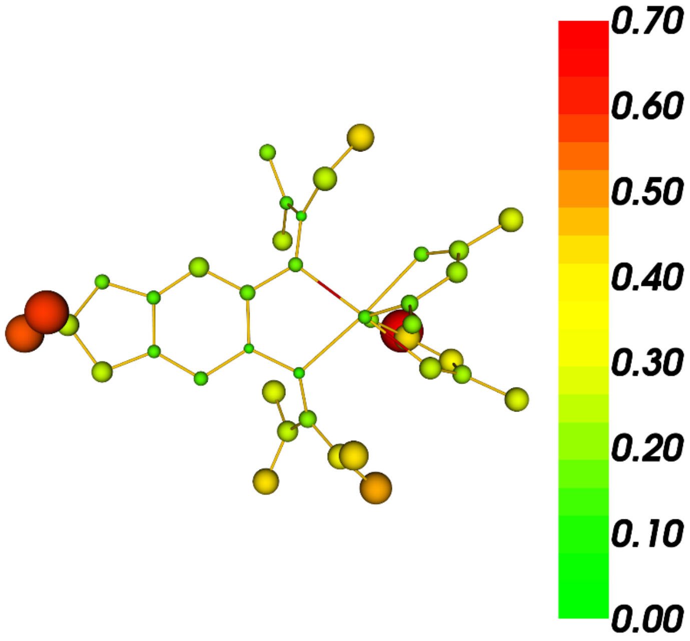

Figure S55: Superposition with Root-Mean-Square-Deviation (RMSD) for the experimentally determined and calculated structures of [Co(acac)$_2$(L1)]. The sphere dimensions reflect the relative RMSD distribution and the color code the absolute deviation (small for green color and large for red color).

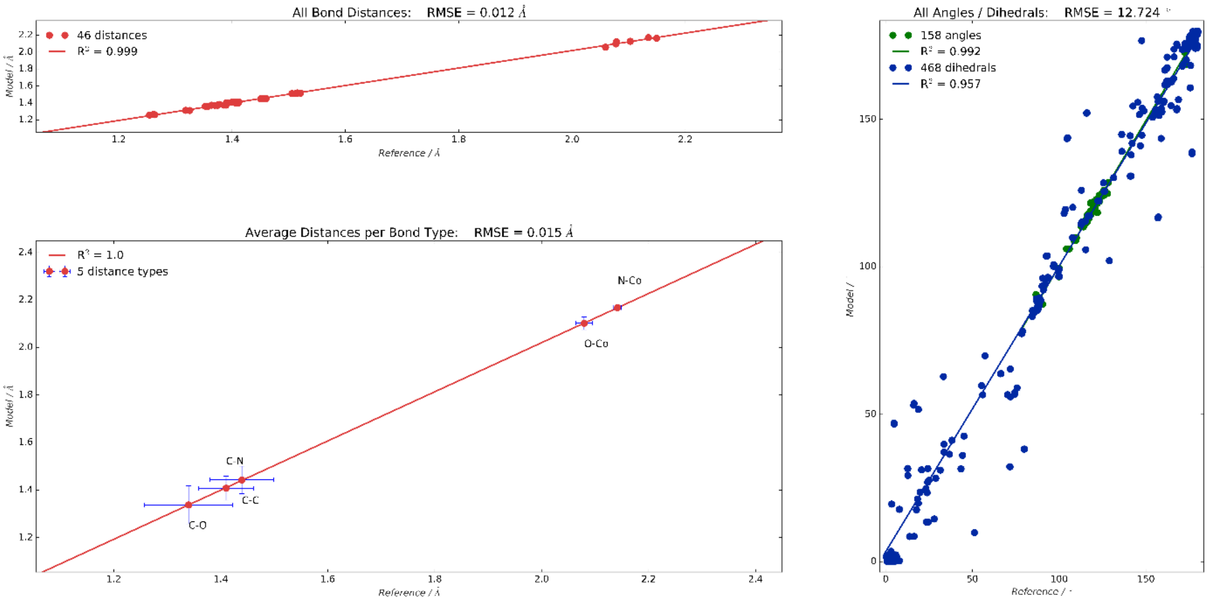

Figure S56: Detailed analysis of the Root-Mean-Square-Error (RMSE) for bonds and angles for the experimentally determined structure of [Co(acac)$_2$(L1)] (Model) and the calculated structure (Reference).

The crystal structure of [Co(acac)$_2$(L2)] is in good agreement with that calculated, with a total RMSD of 0.53 Å. All bond distances are in excellent agreement, as evidenced with the RMSE of 0.021 Å (Figure S58).

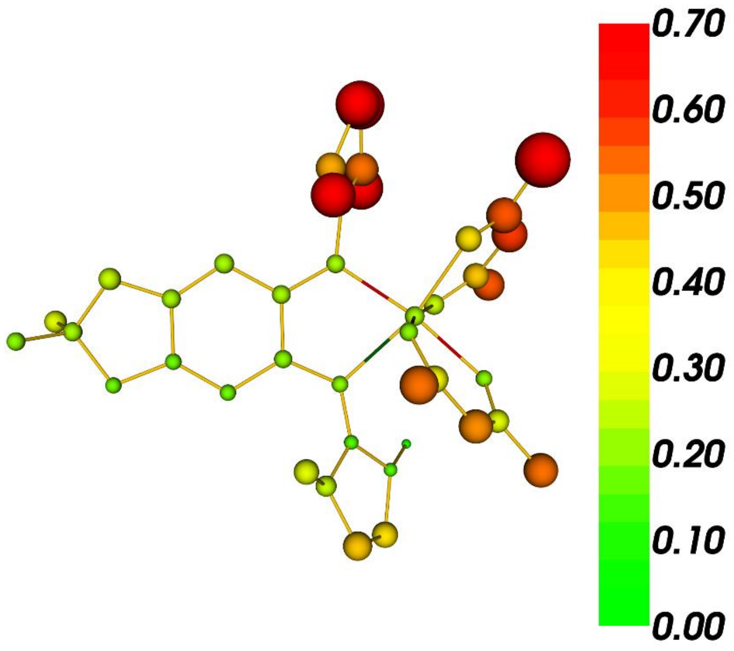

Figure S57: Superposition with Root-Mean-Square-Deviation (RMSD) for the experimentally determined structure of [Co(acac)$_2$(L2)] and the calculated structure.

The sphere dimensions reflect the relative RMSD distribution and the color code the absolute deviation (small for green color and large for red color).

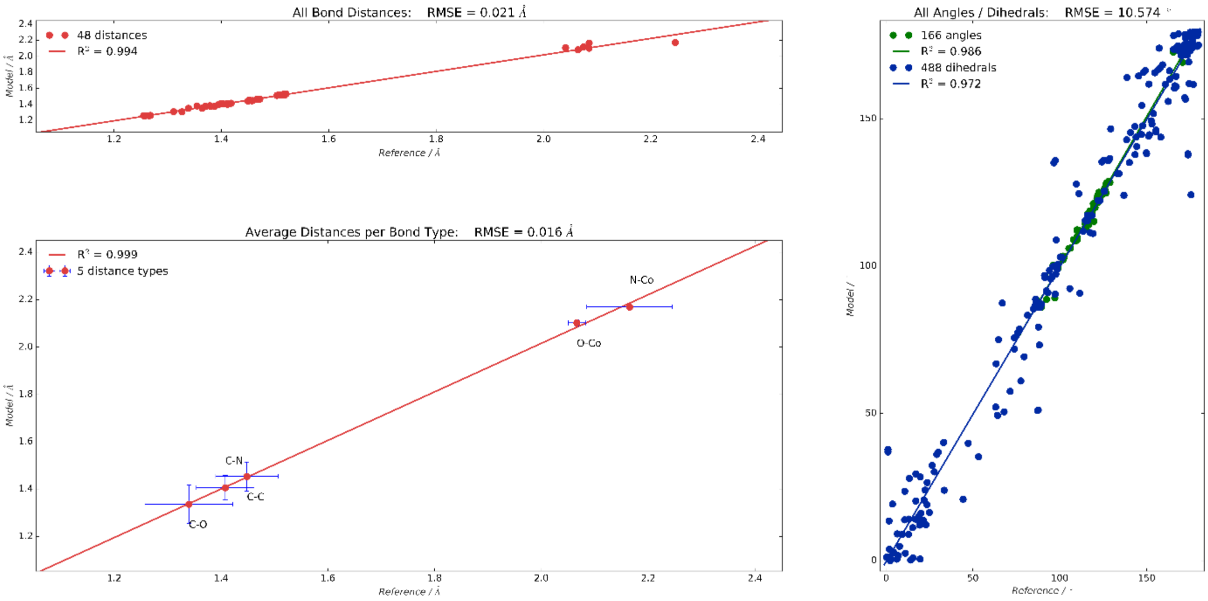

Figure S58: Detailed analysis of the Root-Mean-Square-Error (RMSE) for bonds and angles for the experimentally determined structure of [Co(acac)$_2$(L2)] (Model) and the calculated structure (Reference).

The crystal structure of [Co(acac)$_2$(L3)] is in excellent agreement with that calculated, with a total RMSD of 0.05 Å. All bond distances are in excellent agreement, as evidenced with the RMSE of 0.001 Å (Figure S60).

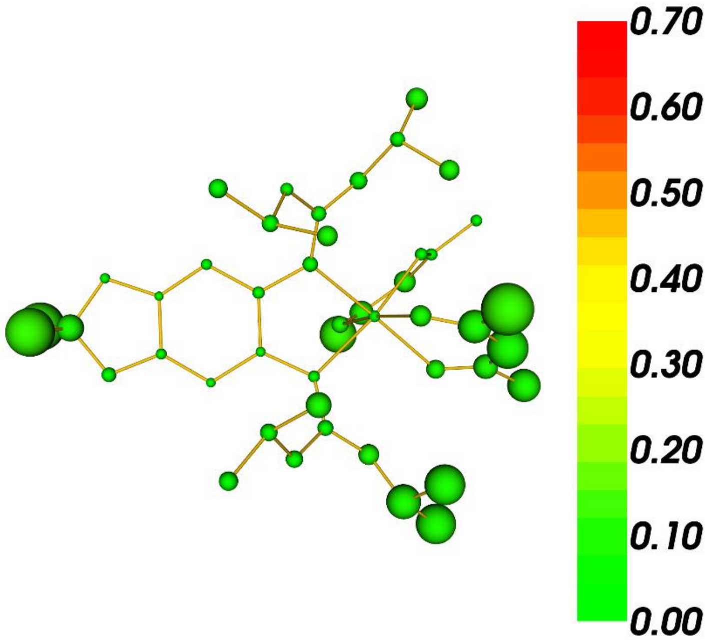

Figure S59: Superposition with Root-Mean-Square-Deviation (RMSD) for the experimentally determined and calculated structures of [Co(acac)$_2$(L3)] and. The sphere dimensions reflect the relative RMSD distribution and the color code the absolute deviation (small for green color and large for red color).

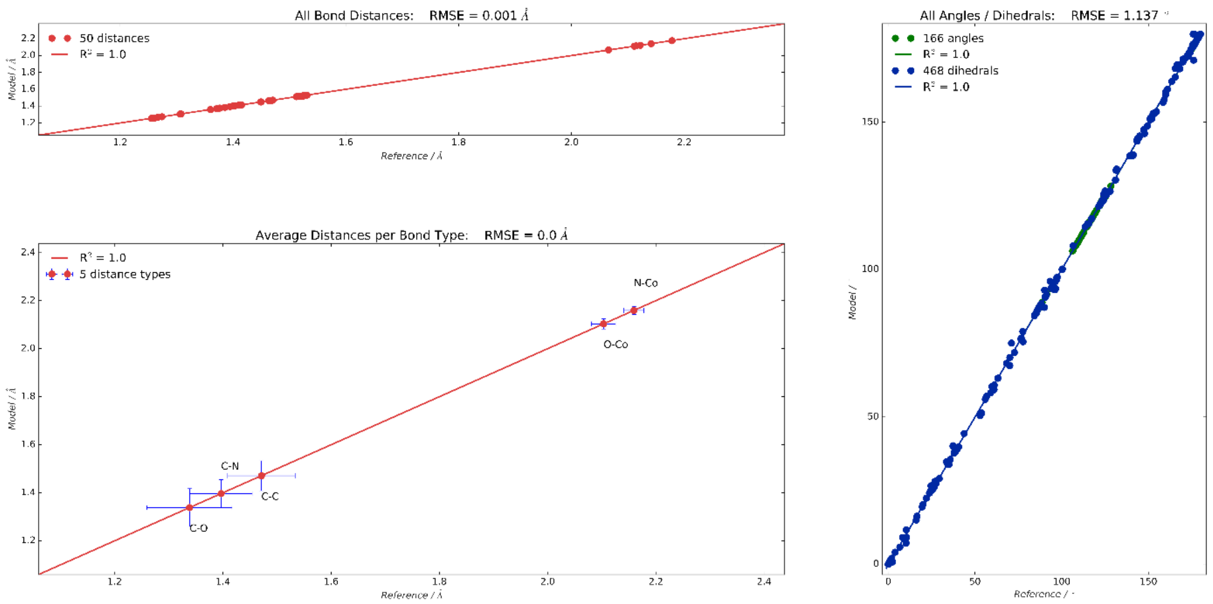

Figure S60: Detailed analysis of the Root-Mean-Square-Error (RMSE) for bonds and angles for the experimentally determined structure of [Co(acac)$_2$(L3)] (Model) and the calculated structure (Reference).

The crystal structure of [Co(acac)$_2$(L1)](PF$_6$) is in good agreement with that calculated, with a total RMSD of 0.25 Å. All bond distances are in excellent agreement, as evidenced with the RMSE of 0.015 Å (Figure S62).

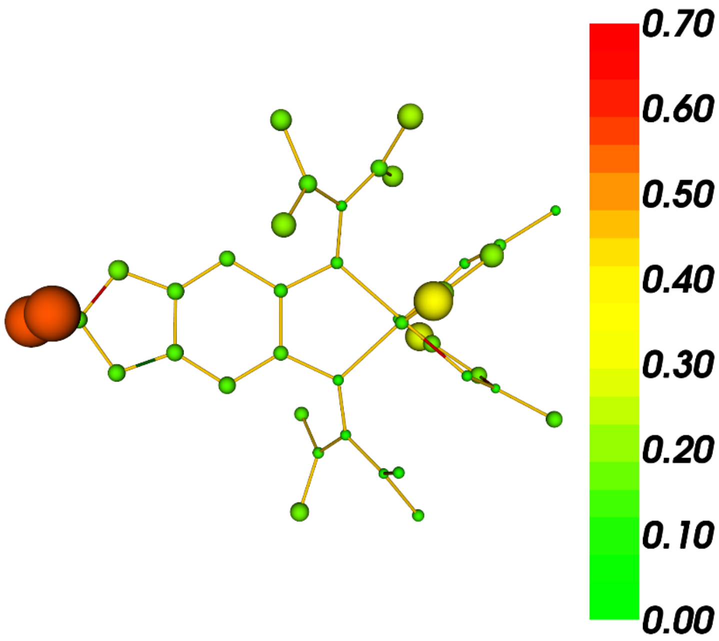

Figure S61: Superposition with Root-Mean-Square-Deviation (RMSD) for the experimentally determined structure of [Co(acac)$_2$(L1)](PF$_6$) and the calculated structure. The sphere dimensions reflect the relative RMSD distribution and the color code the absolute deviation (small for green color and large for red color).

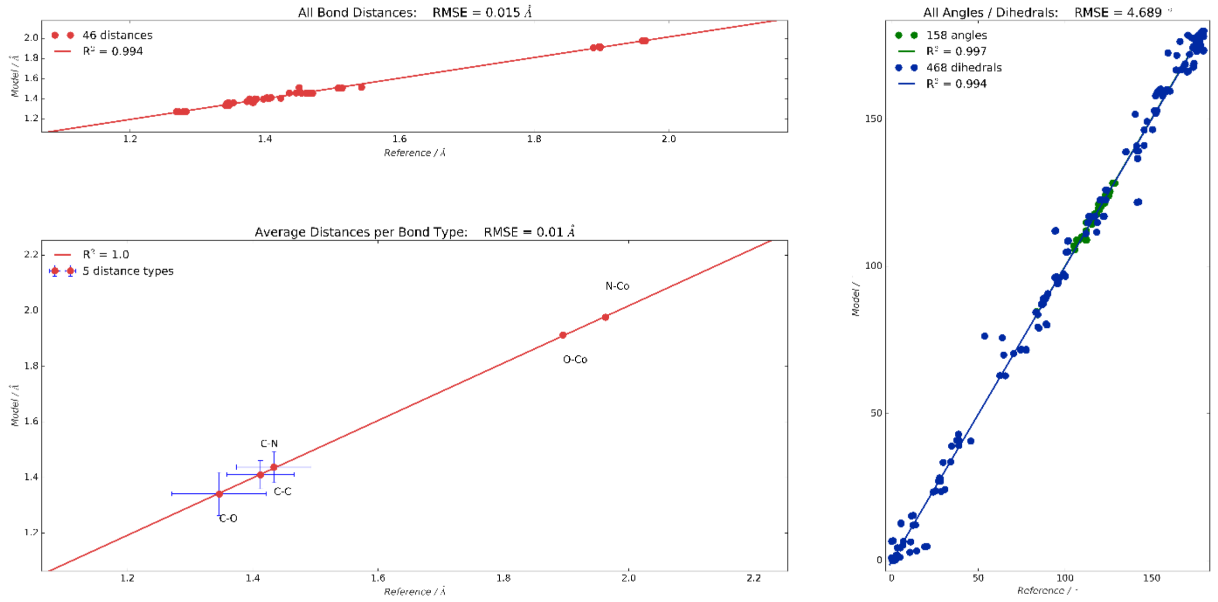

Figure S62: Detailed analysis of the Root-Mean-Square-Error (RMSE) for bonds and angles for the experimentally determined structure of [Co(acac)$_2$(L1)](PF$_6$) (Model) and the calculated structure (Reference).

The crystal structure of [Co(acac)$_2$(L1)](SbF$_6$)$_2$ is in good agreement with that calculated, with a total RMSD of 0.16 Å. All bond distances are in excellent agreement, as evinced with the RMSE of 0.012 Å (Figure S64).

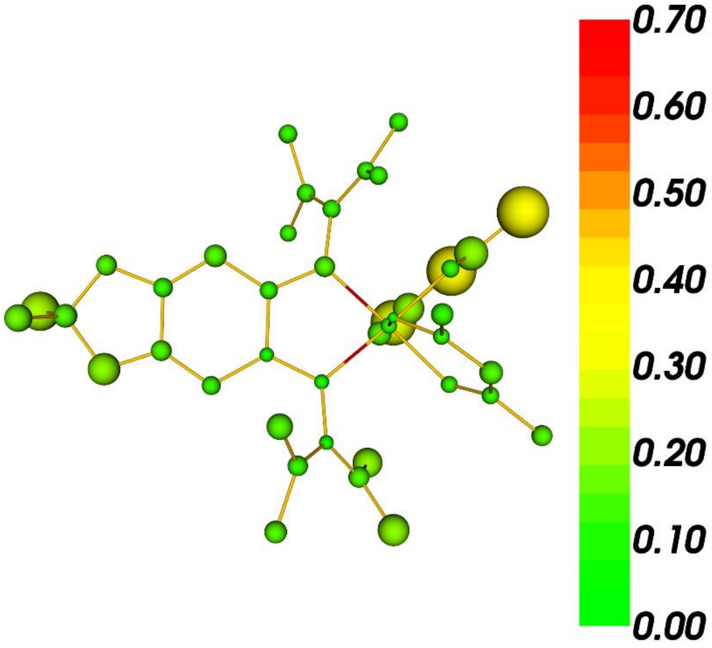

Figure S63: Superposition with Root-Mean-Square-Deviation (RMSD) for the experimentally determined and calculated structures of [Co(acac)$_2$(L1)](SbF$_6$)$_2$. The sphere dimensions reflect the relative RMSD distribution and the color code the absolute deviation (small for green color and large for red color).

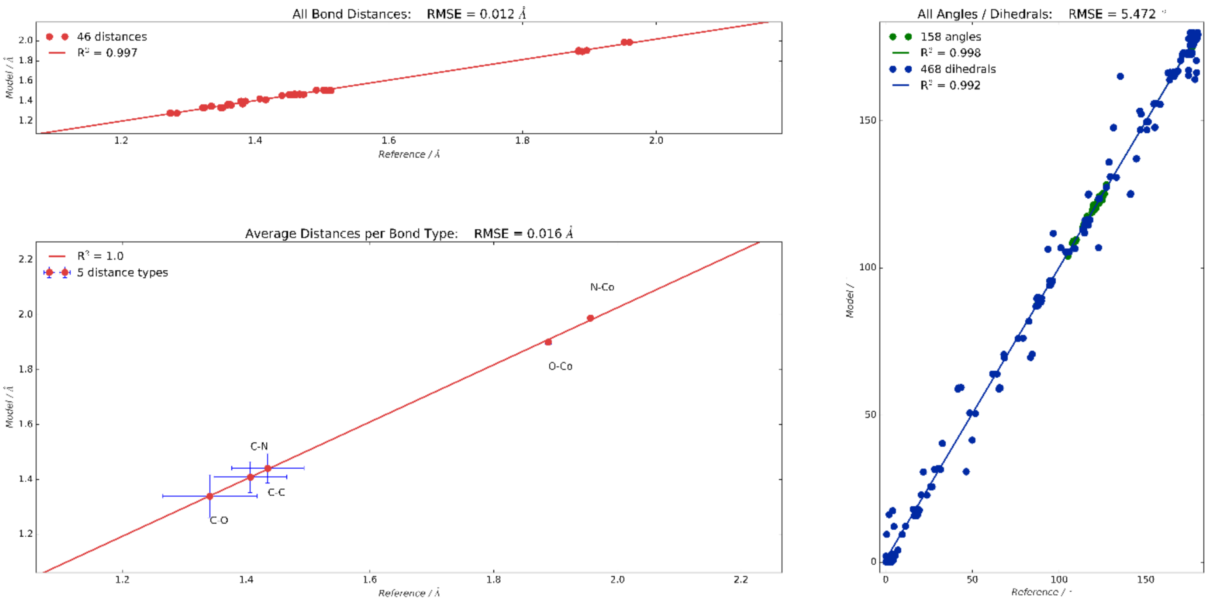

Figure S64: Detailed analysis of the Root-Mean-Square-Error (RMSE) for bonds and angles for the experimentally determined structure of $[Co(acac)_2(L1)](SbF_6)_2$ (Model) and the calculated structure (Reference).

The crystal structure of $[Co(acac)_2(L3)](PF_6)_2$ is in good agreement with that calculated, with a total RMSD of 0.47 Å. All bond distances are in excellent agreement, as evinced with the RMSE of 0.04 Å (Figure S66).

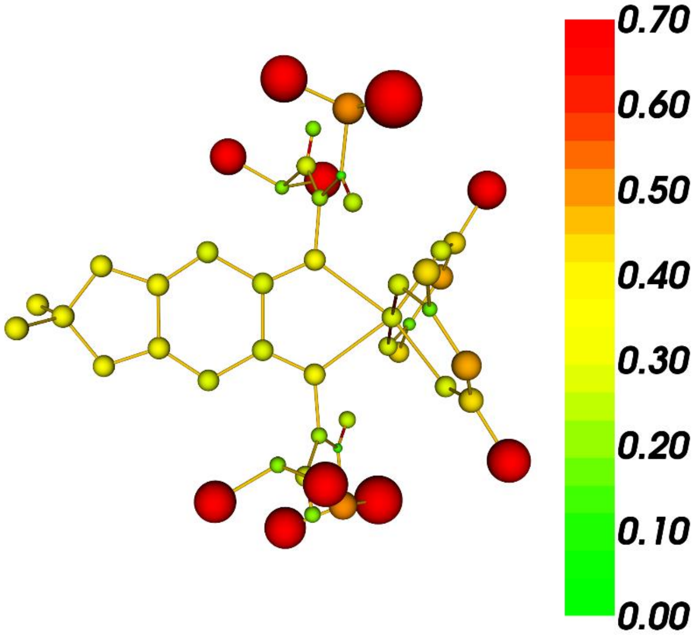

Figure S65: Superposition with Root-Mean-Square-Deviation (RMSD) for the experimentally determined and calculated structures of $[Co(acac)_2(L3)](PF_6)_2$. The sphere dimensions reflect the relative RMSD distribution and the color code the absolute deviation (small for green color and large for red color).

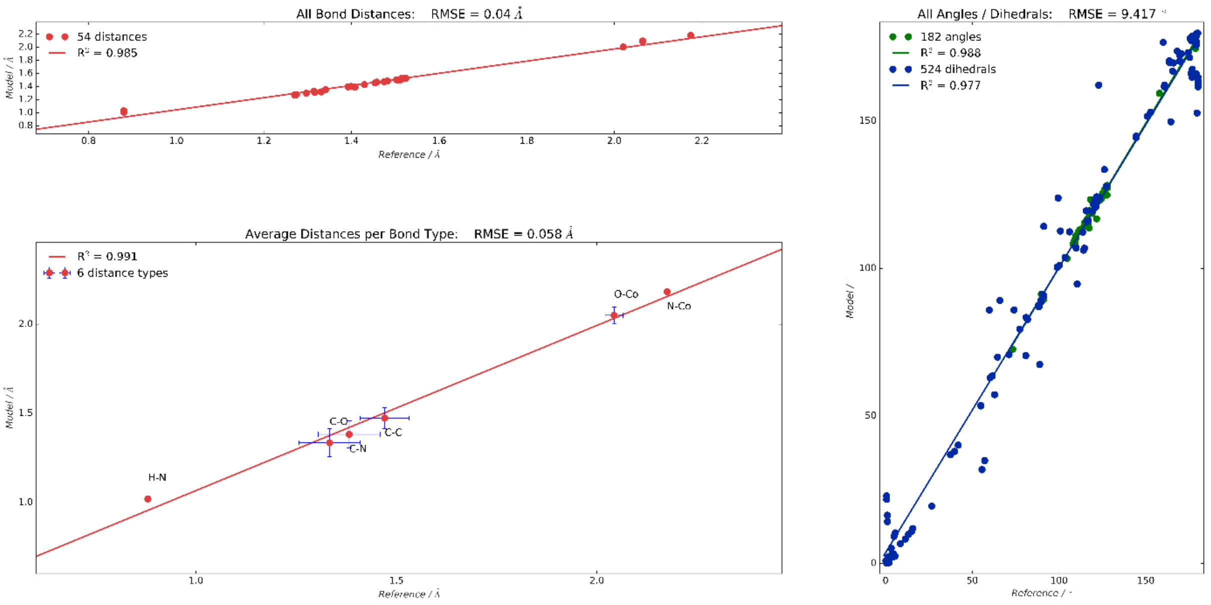

Figure S66: Detailed analysis of the Root-Mean-Square-Error (RMSE) for bonds and angles for the experimentally determined structure of [Co(acac)$_2$(L3)](PF$_6$)$_2$ (Model) and the calculated structure (Reference).

5.4 Cartesian coordinates for all complexes, calculated with B3LYP/def2-TZVP

1) [Co(acac)$_2$(L1)]

88

Energy = -3295.181662114

Co	6.5251836	4.7110666	15.0964951
O	2.5008654	3.6004101	9.8397306
O	0.9115153	3.9227819	11.4963706
O	6.8474174	2.7513545	15.6412289
O	6.7455666	5.1941762	17.1253686
O	8.6230258	5.0468916	14.9815572
O	6.3787135	6.7945671	14.7177398
N	4.3607882	4.6865282	14.9287272
N	3.7071165	4.9361941	17.1280571
N	2.8694778	6.4284160	15.5556834
N	6.2188499	4.3204616	12.9904611
N	7.0391360	5.6109379	11.1745857
N	8.3757892	4.0205588	12.2165651
C	3.9031607	4.4938252	13.6205288
C	4.8712264	4.2996134	12.6112954
C	4.4649087	3.9704569	11.2995578
H	5.1939576	3.7746356	10.5262084
C	3.1212409	3.8806775	11.0343933
C	2.1727674	4.0728438	12.0230992
C	2.5306487	4.3617014	13.3161757
H	1.7802188	4.4671108	14.0867804
C	3.6583656	5.3360190	15.8267463
C	4.0498853	3.5629942	17.4647213
H	3.9025700	2.9272644	16.5968691
H	3.4033616	3.2296806	18.2823885
H	5.0904490	3.4733875	17.7754764
C	3.7923665	5.8837336	18.2267722
H	3.7067889	6.9003531	17.8534135
H	4.7668529	5.7836076	18.7100350
H	3.0038433	5.7054427	18.9647275
C	3.1601755	7.3043864	14.4348457
H	4.2075558	7.2059030	14.1648069
H	2.9755387	8.3377916	14.7423301
H	2.5305137	7.0794691	13.5672419
C	1.5805463	6.6327011	16.1906467
H	1.3797154	5.8358563	16.9022444
H	0.7891281	6.6290139	15.4317576
H	1.5388831	7.5918790	16.7172276
C	7.1689139	4.6402577	12.1417231
C	6.1330929	6.7295134	11.3637284
H	5.1856129	6.5830076	10.8339113
H	6.6112965	7.6359755	10.9814300
H	5.9342162	6.8599491	12.4241295
C	7.5562180	5.4434593	9.8292577
H	8.0808347	4.4953104	9.7419703
H	8.2441846	6.2510566	9.5584318
H	6.7282215	5.4476810	9.1101835
C	8.5124396	2.7444181	12.9043225

H	8.8932270	2.8957463	13.9149418
H	9.2043558	2.1128385	12.3393558
H	7.5435482	2.2615966	12.9821189
C	9.6259420	4.7260631	11.9864813
H	9.4352711	5.7750587	11.7768620
H	10.1847976	4.2889311	11.1528760
H	10.2356592	4.6637198	12.8895053
C	1.0957827	3.4178206	10.1493716
C	0.7750615	1.9332315	10.1305926
H	1.4158872	1.4166187	10.8455073
H	0.9520429	1.5250091	9.1351908
H	-0.2673223	1.7724456	10.4069126
C	0.2691707	4.2489664	9.1944584
H	-0.7888833	4.1549561	9.4399245
H	0.4291456	3.9084769	8.1712058
H	0.5638069	5.2945992	9.2742018
C	7.5344034	2.3940671	16.6387637
C	7.8990559	3.2091636	17.7262063
H	8.5138433	2.7660330	18.4964986
C	7.4924727	4.5432072	17.9066242
C	7.9862076	0.9485871	16.6291923
H	7.1131043	0.3043920	16.5069811
H	8.5264423	0.6607829	17.5304479
H	8.6282561	0.7872910	15.7596623
C	7.9519631	5.2878009	19.1429402
H	8.4870686	6.1856571	18.8254027
H	8.5940589	4.6916876	19.7904371
H	7.0795674	5.6195656	19.7111691
C	9.1910281	6.1191486	15.3169564
C	8.5847924	7.3888194	15.3698613
H	9.2039127	8.2261845	15.6586360
C	7.2443530	7.6530283	15.0537064
C	10.6538729	5.9981580	15.6958514
H	10.7291998	5.3324503	16.5590358
H	11.1168250	6.9545031	15.9367221
H	11.2063787	5.5266723	14.8804523
C	6.7522462	9.0855354	15.1015663
H	6.3290276	9.3519179	14.1301076
H	7.5327939	9.8000453	15.3603459
H	5.9452394	9.1542428	15.8349777

2) [Co(acac)$_2$(L2)]

84
Energy = -3292.787175053

Co	1.4675471	9.8528718	8.0788108
O	0.9249189	11.7435506	1.6742028
O	2.8998059	10.5345607	1.5727703
O	2.5883732	11.6477480	8.1608002
O	0.3137130	10.6659636	9.6433989
O	2.8063089	9.0841377	9.5035166
O	0.3652163	8.1130746	8.3906702
N	2.6038619	9.2812908	6.3259198
N	5.0111822	9.5884047	6.2961100
N	4.1252155	7.7766086	7.2386269
N	0.3092265	10.7158835	6.4559199
N	-0.4530898	13.0100321	6.5094936
N	-1.6004539	11.4391181	7.5860581
C	2.2167698	9.9822888	5.1823794
C	1.0138685	10.7243619	5.2450812
C	0.5013509	11.3330908	4.0788331
H	-0.4287194	11.8825799	4.1067301
C	1.2101319	11.2091152	2.9101677
C	2.3885121	10.4877664	2.8494644
C	2.9044025	9.8582899	3.9547061
H	3.8151117	9.2795394	3.8909902
C	3.8362821	8.9208783	6.5814816
C	6.0916718	8.9940735	7.0877923
H	6.2597464	9.5947091	7.9898247
H	7.0214224	8.9547980	6.5189196
C	5.5468298	7.6097420	7.4491237
H	5.9398936	6.8242565	6.7907661
H	5.7662056	7.3292047	8.4819401
C	5.0494804	11.0388913	6.1662674
H	4.3062535	11.3764971	5.4511388
H	6.0373251	11.3212399	5.7997465
H	4.8487359	11.5244903	7.1245075
C	3.2001034	6.6917110	7.4531661
H	2.2023698	7.0198407	7.1772180
H	3.1864407	6.4107435	8.5071818
H	3.4875610	5.8205848	6.8527647
C	-0.5180436	11.6673108	6.8064838
C	-1.4339550	13.7437271	7.3060765
H	-1.9484133	14.5006497	6.7116613
H	-0.9256868	14.2414491	8.1404023
C	-2.3731753	12.6425700	7.8117162
H	-2.6259769	12.7636703	8.8675924
H	-3.3104565	12.6033047	7.2421015
C	0.7906188	13.6842723	6.1785712
H	1.0260005	14.4141216	6.9582587
H	0.7131668	14.1993136	5.2185697
H	1.6042910	12.9680156	6.1321164
C	-2.1347505	10.1365071	7.9025304
H	-3.1175378	10.0053927	7.4347741
H	-2.2269973	10.0275500	8.9842981

H	-1.4486682	9.3711417	7.5518245
C	1.8739718	11.1492271	0.7536368
C	1.1715023	10.0689423	-0.0514603
H	0.7520170	9.3277873	0.6292370
H	1.8812259	9.5778005	-0.7177765
H	0.3653571	10.5063528	-0.6411402
C	2.5006213	12.2428549	-0.0815455
H	1.7345512	12.7431846	-0.6743576
H	3.2480635	11.8170922	-0.7513099
H	2.9791180	12.9702837	0.5729794
C	2.4176618	12.6292048	8.9329749
C	1.3943137	12.7692230	9.8862595
H	1.3861934	13.6755598	10.4753521
C	0.4390120	11.7820264	10.2043002
C	3.4390960	13.7441068	8.8046466
H	3.1783388	14.6334289	9.3778381
H	3.5544205	14.0094436	7.7521025
H	4.4066076	13.3739399	9.1533122
C	-0.5341607	12.0571068	11.3356161
H	-0.4580901	13.0684786	11.7341080
H	-0.3461429	11.3414276	12.1392467
H	-1.5525393	11.8797451	10.9836886
C	2.4822625	8.3010638	10.4357598
C	1.3173895	7.5134825	10.4829851
H	1.1694015	6.8894080	11.3525089
C	0.3464199	7.4563172	9.4683349
C	3.4694170	8.2107931	11.5826894
H	3.1644730	7.5100397	12.3593380
H	3.5908087	9.2045927	12.0192138
H	4.4445726	7.9112161	11.1908427
C	-0.8351141	6.5250398	9.6412715
H	-0.9186251	5.8856373	8.7601311
H	-1.7473612	7.1249183	9.6919452
H	-0.7670507	5.9062448	10.5354297

3) [Co(acac)$_2$(L3)]

100
Energy = -3452.461705414

Co	-1.2493739	0.5680477	-0.0796458
O	5.1428203	1.1426158	-1.3548334
O	5.1527772	1.4621696	0.9436187
O	-2.8062287	1.0244960	1.2768271
O	-1.8942171	-1.4418275	0.1351115
O	-2.6449975	0.9881342	-1.6059805
O	-0.7911276	2.5829830	-0.0968137
N	0.3914597	0.2528226	1.3143837
N	-0.8196036	0.2628038	3.2540520
H	-1.4607039	0.8151586	2.6910861
N	1.1909091	-0.9236560	3.2193691
H	0.9705918	-1.0974666	4.1866154
N	0.3635463	-0.0487008	-1.3463893
N	1.1110799	-1.6962315	-2.8778921
H	0.9188301	-2.0613467	-3.7979348
N	-0.8500897	-0.4756322	-3.2416947
H	-1.4393915	0.2547244	-2.8436673
C	1.6139177	0.3997911	0.6485191
C	1.6048440	0.2211131	-0.7505095
C	2.7750113	0.4519538	-1.5043638
H	2.7705957	0.3396252	-2.5781511
C	3.9071689	0.8408145	-0.8353208
C	3.9132674	1.0316912	0.5371481
C	2.7895985	0.8345204	1.2980960
H	2.7974660	1.0080535	2.3640453
C	0.2725687	-0.1451401	2.5523958
C	-1.4381486	-0.4335122	4.3829336
H	-0.6620467	-0.6464676	5.1261385
C	-2.1050621	-1.7491778	3.9724587
H	-2.9209246	-1.5560895	3.2747042
H	-2.5073842	-2.2652334	4.8476264
H	-1.3941967	-2.4103294	3.4785240
C	-2.4349792	0.5208099	5.0374208
H	-1.9432573	1.4415295	5.3540042
H	-2.8967676	0.0513815	5.9071159
H	-3.2273430	0.7812416	4.3327694
C	1.9196834	-2.0258761	2.5743106
H	2.4637261	-1.6068474	1.7291230
C	2.9367876	-2.5714465	3.5696200
H	3.6283229	-1.7900755	3.8886016
H	3.5132538	-3.3795713	3.1174362
H	2.4387189	-2.9776671	4.4562509
C	0.9770695	-3.1102525	2.0506582
H	0.4741968	-3.6210106	2.8762759
H	1.5389591	-3.8582435	1.4877473
H	0.2208575	-2.6809100	1.3915481
C	0.2180619	-0.7441934	-2.4444941
C	1.6902253	-2.6894097	-1.9616862
H	2.0481752	-2.1465293	-1.0874155
C	2.8852970	-3.3438025	-2.6443615

H	2.5768112	-3.8698498	-3.5536479
H	3.3489520	-4.0762953	-1.9818475
H	3.6326878	-2.5967123	-2.9145526
C	0.6377243	-3.7053484	-1.5126430
H	-0.1806459	-3.2005990	-0.9968413
H	1.0728796	-4.4383680	-0.8305990
H	0.2320862	-4.2449155	-2.3735578
C	-1.6162893	-1.4559389	-4.0180801
H	-0.9198375	-2.1734797	-4.4596140
C	-2.6021834	-2.2120814	-3.1256622
H	-3.1786452	-2.9355159	-3.7075466
H	-3.2862483	-1.5061789	-2.6510622
H	-2.0710315	-2.7365137	-2.3337185
C	-2.3238544	-0.7267461	-5.1582382
H	-2.8891704	-1.4305964	-5.7715874
H	-1.6066156	-0.2037049	-5.7922693
H	-3.0252883	0.0080787	-4.7567159
C	6.0099453	1.4114832	-0.2246743
C	6.6629573	2.7629114	-0.4153626
H	7.2801058	2.7545055	-1.3141566
H	7.2885286	2.9988807	0.4458511
H	5.8922494	3.5258694	-0.5175868
C	6.9862699	0.2588824	-0.0684967
H	6.4298712	-0.6695252	0.0626779
H	7.6193672	0.4204276	0.8044152
H	7.6120347	0.1757732	-0.9574909
C	-3.9074973	0.3997290	1.2940298
C	-4.0855363	-0.9377489	0.9146747
H	-5.0655268	-1.3711371	1.0581419
C	-3.0674046	-1.7893252	0.4404105
C	-5.0964585	1.1884340	1.7956156
H	-4.8618713	1.6256321	2.7684170
H	-6.0061224	0.5939895	1.8731713
H	-5.2657312	2.0206987	1.1079125
C	-3.3952164	-3.2604206	0.2730820
H	-3.6679894	-3.4535452	-0.7672867
H	-4.2230390	-3.5788366	0.9063271
H	-2.5089713	-3.8539978	0.4985221
C	-3.2089620	2.1219498	-1.7446347
C	-2.7559589	3.3293133	-1.2125897
H	-3.3338231	4.2159622	-1.4310370
C	-1.5703317	3.4985280	-0.4587466
C	-4.4712395	2.1116258	-2.5789651
H	-4.2351629	1.7619308	-3.5870660
H	-4.9473951	3.0890059	-2.6469572
H	-5.1720323	1.3942070	-2.1459090
C	-1.1758158	4.8986223	-0.0374325
H	-1.1786466	4.9466564	1.0540650
H	-1.8365692	5.6695698	-0.4324797
H	-0.1526357	5.0911743	-0.3655238

4) $[Co(III)(acac)_2(L1)]^+$

88
Energy = -3294.997794527

Co	7.5509298	5.3672607	7.2266436
O	5.1387241	11.2412969	8.6270549
O	4.7011148	10.1266044	10.6186465
O	7.8674353	3.5637275	7.7927832
O	9.2636324	5.9934678	7.7891184
O	8.3846513	4.8099555	5.5924767
O	5.8073358	4.8042213	6.6944242
N	6.7917649	5.9916448	8.9412203
N	6.6852417	4.1352188	10.3290012
N	7.6899882	6.0719288	11.1246902
N	7.1124685	7.1970009	6.6193627
N	8.0302762	7.4237319	4.5004433
N	5.7997446	7.9847730	4.8196896
C	6.3431864	7.3210288	8.8677278
C	6.5242496	7.9650877	7.6381664
C	6.1813491	9.3255926	7.4881002
H	6.3719912	9.8595196	6.5690614
C	5.5852722	9.9564500	8.5512612
C	5.3270248	9.2911187	9.7433333
C	5.6894038	7.9806141	9.9300107
H	5.4791453	7.4796735	10.8633143
C	7.0539766	5.4166503	10.1140230
C	5.5400718	3.5468847	9.6442664
H	5.0069728	4.3174180	9.0974797
H	4.8824407	3.0861462	10.3861850
H	5.8680185	2.7924234	8.9297943
C	7.4567858	3.2223486	11.1590487
H	8.4220937	3.6587137	11.4013625
H	7.6244202	2.2984271	10.6035021
H	6.9329627	2.9702764	12.0848158
C	8.6133586	7.1661845	10.8598387
H	8.8964517	7.1680183	9.8123268
H	9.5052302	7.0288432	11.4758621
H	8.1613873	8.1307361	11.1065037
C	7.3116311	5.9114512	12.5235722
H	7.0572246	6.8910531	12.9383032
H	8.1276943	5.4908039	13.1163344
H	6.4414722	5.2666613	12.6108012
C	6.9753970	7.5316323	5.3370259
C	9.4009698	7.5426533	4.9833239
H	9.3959844	7.8655674	6.0189652
H	9.9325802	8.2694669	4.3634891
H	9.9090222	6.5799486	4.9328576
C	7.8885750	7.0653864	3.0971608
H	8.5765746	6.2492162	2.8716625
H	8.1276751	7.9021188	2.4356347
H	6.8754754	6.7277951	2.8954309
C	5.7387537	9.0525330	3.8287455
H	6.7312625	9.4511645	3.6375930

H	5.1112423	9.8609364	4.2147294
H	5.3069746	8.7047668	2.8870026
C	4.5177426	7.6132296	5.4018539
H	4.6447509	6.7698914	6.0726654
H	3.8342629	7.3353742	4.5959397
H	4.0825599	8.4470198	5.9593794
C	4.6192553	11.4328784	9.9759703
C	3.1690401	11.8492180	9.8905555
H	2.7535854	11.9535873	10.8927155
H	2.6016878	11.0949474	9.3462367
H	3.0872296	12.8033914	9.3703113
C	5.5167922	12.4019931	10.7153067
H	5.5034266	13.3727972	10.2201418
H	6.5383627	12.0218419	10.7242629
H	5.1707784	12.5196267	11.7421089
C	9.0133378	3.0962640	8.0758638
C	10.1764822	3.8512414	8.2554307
H	11.0829362	3.3281925	8.5196242
C	10.2336690	5.2402909	8.1206070
C	9.0801432	1.5955703	8.1948226
H	9.3215345	1.1823896	7.2113141
H	8.1121552	1.1942978	8.4901790
H	9.8509409	1.2716870	8.8927861
C	11.5260590	5.9687763	8.3758071
H	11.8501302	6.4480247	7.4493381
H	12.3148197	5.3091717	8.7310811
H	11.3586868	6.7623818	9.1060145
C	7.8456806	4.0289417	4.7489039
C	6.5011270	3.6452380	4.7411657
H	6.1663742	2.9925984	3.9492630
C	5.5651809	4.0608962	5.6909188
C	8.7778257	3.4760394	3.7020169
H	9.1890049	2.5319481	4.0706602
H	9.6122464	4.1558345	3.5388007
H	8.2668551	3.2739737	2.7616182
C	4.1290003	3.6255746	5.5732204
H	3.4790513	4.5016835	5.6037269
H	3.8756689	3.0076668	6.4375950
H	3.9360985	3.0617488	4.6631001

5) $[Co(III)(acac)_2(L2)]^+$

84
Energy = -3292.600949450

Co	1.4607771	9.9942567	8.1246360
O	0.8660465	11.4953685	1.8248753
O	2.8224637	10.2476099	1.7722231
O	2.5473230	11.5775237	8.2069084
O	0.2930879	10.5719628	9.5216343
O	2.5900949	9.2771298	9.4864575
O	0.3952113	8.4221260	8.0175327
N	2.6224486	9.4278569	6.6267977
N	4.9840514	9.8847705	6.5447155
N	4.2998426	7.9796232	7.4154561

N	0.4063706	10.8551397	6.6927524
N	-0.3216820	13.1430968	6.6510795
N	-1.6201316	11.6353625	7.6040201
C	2.2264001	10.0180464	5.4099809
C	1.0500611	10.7736390	5.4421052
C	0.5001165	11.2945845	4.2539734
H	-0.4322917	11.8411773	4.2552945
C	1.1814072	11.0687776	3.0824199
C	2.3510663	10.3222434	3.0507500
C	2.8846927	9.7689287	4.1895204
H	3.7708442	9.1514312	4.1436128
C	3.9021458	9.1317190	6.8446767
C	6.2202887	9.1439039	6.7859088
H	6.9494700	9.7753822	7.2927494
H	6.6581759	8.8015586	5.8424033
C	5.7363441	7.9768090	7.6629639
H	6.1729423	7.0220741	7.3703858
H	5.9442015	8.1419561	8.7249765
C	4.9884142	11.2496394	6.0677855
H	4.0266975	11.7041085	6.2793350
H	5.1845977	11.2976870	4.9931977
H	5.7629901	11.8073101	6.5973445
C	3.4675709	6.8257748	7.6548869
H	2.4637608	7.0455120	7.3019051
H	3.4234453	6.5858585	8.7178738
H	3.8657248	5.9610347	7.1174998
C	-0.4544945	11.8308128	6.9600461
C	-1.5356349	13.8848830	6.9837427
H	-2.1076051	14.1216744	6.0809810
H	-1.2846711	14.8191907	7.4854796
C	-2.2791135	12.9008097	7.9038797
H	-2.1637538	13.1569562	8.9623592
H	-3.3438677	12.8389607	7.6805550
C	0.8768540	13.7955186	6.1749693
H	1.0462257	14.7031578	6.7582743
H	0.7981977	14.0608472	5.1175090
H	1.7237968	13.1287316	6.3060387
C	-2.2050298	10.3538242	7.9280257
H	-3.2294564	10.3141873	7.5504840
H	-2.2074101	10.1888759	9.0064766
H	-1.6051950	9.5732607	7.4680906
C	1.8070774	10.8503919	0.9183266
C	1.0875205	9.7541481	0.1589726
H	0.6541448	9.0443680	0.8638692
H	1.7886434	9.2283875	-0.4890682
H	0.2907506	10.1834989	-0.4482899
C	2.4546576	11.9024606	0.0499303
H	1.6988211	12.3937911	-0.5625281
H	3.1944895	11.4398706	-0.6031054
H	2.9456647	12.6456713	0.6769981
C	2.4878845	12.4515005	9.1267165
C	1.5346142	12.4937756	10.1486312
H	1.5986126	13.2931641	10.8705530
C	0.4983929	11.5660123	10.2848453
C	3.5593966	13.5074526	9.0571393
H	3.4127231	14.3013449	9.7862728

H	3.5880784	13.9352543	8.0537836
H	4.5279619	13.0351094	9.2360359
C	-0.4938075	11.7015381	11.4091321
H	-0.3335505	12.5982547	12.0038976
H	-0.4169265	10.8229837	12.0533382
H	-1.5071070	11.7104668	11.0034846
C	2.2046539	8.3704224	10.2945389
C	1.0676704	7.5775891	10.1326971
H	0.8497024	6.8373378	10.8868007
C	0.2644067	7.6093045	8.9861033
C	3.1151422	8.1610958	11.4738224
H	2.7684628	7.3687892	12.1336900
H	3.1921844	9.0950832	12.0339984
H	4.1184938	7.9192227	11.1163776
C	-0.8451856	6.6079159	8.8148669
H	-0.7622668	6.1390807	7.8332790
H	-1.8018801	7.1354671	8.8401111
H	-0.8415147	5.8434772	9.5888540

6) $[Co(III)(acac)_2(L3)]^+$

100
Energy = -3452.276000388

Co	23.0922806	4.8440936	11.6988475
O	29.0302403	3.3945123	10.1727691
O	29.3918242	4.8651883	11.9302088
O	22.0889544	6.3866720	12.2722174
O	22.1465886	3.6511864	12.8661430
O	21.7028255	4.6184795	10.3851292
O	24.0895580	6.0397663	10.6136357
N	24.5778311	4.9725948	13.0003999
N	23.6801580	6.3186538	14.6327907
H	23.1904902	6.7394116	13.8539513
N	25.3855870	4.8600243	15.2144369
H	25.4034266	5.3620615	16.0880623
N	24.1808847	3.3240102	11.0765142
N	24.5134002	0.9970911	10.8721924
H	24.1692197	0.1855121	10.3849708
N	22.5825535	2.0110405	10.0637458
H	22.0974351	2.8979627	9.9655628
C	25.7540965	4.5212420	12.3643257
C	25.5380488	3.6345550	11.3074163
C	26.5943877	3.2147033	10.4804707
H	26.4255734	2.5499202	9.6465733
C	27.8497667	3.6784236	10.7906136
C	28.0670698	4.5607704	11.8453494
C	27.0411286	5.0185996	12.6356578
H	27.2159196	5.7359411	13.4229041
C	24.5463410	5.3542585	14.2677431
C	23.1854955	6.6097644	15.9831835
H	24.0456568	6.7131489	16.6526615
C	22.2718364	5.5036383	16.5139624
H	21.3790362	5.4290074	15.8913125
H	21.9639271	5.7230415	17.5376733

H	22.7771665	4.5384160	16.5099211
C	22.4756804	7.9600978	15.9487048
H	23.1341154	8.7429360	15.5701328
H	22.1489003	8.2385034	16.9503481
H	21.5897370	7.9109095	15.3116559
C	25.8639997	3.4701147	15.3018820
H	25.8538281	3.0687651	14.2909086
C	27.2967825	3.4575431	15.8243665
H	27.9580404	4.0194017	15.1656155
H	27.6661888	2.4334940	15.8915689
H	27.3497293	3.8939313	16.8257941
C	24.9232923	2.6400009	16.1759741
H	24.8602716	3.0570525	17.1844786
H	25.2870237	1.6153617	16.2643533
H	23.9207341	2.6088081	15.7475484
C	23.7655898	2.1249215	10.7001732
C	25.2714592	0.6767376	12.0962029
H	25.9580118	1.5005077	12.2836944
C	26.0930815	-0.5779609	11.8297760
H	25.4459387	-1.4316592	11.6057937
H	26.6827134	-0.8358274	12.7096859
H	26.7743205	-0.4288349	10.9911101
C	24.3441137	0.5272249	13.3018183
H	23.7463537	1.4294039	13.4434819
H	24.9270618	0.3537302	14.2073893
H	23.6703469	-0.3231127	13.1709017
C	21.7253389	0.8227418	9.9905471
H	22.3500599	-0.0481978	9.7777995
C	20.9735152	0.5877404	11.3010191
H	20.3641155	-0.3156440	11.2395513
H	20.3168020	1.4339630	11.5098318
H	21.6676921	0.4826859	12.1338228
C	20.7685620	0.9967590	8.8134629
H	20.1434430	0.1110403	8.6989169
H	21.3151347	1.1592487	7.8838417
H	20.1073230	1.8503482	8.9827436
C	30.0409874	4.2523517	10.7776105
C	30.4207972	5.3377776	9.7917210
H	30.8620016	4.8921632	8.9004092
H	31.1404559	6.0183023	10.2465214
H	29.5317364	5.9000853	9.5054446
C	31.1909085	3.3983753	11.2557061
H	30.8259520	2.6491493	11.9572472
H	31.9339623	4.0220241	11.7523806
H	31.6574839	2.8973036	10.4077991
C	20.9009689	6.2976504	12.7414502
C	20.3369170	5.1404043	13.2677844
H	19.3458145	5.2038232	13.6912039
C	21.0201773	3.9181496	13.3826058
C	20.1224902	7.5831373	12.7170055
H	20.7513315	8.4065102	13.0577374
H	19.2151877	7.5361134	13.3159588
H	19.8521145	7.7998772	11.6801152
C	20.4233217	2.8300487	14.2352485
H	20.8262784	1.8611937	13.9520494
H	19.3357517	2.8176089	14.1672898

H	20.6869804	3.0195185	15.2796382
C	21.5351530	5.3926010	9.3731309
C	22.4152762	6.3835112	8.9669657
H	22.1616175	6.9623183	8.0925386
C	23.6588560	6.6219122	9.5776592
C	20.2799275	5.1206138	8.5914313
H	20.3593749	4.1416426	8.1121279
H	20.1006741	5.8696500	7.8231616
H	19.4295021	5.0809225	9.2740094
C	24.6031589	7.6358875	8.9929721
H	24.7270937	8.4524405	9.7078715
H	24.2499976	8.0378776	8.0458433
H	25.5821004	7.1741977	8.8561900

7) $[Co(III)(acac)_2(L1^{\cdot +})]^{2+}$

88

Energy = -3294.706755596

Co	2.9633703	7.2913771	10.3937121
O	1.2907123	8.1912032	10.5420235
O	4.6680846	6.4575184	10.2248628
O	2.0357102	5.8965944	9.5080906
O	2.7337985	6.3012877	11.9934825
O	5.8794223	13.0400056	9.8481964
O	5.3874936	12.6128374	7.6281192
N	3.8231536	8.8621292	11.2584045
N	3.0927939	10.2019417	13.0585841
N	4.2901066	8.2475838	13.4547558
N	3.3692448	8.3305037	8.7483045
N	4.4301204	7.6677220	6.7477159
N	2.1400648	7.3651240	7.0227615
C	4.2385431	9.8152920	10.3833277
C	3.9854613	9.5185321	8.9832713
C	4.3321315	10.4526960	7.9831748
H	4.1109349	10.2796326	6.9413104
C	4.9586979	11.6018498	8.3825993
C	5.2566173	11.8665047	9.7470799
C	4.9128621	11.0003739	10.7493514
H	5.1574875	11.2222107	11.7766462
C	3.7419767	9.1139153	12.6025060
C	2.0608874	10.8852122	12.2796498
H	2.4347766	11.8417014	11.9071400
H	1.2060808	11.0757399	12.9302287
H	1.7440741	10.2678171	11.4467482
C	3.4710403	10.8892034	14.2967791
H	2.7298090	10.7465191	15.0842899
H	3.5402305	11.9570992	14.0823759
H	4.4404287	10.5442452	14.6443724
C	5.4023422	7.3791721	13.0654830
H	5.0503929	6.3541258	12.9633629
H	6.1685795	7.4330528	13.8402975
H	5.8134265	7.6975023	12.1146483
C	3.7360355	7.9769124	14.7820256
H	4.3557422	8.3936075	15.5768778
H	3.6960406	6.8958108	14.9102841

H	2.7267053	8.3709536	14.8586595
C	3.3134245	7.7922323	7.4897094
C	5.7657900	7.6163739	7.3409841
H	6.3032553	8.5485093	7.1521251
H	6.3180097	6.7969407	6.8785126
H	5.6993186	7.4431325	8.4090606
C	4.4203652	7.7342947	5.2834011
H	4.6241092	6.7629984	4.8305940
H	5.2037683	8.4274139	4.9720813
H	3.4663682	8.1088409	4.9242275
C	2.0109888	6.2221432	6.1178148
H	1.7427629	6.5293201	5.1063035
H	1.2176302	5.5826286	6.5029582
H	2.9344434	5.6506427	6.0952881
C	0.8708469	7.9110669	7.5064883
H	0.3687461	7.1755520	8.1325914
H	0.2479143	8.1506802	6.6433739
H	1.0453282	8.8028147	8.0969933
C	6.0115008	13.6133441	8.5029825
C	5.2182412	14.8940508	8.4319736
H	5.2646590	15.2959307	7.4205906
H	5.6404853	15.6233739	9.1221819
H	4.1772908	14.7096393	8.6962706
C	7.4748907	13.7278425	8.1569815
H	7.5808140	14.0980110	7.1379656
H	7.9602004	12.7556487	8.2385458
H	7.9572535	14.4268069	8.8391802
C	1.6127538	6.1294347	12.5782212
C	0.4367509	6.8236209	12.2847480
H	-0.4446490	6.5891936	12.8619339
C	0.3405367	7.8165891	11.3106647
C	1.6058916	5.0754783	13.6504170
H	1.2208854	4.1461844	13.2206124
H	0.9560963	5.3456638	14.4819639
H	2.6156049	4.8819612	14.0065703
C	-0.9577504	8.5424169	11.0967690
H	-1.3884072	8.2182227	10.1457158
H	-0.7770399	9.6151834	11.0179975
H	-1.6783779	8.3487940	11.8879735
C	4.8372982	5.2231476	9.9393803
C	3.8338954	4.3473136	9.5265235
H	4.1031779	3.3210666	9.3285573
C	2.5032405	4.7228848	9.3285620
C	6.2527450	4.7390493	10.0805984
H	6.9379522	5.4496807	9.6167141
H	6.4004236	3.7517803	9.6490695
H	6.5046857	4.6967449	11.1435005
C	1.4988655	3.6965825	8.8834680
H	1.1477559	3.1513934	9.7644313
H	1.9351441	2.9696549	8.1996177
H	0.6356009	4.1757095	8.4262009

8) [*is*-Co(III)(acac)$_2$(L1$^{\cdot+}$)]$^{2+}$

88
Energy = -3294.682347603

Co	2.9677363	7.2671377	10.4486331
O	1.3062761	8.0876918	10.7502611
O	4.6621175	6.4758080	10.2252511
O	2.0806256	5.8166130	9.5718122
O	2.7076101	6.1994974	12.2493196
O	6.0345481	13.0415377	9.8574863
O	5.5321181	12.6826146	7.6275228
N	3.8609232	8.9166663	11.2100965
N	3.0765274	10.2705462	12.9743769
N	4.2837901	8.3324036	13.4215141
N	3.3193319	8.4746543	8.6482137
N	4.3303181	7.7807905	6.6264224
N	2.0479228	7.4853735	6.9908446
C	4.2808929	9.8621372	10.3253522
C	4.0047847	9.6092472	8.9137182
C	4.3977677	10.5545281	7.9376783
H	4.1684653	10.4107014	6.8928445
C	5.0650356	11.6704480	8.3594147
C	5.3698281	11.8939612	9.7287279
C	4.9970395	11.0158206	10.7106041
H	5.2557989	11.2040085	11.7415385
C	3.7503563	9.1865186	12.5461021
C	2.0634041	10.9342110	12.1543189
H	2.4610519	11.8554128	11.7216253
H	1.2172997	11.1881884	12.7940164
H	1.7239416	10.2703016	11.3665673
C	3.4221205	10.9805656	14.2087439
H	2.6545200	10.8592133	14.9743908
H	3.5079156	12.0434760	13.9754117
H	4.3761688	10.6340300	14.5952135
C	5.4067536	7.4645885	13.0662474
H	5.0591005	6.4364884	12.9760160
H	6.1582112	7.5313835	13.8544990
H	5.8363918	7.7747620	12.1203730
C	3.6890594	8.0564885	14.7295243
H	4.2964693	8.4526260	15.5443340
H	3.6220807	6.9741710	14.8385605
H	2.6869779	8.4715732	14.7846792
C	3.2370049	7.9363883	7.4056460
C	5.6757009	7.6988114	7.1911228
H	6.2255731	8.6293547	7.0307169
H	6.2108586	6.8901810	6.6908278
H	5.6262756	7.4870202	8.2546018
C	4.2837540	7.8451818	5.1635576
H	4.4884768	6.8760232	4.7057824
H	5.0491639	8.5486741	4.8296405
H	3.3167601	8.2072302	4.8270835
C	1.8940969	6.3398846	6.0943312
H	1.5664933	6.6404937	5.0982137
H	1.1348364	5.6831730	6.5189905

H	2.8259672	5.7869167	6.0197601
C	0.8009710	7.9742830	7.5778519
H	0.3877862	7.2155652	8.2423345
H	0.0931210	8.1828634	6.7743659
H	0.9824573	8.8759291	8.1518694
C	6.1903879	13.6387131	8.5245889
C	5.4459909	14.9497450	8.4815644
H	5.5093682	15.3718971	7.4793966
H	5.8936331	15.6473308	9.1883750
H	4.3983037	14.7984634	8.7401812
C	7.6580448	13.7066337	8.1837913
H	7.7799701	14.0946843	7.1732364
H	8.1065841	12.7154503	8.2444330
H	8.1645462	14.3720499	8.8818459
C	1.5672269	5.8831228	12.6791868
C	0.3603171	6.4688021	12.2179525
H	-0.5631561	6.1224149	12.6581617
C	0.2930698	7.5345652	11.3409617
C	1.4923578	4.8604510	13.7810221
H	0.7256540	4.1140714	13.5656062
H	1.2054542	5.3438513	14.7189448
H	2.4556365	4.3720762	13.9105233
C	-1.0205653	8.1913662	11.0261097
H	-1.2393141	8.0698704	9.9628720
H	-0.9498534	9.2637055	11.2179888
H	-1.8407466	7.7752996	11.6064200
C	4.8651896	5.2176978	10.0443509
C	3.8806897	4.2872856	9.7345955
H	4.1720358	3.2562927	9.6073317
C	2.5488233	4.6357447	9.4737360
C	6.3019642	4.8030325	10.1700049
H	6.9213211	5.4239826	9.5203183
H	6.4548097	3.7549719	9.9246051
H	6.6339379	4.9819351	11.1954276
C	1.5629515	3.5884655	9.0451242
H	0.9875723	3.2854286	9.9250704
H	2.0471725	2.7068111	8.6309806
H	0.8569102	4.0031234	8.3272328

9) $[Co(III)(acac)_2(L2^{\cdot+})]^{2+}$

84
Energy = -3292.321355562

Co	1.6607388	10.4343193	8.1847975
O	0.6466483	11.1178481	1.8407356
O	2.6593225	9.9766748	1.8180372
O	2.5142227	12.1396937	8.1652929
O	0.4266095	10.9060966	9.5349223
O	2.9026982	9.9237094	9.5134698
O	0.8077491	8.7298003	8.1208466
N	2.8014666	9.8643965	6.6774723
N	5.0049781	9.3667292	7.3922586
N	3.8095788	7.7008657	6.5820016
N	0.5116559	11.0498124	6.7014047

N	-0.4982805	13.2150426	6.6807979
N	-1.6876804	11.5229911	7.4442375
C	2.2881080	10.1225287	5.4504746
C	1.0227062	10.8365735	5.4646863
C	0.3993320	11.2135949	4.2577044
H	-0.5513794	11.7263779	4.2443335
C	1.0358081	10.8747359	3.0926127
C	2.2714807	10.1720934	3.0786610
C	2.9093882	9.7898008	4.2294279
H	3.8600310	9.2778191	4.1957531
C	3.8436140	9.0073048	6.8585123
C	5.8418358	8.1884717	7.6476402
H	5.8703933	7.9850448	8.7213659
H	6.8592687	8.3606396	7.3006042
C	5.1147820	7.0844323	6.8572522
H	5.6177276	6.8400018	5.9182034
H	4.9862322	6.1672205	7.4299779
C	5.3831355	10.6922084	7.8449047
H	4.6372613	11.4088797	7.5159595
H	6.3609520	10.9461309	7.4344330
H	5.4252359	10.7151271	8.9345438
C	2.7065093	6.9536343	6.0109641
H	1.7892584	7.5256852	6.1159967
H	2.5990699	6.0134650	6.5526433
H	2.8868408	6.7341758	4.9559748
C	-0.5296336	11.9002688	6.9155084
C	-1.8022883	13.8210818	6.9833962
H	-2.3109988	14.0951424	6.0557193
H	-1.6709299	14.7195298	7.5845401
C	-2.5238442	12.6916523	7.7422111
H	-2.5462619	12.8605374	8.8220475
H	-3.5431813	12.5297782	7.3957924
C	0.6012311	13.9823138	6.1298989
H	0.7112863	14.9031607	6.7033295
H	0.4148408	14.2381405	5.0842189
H	1.5193620	13.4076810	6.2096951
C	-2.0621836	10.1835050	7.8568704
H	-3.0424922	9.9420281	7.4448238
H	-2.0972968	10.1259588	8.9454996
H	-1.3180737	9.4781954	7.5005678
C	1.6525164	10.5643872	0.9264085
C	1.0217176	9.4688502	0.1039400
H	0.5949512	8.7060250	0.7545266
H	1.7757706	9.0134015	-0.5368404
H	0.2338309	9.8886841	-0.5202258
C	2.2824112	11.6904544	0.1454808
H	1.5276839	12.1698035	-0.4768297
H	3.0697410	11.2947398	-0.4949197
H	2.7096996	12.4281014	0.8241400
C	2.3946046	13.0101912	9.0908287
C	1.4798494	12.9448392	10.1451927
H	1.4800085	13.7471091	10.8665406
C	0.5445949	11.9220284	10.3020915
C	3.3400767	14.1731597	8.9856543
H	3.0340573	15.0154608	9.6022518
H	3.4343596	14.4875886	7.9460932

H	4.3297127	13.8464257	9.3171208
C	-0.4372702	11.9564289	11.4384711
H	-0.4110674	12.8968861	11.9838674
H	-0.2060171	11.1396352	12.1265108
H	-1.4463980	11.7775272	11.0634825
C	2.7885012	8.8865997	10.2524672
C	1.8519484	7.8689793	10.0719942
H	1.8555158	7.0465154	10.7702370
C	0.9320185	7.8333824	9.0206311
C	3.7770809	8.8198049	11.3815742
H	3.7525470	7.8648764	11.9012939
H	3.5515720	9.6179808	12.0929506
H	4.7842750	9.0070794	11.0054003
C	-0.0138060	6.6735903	8.8872053
H	-0.1160637	6.3910120	7.8393003
H	-1.0009144	6.9893927	9.2363523
H	0.2973807	5.8130632	9.4754100

10) $[Co(II)(acac)_2(L3^{2+})]^{2+}$

100
Energy = -3451.984737409

Co	8.1200603	12.1800945	10.7619063
O	9.1614712	11.6879283	17.3230770
O	6.5081732	10.8438209	10.6631104
O	9.1176553	10.9146607	9.5682694
N	9.1747252	11.4313841	12.5210861
N	10.5087263	9.4865685	12.6779538
H	11.3590651	9.0242273	12.3885173
N	11.2874752	11.4187508	11.6391389
H	10.9700103	12.3386386	11.3002911
C	8.7729090	11.7960808	13.7004724
C	9.4366045	11.5299815	14.9385508
H	10.3984737	11.0400876	14.9656770
C	8.7777800	11.8671741	16.0721770
C	10.3686245	10.7513424	12.2962591
C	9.4378104	8.5781588	13.1612896
H	8.8384260	9.1417660	13.8760566
C	8.5557218	8.1306401	12.0003306
H	8.1041120	8.9856625	11.5012488
H	7.7584014	7.4833433	12.3678729
H	9.1375565	7.5683982	11.2673756
C	10.1029746	7.4167283	13.8898616
H	10.7303664	6.8351811	13.2090453
H	9.3417102	6.7444893	14.2840471
H	10.7184957	7.7627872	14.7216202
C	12.5145516	10.8361119	11.0593960
H	12.2596447	9.8230624	10.7272139
C	12.8962788	11.6472498	9.8279669
H	12.0674638	11.6892471	9.1206669
H	13.7525908	11.1874016	9.3357169
H	13.1784924	12.6629482	10.1102740

C	13.6359826	10.7748571	12.0958545
H	13.9154621	11.7807841	12.4127848
H	14.5155885	10.2958493	11.6652162
H	13.3464793	10.2092125	12.9841021
C	8.1196809	12.1796778	18.2334973
C	8.6662745	13.3353374	19.0316353
H	9.4814457	12.9859452	19.6639857
H	7.8790539	13.7439742	19.6639055
H	9.0366842	14.1160690	18.3678529
C	6.3305525	9.9779377	9.7373319
C	7.3018978	9.5883140	8.8095913
H	7.0178326	8.8392583	8.0855212
C	8.6249176	10.0494761	8.7731824
C	4.9666455	9.3428032	9.6659083
H	4.5161912	9.3087984	10.6577399
H	5.0010940	8.3391935	9.2452939
H	4.3245121	9.9476771	9.0201935
C	9.5796517	9.4917565	7.7525887
H	9.9418228	10.3086277	7.1243353
H	9.1320419	8.7276141	7.1210521
H	10.4486325	9.0713353	8.2640320
O	7.0780021	12.6714709	17.3229786
N	7.0654935	12.9288203	12.5211157
N	5.7314815	14.8735988	12.6779806
H	4.8811654	15.3359815	12.3885421
N	4.9526289	12.9413269	11.6393992
H	5.2701480	12.0215131	11.3004245
C	7.4671505	12.5637886	13.7004420
C	6.8032408	12.8297210	14.9384336
H	5.8413746	13.3196337	14.9654601
C	7.4618727	12.4923483	16.0721156
C	5.8715413	13.6088033	12.2963586
C	6.8024056	15.7819018	13.1615242
H	7.4013637	15.2183203	13.8766785
C	7.6850752	16.2289568	12.0008315
H	8.1367579	15.3737276	11.5021692
H	8.4823331	16.8762230	12.3685546
H	7.1036502	16.7911043	11.2674820
C	6.1371941	16.9436458	13.8895509
H	5.5102694	17.5252009	13.2083088
H	6.8984537	17.6157798	14.2839275
H	5.5212014	16.5979458	14.7211067
C	3.7255321	13.5239288	11.0596736
H	3.9803529	14.5370674	10.7276861
C	3.3439738	12.7129659	9.8280701
H	4.1728383	12.6712042	9.1208095
H	2.4876410	13.1727981	9.3358458
H	3.0618756	11.6971754	10.1101634
C	2.6040077	13.5848940	12.0960470
H	2.3245878	12.5788822	12.4127628
H	1.7243994	14.0639062	11.6654202
H	2.8933702	14.1503962	12.9844299
C	7.5729896	11.0239709	19.0315173
H	6.7577579	11.3733363	19.6638064
H	8.3601428	10.6152946	19.6638480
H	7.2026391	10.2432750	18.3676612

O	9.7319878	13.5162765	10.6627748
O	7.1222815	13.4455412	9.5683618
C	9.9094609	14.3822080	9.7370127
C	8.9379277	14.7719640	8.8095244
H	9.2218553	15.5211056	8.0854903
C	7.6148788	14.3108652	8.7733480
C	11.2733935	15.0172489	9.6653630
H	11.7238425	15.0516156	10.6571799
H	11.2390355	16.0206974	9.2443564
H	11.9154852	14.4120417	9.0199078
C	6.6599557	14.8688162	7.7530474
H	6.2977680	14.0521077	7.1245915
H	7.1074155	15.6331820	7.1216776
H	5.7910055	15.2890077	8.2647346

11) $[Co(III)(acac)_2(L3^{\cdot +})]^{2+}$

100
Energy = -3451.991317744

Co	8.1196793	12.1796765	10.8722998
O	9.0730869	11.5223541	17.2210803
O	6.7727065	10.8222247	10.8972172
O	9.1544676	11.3413419	9.5201962
N	9.0809928	11.3339895	12.3579560
N	10.3457194	9.3474357	12.5241974
H	11.2362389	8.8933563	12.3756802
N	11.2288550	11.2635544	11.5589128
H	10.9964802	12.2245147	11.3265354
C	8.6889978	11.7270613	13.5919178
C	9.2928691	11.3389193	14.8062991
H	10.1699597	10.7115340	14.8315517
C	8.7038410	11.7749877	15.9631397
C	10.2429709	10.6205780	12.1574387
C	9.2438620	8.4505469	12.9434156
H	8.3951709	9.0930144	13.1691431
C	8.8797978	7.5199988	11.7884454
H	8.5949898	8.0870125	10.9021429
H	8.0436577	6.8809936	12.0750743
H	9.7197546	6.8701669	11.5313361
C	9.6621146	7.6853413	14.1949182
H	10.5366756	7.0602387	13.9976300
H	8.8540227	7.0259873	14.5116010
H	9.8953580	8.3571606	15.0206710
C	12.4540678	10.6813777	10.9825397
H	12.1829173	9.6939194	10.5920092
C	12.8914059	11.5571986	9.8133209
H	12.0890761	11.6635201	9.0825119
H	13.7570900	11.1168718	9.3199408
H	13.1822152	12.5495274	10.1670169
C	13.5482281	10.5334704	12.0401703
H	13.8430406	11.5123279	12.4216924
H	14.4270524	10.0568684	11.6051356
H	13.2241932	9.9281055	12.8893039

C	8.1217549	12.1808027	18.1227693
C	8.8487569	13.2310587	18.9251599
H	9.5978479	12.7548393	19.5566070
H	8.1395474	13.7636645	19.5576657
H	9.3400459	13.9406379	18.2600187
C	6.6296753	9.9433132	9.9765450
C	7.5211841	9.7226204	8.9283628
H	7.2816230	8.9455074	8.2194500
C	8.7318981	10.4023938	8.7671900
C	5.3834516	9.1133469	10.0936281
H	5.2308324	8.8199205	11.1326863
H	5.4129309	8.2288492	9.4613817
H	4.5256304	9.7213087	9.7928637
C	9.6680262	10.0281345	7.6537844
H	9.8708827	10.9112297	7.0448938
H	9.2702321	9.2368652	7.0231238
H	10.6211239	9.7027508	8.0765143
O	7.1698818	12.8390297	17.2214723
N	7.1583984	13.0252086	12.3581832
N	5.8934030	15.0115826	12.5254523
H	5.0027566	15.4654939	12.3771937
N	5.0107279	13.0959601	11.5588018
H	5.2430523	12.1350281	11.3262699
C	7.5513453	12.6328921	13.5921153
C	6.9483121	13.0214019	14.8067815
H	6.0711719	13.6486848	14.8324735
C	7.5382200	12.5858442	15.9633787
C	5.9964609	13.7386520	12.1579095
C	6.9951783	15.9087676	12.9442531
H	7.8439036	15.2665063	13.1704341
C	7.3593144	16.8387322	11.7888316
H	7.6442376	16.2712852	10.9028433
H	8.1953989	17.4779287	12.0752052
H	6.5193451	17.4883865	11.5313182
C	6.5767760	16.6746141	14.1953138
H	5.7021907	17.2995483	13.9975957
H	7.3847908	17.3341996	14.5117030
H	6.3434950	16.0032493	15.0214225
C	3.7858454	13.6784426	10.9820489
H	4.0573407	14.6659090	10.5917785
C	3.3488047	12.8028543	9.8125359
H	4.1514310	12.6964521	9.0820667
H	2.4834304	13.2434232	9.3188284
H	3.0576277	11.8105520	10.1660022
C	2.6912570	13.8264107	12.0392309
H	2.3961596	12.8475679	12.4205687
H	1.8126784	14.3031477	11.6038462
H	3.0149692	14.4316792	12.8885570
C	7.3952107	11.1307484	18.9258440
H	6.6464859	11.6071206	19.5576080
H	8.1047853	10.5982808	19.5580563
H	6.9035312	10.4210180	18.2611514
O	9.4666734	13.5371488	10.8975445
O	7.0849958	13.0182646	9.5202447
C	9.6097965	14.4161648	9.9769807
C	8.7183863	14.6370018	8.9287536

H	8.9580520	15.4141599	8.2199265
C	7.5076086	13.9573564	8.7674559
C	10.8557709	15.2464361	10.0945650
H	11.0077178	15.5401378	11.1336487
H	10.8264527	16.1307907	9.4621138
H	11.7139092	14.6386003	9.7944648
C	6.5710825	14.3325708	7.6547000
H	6.3658710	13.4494799	7.0466222
H	6.9696979	15.1227292	7.0231621
H	5.6190689	14.6601368	8.0782259

6 References

[S1] D. F. Schrempp, E. Kaifer, H. Wadepohl, H.-J. Himmel, *Chem. Eur. J.* **2016**, *22*, 16187–16199.

[S2] (a) *DENZO-SMN*, Z. Otwinowski & W. Minor, Processing of X-ray Diffraction Data Collected in Oscillation Mode, Methods Enzymol. (1997), 276, Eds C. W. Carter, R. M. Sweet, Academic Press., (b) *SAINT*, Bruker AXS GmbH, Karlsruhe, Germany **2016.**

[S3] (a) G. M. Sheldrick, SADABS, Bruker AXS GmbH, Karlsruhe, Germany **2004-2014**; (b) L. Krause, R. Herbst-Irmer, G. M. Sheldrick, D. Stalke, J*. Appl. Cryst*. **2015**, *48*, 3.

[S4] a) G. M. Sheldrick, SHELXT, *Program for Crystal Structure Solution*, University of Göttingen, Germany **2014-2018**; b) G. M. Sheldrick, *Acta Cryst*. **2015**, *A71*, 3.

[S5] a) G. M. Sheldrick, *SHELXL-20xx*, University of Göttingen and Bruker AXS GmbH, Karlsruhe,Germany **2012-2018**; (b) W. Robinson, G. M. Sheldrick in: N. W. Isaaks, M. R. Taylor (eds.) „*Crystallographic Computing 4*“, Ch. 22, IUCr and Oxford University Press, Oxford, UK, **1988**; (c) G. M. Sheldrick, *Acta Cryst*. **2008**, *A64*, 112; (d) G. M. Sheldrick, *Acta Cryst*. **2015**, *C71*, 3.

[S6] O. V. Dolomanov, L. J. Bourhis, R. J. Gildea, J. A. K. Howard, H. Puschmann, OLEX2: A complete structure solution, refinement and analysis program, *J. Appl. Cryst.* **2009**, *42*, 339.

[S7] TURBOMOLE V7.1 **2016**, a development of University of Karlsruhe and Forschungszentrum Karlsruhe GmbH, 1989-2007, TURBOMOLE GmbH since **2007**.

[S8] a) P.J. Stephens, F. J. Devlin, C. F. Chabalowski, M. J. Frisch, *J. Phys. Chem*. **1994**, *98*, 11623-11627; b) A. D: Becke, *J. Chem. Phys*. **1993**, *98*, 5648–5652; c) C. Lee, W. Yang, R. G. Parr, *Phys. Rev. B* **1988**, *37*, 785–789.

[S9] A. Schäfer, H. Horn, R. Ahlrichs, *J. Chem. Phys*. **1992**, *97*, 2571.

[S10] K. Eichkorn, O. Treutler, H. Öhm, M. Häser and R. Ahlrichs, *Chem. Phys. Lett*. **1995**, *42*, 652–660.

[S11] M. Sierka, A. Hogekamp, R. Ahlrichs, *J. Chem. Phys*. **2003**, *118*, 9136–9148

[S12] S. Grimme, J. A., S. Ehrlich, H. Krieg, *J. Chem. Phys.* **2010**, *132*, 154104-1–154104-19.

[S13] A. E. Reed, R. B. Weinstock, F. Weinhold, *J. Chem. Phys*. **1985**, *83*, 735–746.

[S14] A. Wagner, H.-J. Himmel, *J. Chem. Inf. Model* **2017**, *57*, 428–438.
